# Open science discovery of potent noncovalent SARS-CoV-2 main protease inhibitors

**DOI:** 10.1126/science.abo7201

**Published:** 2023-11-10

**Authors:** Melissa L. Boby, Daren Fearon, Matteo Ferla, Mihajlo Filep, Lizbé Koekemoer, Matthew C. Robinson, The COVID Moonshot Consortium, John D. Chodera, Alpha A. Lee, Nir London, Annette von Delft, Frank von Delft

**Affiliations:** 1 Pharmacology Graduate Program, Weill Cornell Graduate School of Medical Sciences, New York, NY 10065, USA; 2 Program in Chemical Biology, Sloan Kettering Institute, Memorial Sloan Kettering Cancer Center, New York, NY 10065, USA; 3 Program in Computational and Systems Biology, Sloan Kettering Institute, Memorial Sloan Kettering Cancer Center, New York, NY 10065, USA; 4 Diamond Light Source Ltd., Harwell Science and Innovation Campus, Didcot, UK; 5 Research Complex at Harwell, Harwell Science and Innovation Campus, Didcot, UK; 6 Oxford Biomedical Research Centre, National Institute for Health Research, University of Oxford, Oxford, UK; 7 Department of Chemical and Structural Biology, The Weizmann Institute of Science, Rehovot, Israel; 8 Centre for Medicines Discovery, Nuffield Department of Medicine, University of Oxford, Oxford, UK; 9 Structural Genomics Consortium, Nuffield Department of Medicine, University of Oxford, Oxford, UK; 10 PostEra Inc., Cambridge, MA 02142, USA; 11 Department of Biochemistry, University of Johannesburg, Auckland Park, Johannesburg 2006, South Africa

## Abstract

**INTRODUCTION:**

COVID-19 became a global pandemic partially as a result of the lack of easily deployable, broad-spectrum oral antivirals, which complicated its containment. Even endemically, and with effective vaccinations, it will continue to cause acute disease, death, and long-term sequelae globally unless there are accessible treatments. COVID-19 is not an isolated event but instead is the latest example of a viral pandemic threat to human health. Therefore, antiviral discovery and development should be a key pillar of pandemic preparedness efforts.

**RATIONALE:**

One route to accelerate antiviral drug discovery is the establishment of open knowledge bases, the development of effective technology infrastructures, and the discovery of multiple potent antivirals suitable as starting points for the development of therapeutics. In this work, we report the results of the COVID Moonshot—a fully open science, crowd-sourced, and structure-enabled drug discovery campaign—against the severe acute respiratory syndrome coronavirus 2 (SARS-CoV-2) main protease (Mpro). This collaboration may serve as a roadmap for the potential development of future antivirals.

**RESULTS:**

On the basis of the results of a crystallographic fragment screen, we crowdsourced design ideas to progress from fragment to lead compounds. The crowdsourcing strategy yielded several key compounds along the optimization trajectory, including the starting compound of what became the primary lead series. Three additional chemically distinct lead series were also explored, spanning a diversity of chemotypes.

The collaborative and highly automated nature of the COVID Moonshot Consortium resulted in >18,000 compound designs, >2400 synthesized compounds, >490 ligand-bound x-ray structures, >22,000 alchemical free-energy calculations, and >10,000 biochemical measurements—all of which were made publicly available in real time. The recently approved antiviral ensitrelvir was identified in part based on crystallographic data from the COVID Moonshot Consortium.

This campaign led to the discovery of a potent [median inhibitory concentration (IC_50_) = 37 ± 2 nM] and differentiated (noncovalent and nonpeptidic) lead compound that also exhibited potent cellular activity, with a median effective concentration (EC_50_) of 64 nM in A549-ACE2-TMPRSS2 cells and 126 nM in HeLa-ACE2 cells without measurable cytotoxicity. Although the pharmacokinetics of the reported compound is not yet optimal for therapeutic development, it is a promising starting point for further antiviral discovery and development.

**CONCLUSION:**

The success of the COVID Moonshot project in producing potent antivirals building open knowledge bases, accelerating external discovery efforts, and functioning as a useful information-exchange hub is an example of the potential effectiveness of open science antiviral discovery programs. The open science, patent-free nature of the project enabled a large number of collaborators to provide in-kind support, including synthesis, assays, and in vitro and in vivo experiments. By making all data immediately available and ensuring that all compounds are purchasable from Enamine without the need for materials transfer agreements, we aim to accelerate research globally along parallel tracks. In the process, we generated a detailed map of the structural plasticity of Mpro, extensive structure-activity relationships for multiple chemotypes, and a wealth of biochemical activity data to spur further research into antivirals and discovery methodologies. We hope that this can serve as an alternative model for antiviral discovery and future pandemic preparedness.

Further, the project also showcases the role of machine learning, computational chemistry, and high-throughput structural biology as force multipliers in drug design. Artificial intelligence and machine learning algorithms help accelerate chemical synthesis while balancing multiple competing molecular properties. The design-make-test-analyze cycle was accelerated by these algorithms combined with planetary-scale biomolecular simulations of protein-ligand interactions and rapid structure determination.

## Abstract (summary)

We report the results of the COVID Moonshot, a fully open-science, crowd–sourced, and structure-enabled drug discovery campaign targeting the severe acute respiratory syndrome coronavirus 2 (SARS-CoV-2) main protease. We discovered a noncovalent, nonpeptidic inhibitor scaffold with lead-like properties that is differentiated from current main protease inhibitors. Our approach leveraged crowdsourcing, machine learning, exascale molecular simulations, and high-throughput structural biology and chemistry. We generated a detailed map of the structural plasticity of the SARS-CoV-2 main protease, extensive structure-activity relationships for multiple chemotypes, and a wealth of biochemical activity data. All compound designs (>18,000 designs), crystallographic data (>490 ligand-bound x-ray structures), assay data (>10,000 measurements), and synthesized molecules (>2400 compounds) for this campaign were shared rapidly and openly, creating a rich, open, and intellectual property–free knowledge base for future anticoronavirus drug discovery.

## Graphical Abstract

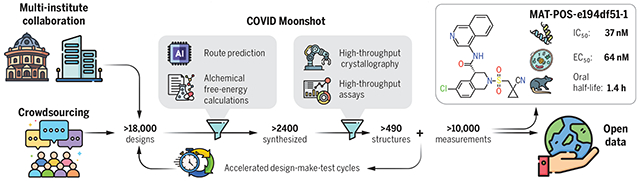

**The COVID Moonshot Consortium.** An open science, crowdsourced drug discovery campaign against the SARS-CoV-2 Mpro led to a potent, noncovalent, and nonpeptidic inhibitor scaffold with lead-like properties. We generated copious structural, biochemical, and pharmacological data that were shared rapidly and openly, creating a rich, open, and intellectual property–free knowledge base for future anticoronavirus drug discovery.

Despite rapid progress in vaccine development, the global failure to abate COVID-19, which culminated in more than 690 million confirmed cases worldwide by July 2023, will likely cause the virus to become endemic (1) and continue to cause a significant number of deaths, especially in the Global South, unless there is an accessible treatment (2). Antiviral therapeutics are a necessary and complementary strategy to vaccination to control COVID-19 (3). Several directly acting oral antivirals are now approved for use against severe acute respiratory syndrome coronavirus 2 (SARS-CoV-2) infection, including ritonavir-boosted nirmatrelvir (4), ensitrelvir (Japan) (5), and molnupiravir (6).

COVID-19 is not an isolated event but rather the latest exemplar of a series of threats to human health caused by beta-coronaviruses, which have also been responsible for the SARS (2003) and Middle East respiratory syndrome (MERS) (2010) pandemics (7). Open knowledge bases and technology infrastructures for antiviral drug discovery will enable pandemic preparedness by refreshing the antivirals pipeline and providing multiple starting points for the development of therapeutics. In this work, we report the open science discovery of a potent SARS-CoV-2 antiviral lead compound and a roadmap for the potential development of future SARS-CoV-2 and pan-coronavirus antivirals.

The SARS-CoV-2 main protease (Mpro; or 3CL-protease) is an attractive target for antiviral development because of its essential role in viral replication, a large degree of conservation across coronaviruses, and dissimilarity of its structure and substrate profile to human proteases (8) (fig. S1). Pioneering studies during and after the 2003 SARS pandemic established the linkage between Mpro inhibition and antiviral activity in cell culture (9). This work has been corroborated by in vitro and in vivo studies for SARS-CoV-2 (10, 11) and the clinical success of nirmatrelvir (the Mpro inhibitor component of Paxlovid) (12) and ensitrevir (Xocova) (13, 14).

To warrant early use in the course of disease or even prophylactically among at-risk populations, an antiviral drug would need to be orally available with an excellent safety profile. Given the historical difficulties in developing peptidomimetic compounds into oral drugs and the risk of downstream idiosyncratic hazards of covalent inhibition, we chose to pursue noncovalent, nonpeptidomimetic scaffolds. First–generation oral Mpro inhibitors have now demonstrated clinical efficacy (15, 16), but the need for cytochrome P450 3A4 (CYP3A4) inhibitor co-dosing (ritonavir, in the case of Paxlovid) to achieve sufficient human exposure may substantially limit use in at-risk populations because of drug-drug interactions (17). There remains a need for chemically differentiated oral antiviral protease inhibitors with the potential to enter clinical development.

## Crowdsourced progression of x-ray fragment hits rapidly generated potent lead compounds with diverse chemotypes

**Fig. 1.**
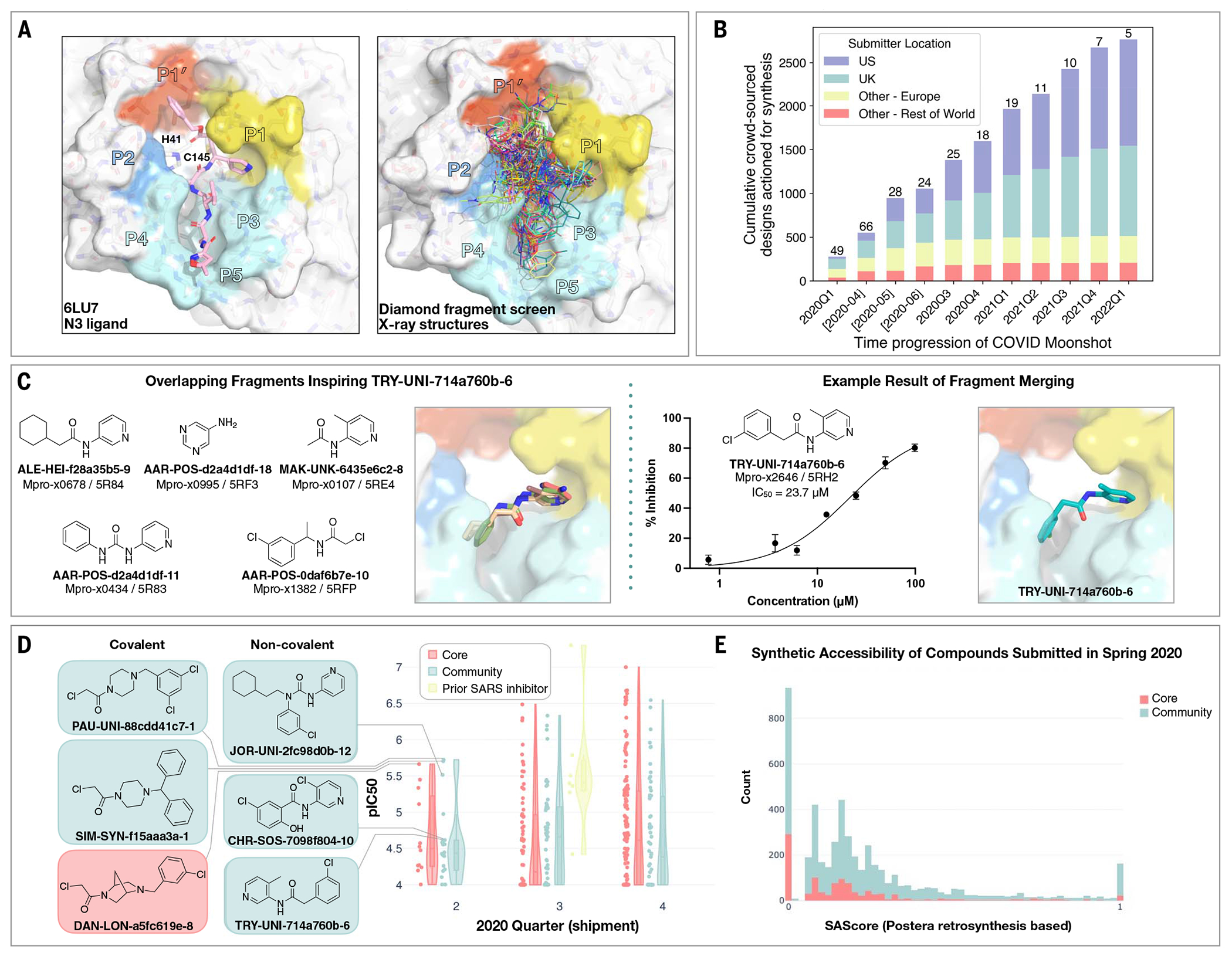
Crowdsourcing rapidly identified chemotype scaffolds by merging fragment hits. (**A**) A Diamond/XChem fragment screen that initiated this SARS-CoV-2 Mpro inhibitor discovery campaign generated 71 hits that completely cover the Mpro active site, with a variety of chemotypes engaging each pocket; 1638 x-ray datasets were collected, and 96 solved structures for hits were publicly posted (20). The peptidomimetic N3 ligand is shown on the left for comparison to indicate natural substrate engagement in the binding site, defining the peptide sidechain numbering scheme used throughout this work. The nucleophilic Cys^145^ reacts with the scissile peptide bond between P1 and P1’; His^41^-Cys^145^ form a catalytic dyad whose coupled charge states shuttle between zwitterionic and neutral states (90). (**B**) On 18 March 2020, the COVID Moonshot set up a crowdsourcing website to empower scientists across the globe to contribute molecule designs. The number of designs actioned for synthesis each quarter (except for the 2020 Q2, which is shown per-month in brackets) is shown, subdivided by the region of the submitter of the design idea. The total number of unique submitters that contributed actioned designs for that quarter is shown on top of the bars. (**C**) Many submissions, such as TRY-UNI-714a760b-6, exploited spatially overlapping fragment hits to design potent leads that are synthetically facile. (**D**) Experimental biochemical potency of designs broken down by submission group. Multiple submissions in 2020 from the community were more potent than the best designs from the core team, as seen for the top three chloroacetamide structures (left) and noncovalent structures (right). (**E**) Distribution of synthetic accessibility scores (SAScores) for designs contributed by the core team and the community. The concern that community submissions may be of poor quality is not supported by the fact that these were as synthetically accessible as those designed by the core team (median: community, 0.17; core, 0.13). Half of the outliers (SAScore = 1) were primarily natural products, which are hard to achieve through organic chemistry.

The COVID Moonshot is an open science drug discovery campaign targeting SARS-CoV-2 Mpro (18, 19), building off a rapid crystallographic fragment screening campaign that assessed >1250 unique fragment-soaked crystals screened within weeks to identify 71 hits that densely populated the active site (Fig. 1A) (20). This dataset was posted online on 18 Mar 2020 (21), days after the screen was completed (21). The noncovalent fragment hits did not show detectable inhibition in a fluorescence-based enzyme activity assay [assay dynamic range median inhibitory concentration (IC_50_) < 100 μM]. However, they provided a high-resolution map of key interactions that optimized compounds may exploit to inhibit Mpro (22).

Numerous approaches have been proposed to advance from fragments to lead compounds (23, 24). One strategy, fragment merging, aims to combine multiple fragments into a single, more-potent molecule, whereas fragment expansion elaborates a fragment to engage neighboring interactions. Although these strategies are usually applied to a single fragment or a handful of fragments, our large-scale fragment screen produced a dense ensemble of hits, which provided an opportunity for rapid lead generation by combining chemotypes from multiple fragments. Nonetheless, this approach requires heuristic chemical reasoning that accounts for the spatial orientation of fragments in the binding site—a feat that can challenge algorithms but is also potentially solvable by humans. Building on successes in crowdsourced protein (25) and RNA (26) design campaigns, we hypothesized that crowdsourced human analysis and algorithmic strategies could accelerate the generation of potent lead compounds and furnish diverse chemical matter because different chemists would use different approaches and reasoning strategies.

We launched an online crowdsourcing platform (https://postera.ai/covid) on 18 March 2020 (Fig. 1B), soliciting participants to submit compounds designed on the basis of the fragment hits (19). Compounds selected for synthesis were evaluated by biochemical assays (data S1) and x-ray crystallography, and the results were released rapidly on the same platform, which enabled contributing designers to build on all available data as well as on designs contributed by others. To facilitate transparency and maximal speed, and to avoid delays around intellectual property (IP), all designers were asked to contribute their designs directly into the public domain, with every design and all related experimental data immediately disclosed online and made openly available, explicitly free of IP restrictions. This aggressive open science policy enabled contributors from multiple fields in both academia and industry to freely share their ideas. Within the first week, we received more than 2000 submissions, representing a diverse set of design strategies (data S2).

Many submissions exploited spatially overlapping fragment hits. For example, the submission TRY-UNI-714a760b-6 was inspired by five overlapping fragments, furnishing a noncovalent inhibitor with a SARS-CoV-2 Mpro enzymatic IC_50_ of 23.7 μM (Fig. 1C). This compound seeded the aminopyridine series, whose optimization is described in detail below. Only 11 of the 768 fragments in the DSi-poised library (27, 28) contained a 3-amino pyridine; yet, four of them were successfully identified in the crystallographic fragment screen and were consequently picked up for merging by the designers. Apart from the aminopyridine series, our campaign identified three other major chemically distinct lead series with measurable potencies against SARS-CoV-2 Mpro inspired by reported SARS-CoV-1 inhibitors (fig. S2). Those compounds span the same binding pocket but feature different chemotypes, and the large quantity of structure-activity relationship (SAR) subsequently generated for these series furnishes multiple backup series with different risk profiles. Other groups have subsequently further elaborated on the Ugi (29, 30) and the benzotriazole series that we generated (31).

Analysis of the submissions provides some hints to the utility of crowdsourcing as a general strategy for hit-discovery or hit-to-lead campaigns. A qualitative assessment of the textual description of submitted designs (fig. S3) hints that many of the designers used tools such as docking to assess fragment “linking,” “merging,” or “combination.” When trying to more-thoroughly categorize submissions, it does not appear that hypothesis-driven designs perform better than docking-driven designs; however, “predicting” historical SARS inhibitors is the best-performing strategy (fig. S4 and Fig. 1D). Throughout the campaign, designs were contributed both by the core group of laboratories and medicinal chemists leading this project and by the community. One could hypothesize that the core group being committed to the project, as well as being thoroughly invested in the campaign details, would contribute more-potent designs. However, there is no obvious difference in the distributions of designs produced by the core group versus the community in the early stages of the campaign (Fig. 1D), nor were the designs contributed by the community less synthetically accessible (Fig. 1E). Later in the campaign (lead optimization stage), the number of submissions from the community decreased, and comparing potency became irrelevant as other attributes of the molecules were being optimized. It is important to mention that several key compounds along the optimization trajectory of our lead were contributed by the community and not core group members: TRY-UNI-714a760b-6, ADA-UCB-6c2cb422-1, and VLA-UCB-1dbca3b4-15 (the racemic mixture of MAT-POS-b3e365b9-1). Although anecdotal, this example demonstrates the potential power of crowdsourcing as a strategy to drive fragment-to-lead campaigns.

## Technologies to support rapid optimization cycles

With a growing number of chemically diverse submissions, we relied on a team of experienced medicinal chemists supported by computational methods to aid in triaging design proposals with the goal of increasing potency. To execute a rapid fragment-to-lead campaign, we used models to plan synthetic routes, enumerate synthetically accessible virtual libraries, and estimate potencies to prioritize which compounds to target for synthesis. We did not use an “autonomous” approach—expert judgment is used to make decisions given all the model predictions. Furthermore, in the context of a fast-moving campaign, we prioritized making progress over granular “human-versus-machine” evaluations.

### Synthetic route predictions guided decision-making to accelerate design-make-test-analyze cycles

**Fig. 2.**
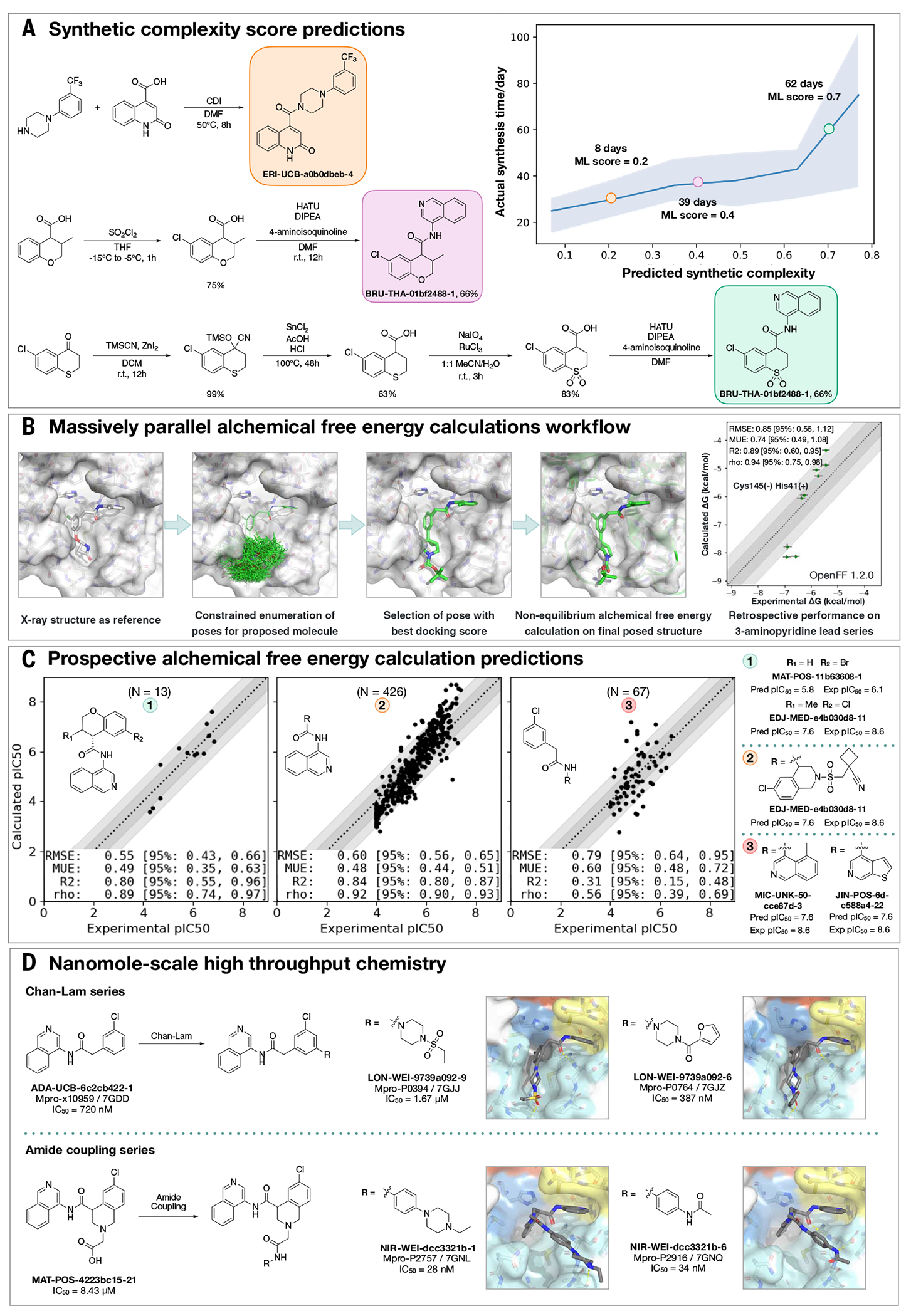
Strategies to support rapid optimization cycles. (**A**) Machine learning forecasts experimental synthesis time (left) and returns efficient routes that leverage more than 10 million in-stock advanced intermediates (right). Our algorithm predicts the probability of each step being successful and predicts synthetic accessibility by taking the product of the probabilities along the whole route. We analyzed all compounds made in COVID Moonshot from 1 May 2020 to 1 July 2021 (*n* = 898). The right panel exemplifies the experimental execution of the predicted routes, demonstrating the ability of the algorithm to build on functionalized intermediates to shorten synthesis. (**B**) Applying alchemical free-energy calculations at scale enables us to estimate the potency of compounds. Retrospective assessment of our automated free-energy calculation workflow on early compounds in the 3-aminopyridine series in the first month of the COVID Moonshot campaign suggested that free-energy calculations could provide good predictive utility, which inspired confidence for large-scale deployment during this campaign. Here, the absolute free energy of binding (ΔG) is shown in the rightmost panel by adding a constant offset to the computed relative free-energy differences. (**C**) Alchemical free-energy predictions for all submissions elaborating on the depicted scaffold for three representative batches of prospective free-energy calculations plotted as calculated (converted using Cheng-Prusoff equation) versus experimental pIC_50_. Simulations were run using Mpro in dimer form, with neutral catalytic dyad and no restraints. Each batch (numbered 1 to 3 from left to right) is annotated with its scaffold, and top-scoring candidates are shown on the right-hand side (numbered 1 to 3 from top to bottom)–for these, the structure names are shown together with their predicted and experimental pIC_50_ (“Pred” and “Exp,” respectively). Statistical performance with 95% confidence intervals for each batch is shown as a table in each scatterplot. (**D**) Two examples of nanomole-scale HTC campaigns used to optimize the potency of intermediate binders, centering on the Chan-Lam reaction (fig. S7) and amide couplings (fig. S8). Direct biochemical screening of crude reactions identified candidates that were resynthesized and in both cases were able to improve the potency of the parent compound. Soaking of crude reaction mixtures of the most potent biochemical hits into Mpro crystals provided complex structures with the identified hits (Chan-Lam PDBs: 7GJJ/7GJZ, resolution: 1.75Å/1.65Å; Amide coupling PDBs: 7GNL/7GNQ, resolution: 1.68Å/1.53Å). In both cases, new interactions were discovered, explaining the improved activity. Although for the Chan-Lam reaction campaign, the extended compounds occupied the intended P4, for the amide-coupling vector, all compounds extended into the P3/5 pockets.

We used an established synthetic contract research organization (CRO), Enamine, to carry out rapid synthesis of progressed compound designs. To take full advantage of the available building block collection, we used a machine learning approach that plans efficient retrosynthetic routes to predict synthetic tractability (32, 33). We automatically computed synthetic routes for all crowdsourced submissions using Enamine’s in-stock building block inventories. From the computed routes, synthetic complexity was estimated on the basis of the number of steps and the probability of success of each step. The synthetic accessibility score, as well as the predicted synthetic route, were then used to aid medicinal chemistry decision-making. Our predicted synthetic complexity correlated with the actual time taken to synthesize target compounds, and the algorithm was able to pick out advanced intermediates as starting materials (Fig. 2A).

### Alchemical free-energy calculations prioritized potent compounds for synthesis

We estimated potency of proposed designs and virtual synthetic libraries of analogs using alchemical free-energy calculations (34–36), an accurate physical modeling technique that has hitherto not been deployed in a high-throughput setup because of its prohibitive computational cost. We used Folding@home (37)—a worldwide distributed computing network where hundreds of thousands of volunteers around the world contributed computing power to create the world’s first exascale computing resource (38)—to compute the free energy of binding of all 20,000+ crowdsourced and internal design submissions using the Open Force Field Initiative “Parsley” small-molecule force fields (39) and nonequilibrium switching with the open source PERSES alchemical free-energy toolkit (40–42) based on the graphics processing unit (GPU)–accelerated OpenMM framework (38, 43) (see Materials and methods). Comprehensive sampling was prioritized over efficiency of computation given the abundant compute resources available on Folding@home.

We first performed a small retrospective study using bioactivity data generated from the first week of crowdsourced compound designs, triaged solely using synthetic accessibility. The results of these free-energy calculations showed good correlation with experimentally measured affinities (Fig. 2B). Henceforth, alchemical free-energy calculations were used as an additional (though not the sole) criterion to guide compound selection and iterative design (see Data and materials availability statement). During the campaign, distinct objectives were solicited from submitters to address medicinal chemistry problems, and free-energy calculations were used to assess these submissions on the basis of predicted potency. Fig. 2C shows that predicted −log_10_ IC_50_ (pIC_50_) tracks experimental measurements across three chronologically distinct design campaigns: decoration of the benzopyran ring, replacement of the benzopyran system, and replacement of the isoquinoline system. Some design ideas with low predicted pIC_50_ were synthesized because the medicinal chemistry team balanced between gaining insights on structure-activity and structure-property relationship and potency optimization. The champion compounds from each design campaign are highlighted in the right panel of Fig. 2C. Although free-energy calculations identified multiple potency-improving transformations, the strategically useful one was the swap from pyran to a piperidine sulfonamide system, which is on the critical path to the lead compound. On average, 80 GPU-hours per compound were used across the three panels (Materials and methods).

A major strength of alchemical free-energy calculations proved to be their ability to select potent analogs from virtual synthetic libraries from which the medicinal chemistry team had already selected compounds sharing a common intermediate as well as highlighting submitted designs predicted to be highly potent but where major synthetic effort would be required. Our design team prioritized for synthesis small libraries suggested by the aforementioned computational approaches. Chemically related groups of outliers frequently provided chemical insight that informed modeling choices (fig. S5). The approach was not without drawbacks, including the need to focus on a single reference compound and structure to design transformation networks (rather than leveraging the abundant structural data), the requirement that protonation states be fixed for the entire calculation (requiring the entire transformation network to be recomputed to assess a different protonation state), and the relatively large computational cost required to handle large replacements (see Materials and methods). The method is also not uniformly accurate across all chemical transformations, and accurately estimating its accuracy beforehand is challenging. For example, isoquinoline replacements show lower correlation between calculated and predicted free energy (Fig. 2B, panel 3) compared with the benzopyran replacements (Fig. 2B, panel 2).

### Nanomole-scale high-throughput chemistry enabled rapid evaluation of SAR

A complementary method for rapid SAR evaluation was the use of nanomole-scale high-throughput chemistry (HTC) (44, 45) coupled with a “direct to biology” (46–48) biochemical screening. Two examples include the optimization of the Chan-Lam reaction (49) to extend molecule ADA-UCB-6c2cb422-1 and amide coupling to extend MAT-POS-4223bc15-21 (Fig. 2D). In both cases, we determined the cocrystal structures of the parent compounds (fig. S6) and suggested vectors that could target the P4 pocket of Mpro. Optimization of the reaction conditions was performed for the starting building block with model amines (figs. S7 and S8), and the optimal conditions were applied to HTC with a library of 300 amine building blocks (data S3). Yield estimation was performed in both cases and showed for the Chan-Lam library that only 29 of the library yielded >30% of the desired product compared with 151 for the amide coupling. Nevertheless, the crude mixtures were subjected to a biochemical assay against Mpro (data S3). Seven compounds were selected for resynthesis from the Chan-Lam series and 20 from the amide series (fig. S9). In parallel to synthesis, the crude reaction mixtures were subjected to soaking and x-ray crystallography. The structures verified that the extended compounds do adopt a similar binding mode to the parent. Chan-Lam–extended compounds occupied P4, whereas the amides extended toward P3/P5, in both cases forming new interactions with Mpro (Fig. 2D). Upon resynthesis, one of the Chan-Lam compounds was able to slightly improve over the parent compound IC_50_. Several of the amide-coupling series were able to improve by up to 300-fold on the parent acid-compound (up to threefold on the corresponding methylamide), with the best inhibitor exhibiting an IC_50_ of 28 nM against Mpro.

### Covalent targeting strategies

Another approach that was attempted to rapidly gain potency was the use of electrophiles to covalently target the catalytic C145. The original fragment screen (20) that launched this effort included electrophiles (50) and resulted in 48 structures of covalently bound fragments, the majority of which were chloroacetamides. Some of the earliest, and most-potent, fragment merges explored by both the core group and the community were of chloroacetamide (Fig. 1D), and further optimization improved chloroacetamide fragments’ IC_50_ values to as low as 300 nM (fig. S10). Chloroacetamides, however, are not considered suitable for therapeutics, and therefore we aimed to move toward acrylamides by derivatizing potent reversible hits (30) (fig. S11). Ultimately, we focused on a noncovalent series, but the chlorophenyl moiety that remained throughout the series was adopted from a chloroacetamide hit fragment (AAR-POS-0daf6b7e-10; Fig. 1C).

## High-throughput structural biology uncovered binding modes and interactions underlying potency

**Fig. 3.**
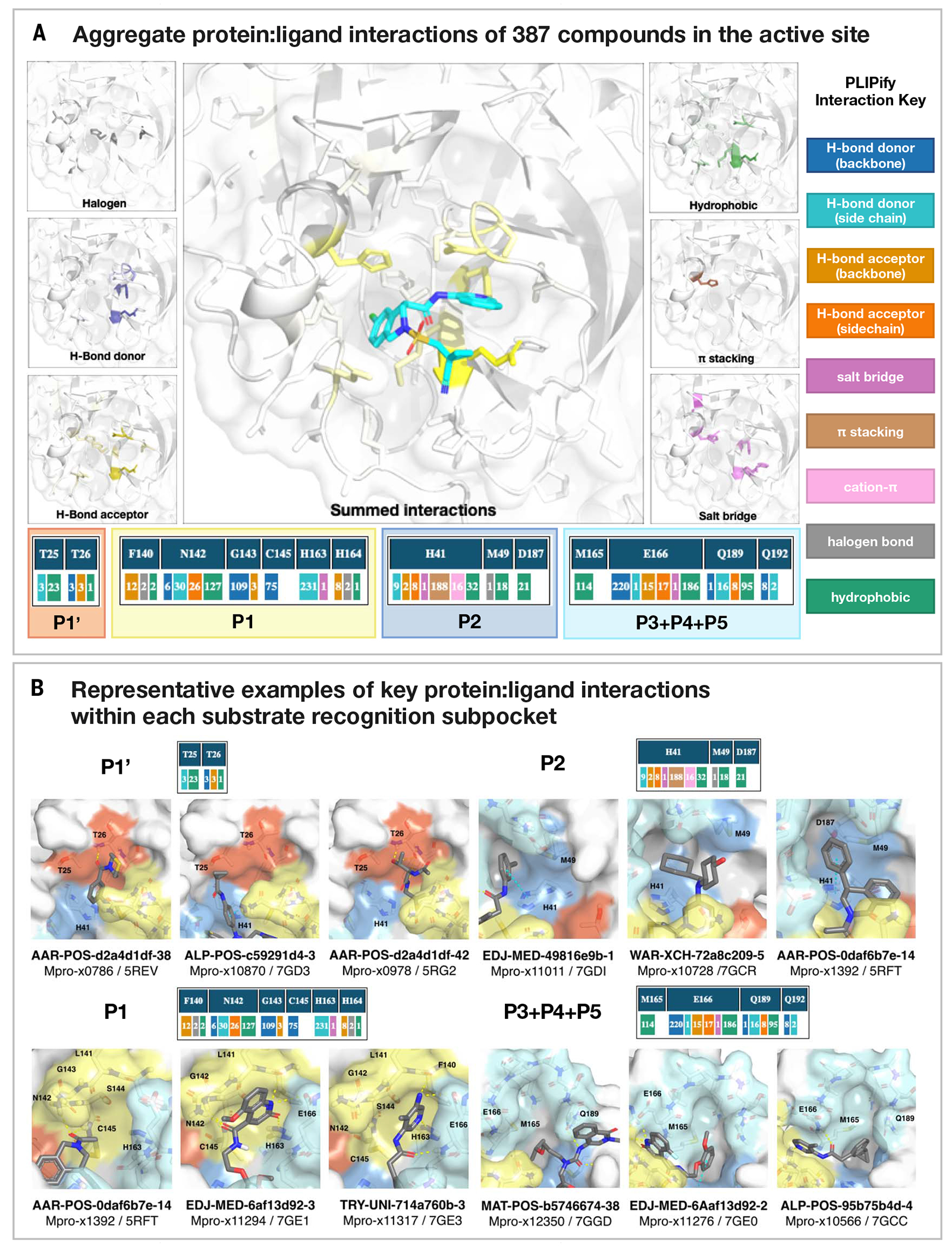
Analysis of 367 complex crystal structures reveals hotspots for ligand engagement and a variety of ways to engage substrate recognition subpockets. (**A**) The five substrate recognition subpockets exhibit distinct preferences for intermolecular interactions. The figure highlights the locations of different types of interactions, with the shading indicating the frequency. The bottom row tallies the number of times that each interaction was seen in our structures for different residues. The interaction map was generated using PLIPify (Materials and methods) and summarizes the interactions witnessed across 367 complexes from the perspective of the protein, distinguishing between backbone (bb) and sidechain (sc) interactions (which might be more vulnerable to point mutations). (**B**) Representative examples of protein-ligand interactions engaging the P1’, P1, P2, and P3-5 subpockets. Hydrogen bonds and π-stacking interactions are depicted as yellow and cyan dashed lines, respectively. The rows above each set of complexes tally the number of times that each interaction was seen with the specific residues within the subpockets. See data S4 for Protein Data Bank (PDB) IDs and crystallography statistics. Single-letter abbreviations for the amino acid residues are as follows: A, Ala; C, Cys; D, Asp; E, Glu; F, Phe; G, Gly; H, His; I, Ile; K, Lys; L, Leu; M, Met; N, Asn; P, Pro; Q, Gln; R, Arg; S, Ser; T, Thr; V, Val; W, Trp; and Y, Tyr.

We selected compounds on the basis of synthetic tractability and alchemical free-energy calculations. We profiled every compound through crystal soaking and x-ray diffraction, totaling in 587 structures (see table S1 and fig. S12 for average statistics, data S4 for crystallographic and refinement statistics, and fig. S13 for ligand density for the structures highlighted in this manuscript). Analysis of a subset of this large trove of structural data (*n* = 367, up to July 2021) reveals the hotspots for ligand engagement and plasticity of each binding pocket. Fig. 3 highlights the statistics of intermolecular interactions between the residues and our ligands. The P1 and P2 pockets are the hotspots of interactions; yet, the interaction patterns are starkly different. The salient interactions sampled by our ligands in the P1 pocket are H163 (H-bond donor), E166 (H-bond acceptor), and N142 (hydrophobic interactions), whereas P2 interactions are dominated by π-stacking interactions with H41 and hydrophobic interactions with M165. The P1’ and P3/4/5 pockets are sparingly sampled by our ligands; the former can be targeted through hydrophobic interactions (T25) and the latter through H bonds (Q192).

**Fig. 4.**
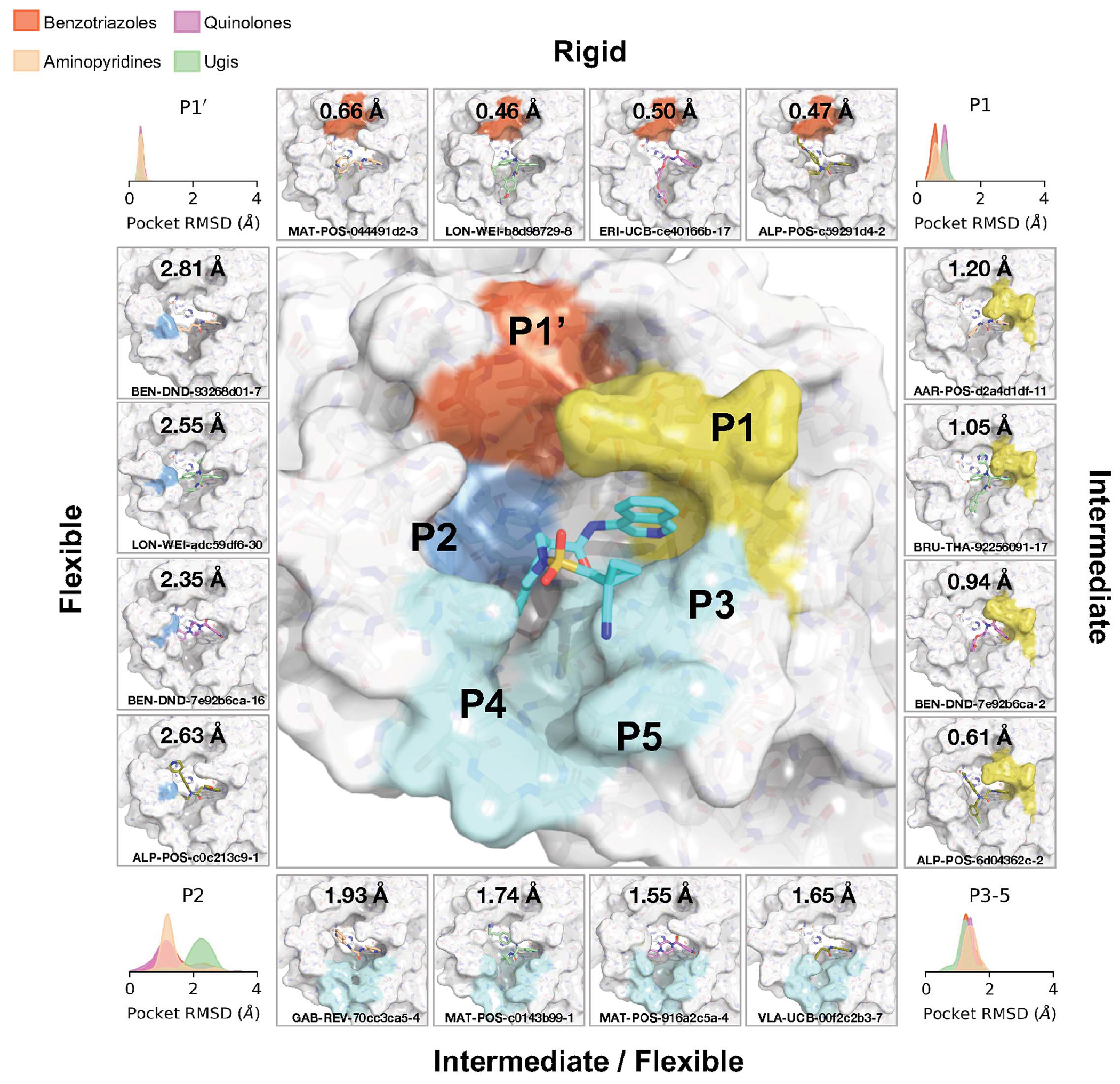
Structural plasticity of the binding subpockets. The subpockets have different degrees of plasticity, which is also dependent on the chemical series (fig. S2). The corners of the figure show the distribution of sidechain root mean square deviation (RMSD) deviations from the structure of MAT-POS-e194df51-1 (middle panel; PDB: 7GAW). The boxes exemplify ligands that significantly deform the pockets.

This pattern of intermolecular interactions is reflected in the plasticity of the different subpockets. The dominance of directional interactions in P1 renders it more rigid than P2 (Fig. 4). The rigidity is also dependent on the chemical series (fig. S2), with the Ugi and benzotriazole series being able to deform the P2 pocket. Those series comprise more heavy atoms and span a larger region of the binding site; thus, changes in P2 pocket interactions could be better tolerated.

## Design of a SARS-CoV-2 Mpro inhibitor lead series with potent antiviral activity

**Fig. 5.**
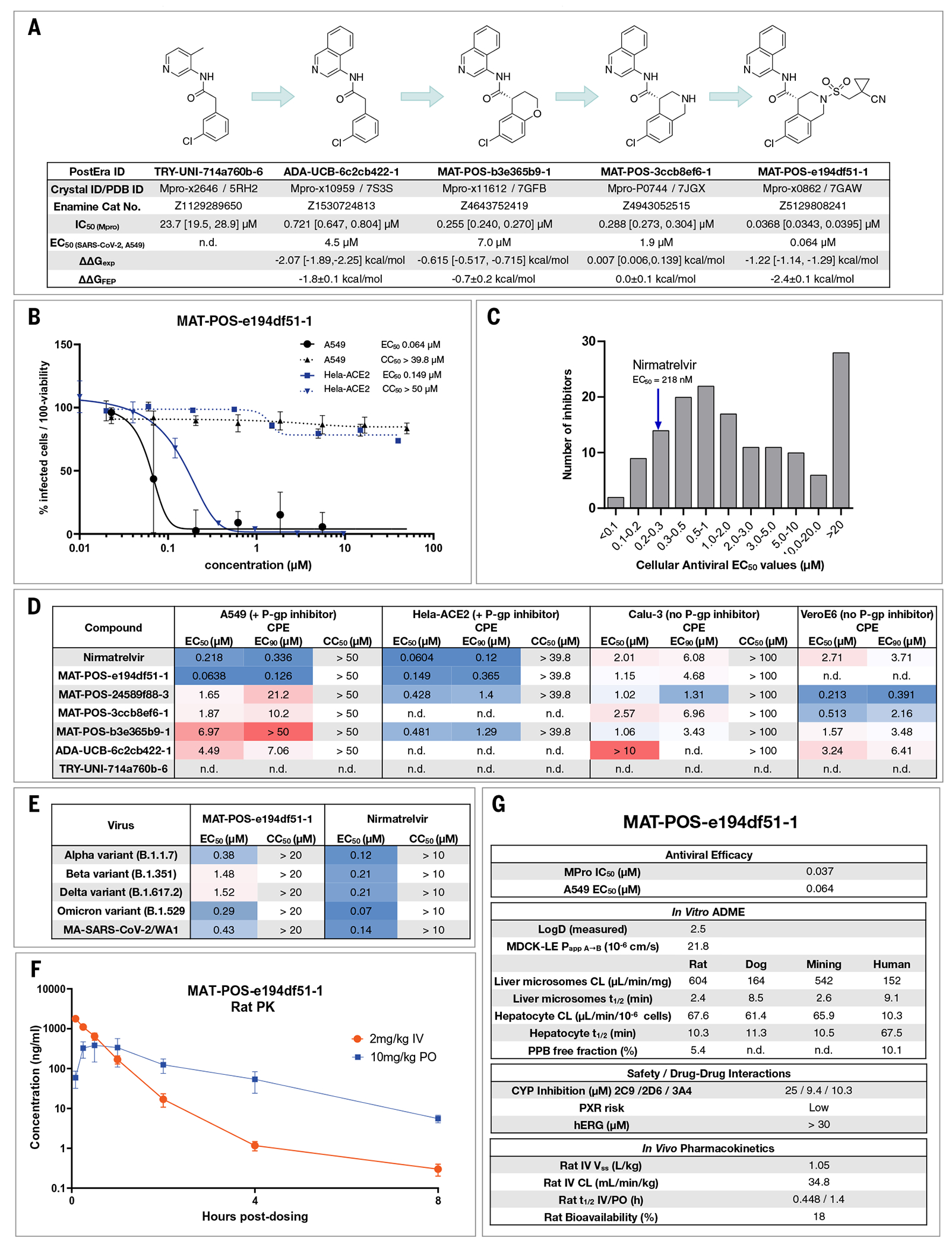
Iterative medicinal chemistry furnished an orally bioavailable inhibitor. (**A**) Summary of key medicinal chemistry milestones in developing the initial crowdsourced lead compound into a potent antiviral. X-ray structures for each milestone compound are available via Fragalysis, and each compound can be obtained from Enamine using the corresponding catalog numbers. Retrospective alchemical free-energy calculation predictions for each transformation (ΔΔG_FEP_) are shown for each step between milestones, along with the corresponding experimental free-energy difference (ΔΔG_exp_) derived from biochemical activities. As positive control, under our assay condition, nirmatrelvir has an IC_50_ of 2.6 nM. (**B**) Antiviral activity of MAT-POS-e194df51-1 cellular antiviral assays, with an EC_50_ of 64 nM in A549-ACE2-TMPRSS2 cells assessing CPE (black; plotted as 100 – percent viability) and 126 nM in HeLa-ACE2 assays (blue; plotted as percentage of infected cells). Both assays were performed with P-gp inhibitors. (**C**) Histogram comparing antiviral efficacy of all COVID Moonshot compounds measured to date in an A549-ACE2-TMPRSS2 CPE cellular antiviral assay. (**D**) Detailed cellular antiviral assessment of key compounds composing the synthetic strategy (A) across different cell lines and assay techniques, with and without p-gp inhibitors, demonstrating efficacy of MAT-POS-e194df51-1 in various setups and laboratories. (**E**) MAT-POS-e194df51-1 shows good cross-reactivity against known circulating variants of SARS-CoV-2 in antiviral cellular assays in a CPE assay in HeLa-ACE2 cells. (**F**) PK profile of MAT-POS-e194df51-1 in rats with a 2 mg/kg intravenous and 10 mg/kg oral dosing with good oral availability. (**G**) ADME characteristics of MAT-POS-e194df51-1 demonstrate a favorable safety profile, indicating translational potential of the lead series.

Our medicinal chemistry strategy was driven by the design of potent ligand-efficient and geometrically compact inhibitors that fit tightly in the substrate binding pocket. The former strategy aimed to increase the probability of achieving oral bioavailability, whereas the latter heuristic was motivated by the substrate envelope hypothesis for avoiding viral resistance (51). Fig. 5A outlines the critical intermediates on the path toward an optimized lead compound.

Starting from the fragment hit, we explored the P1 pocket, which admits a steep SAR—perhaps unsurprising given its rigidity and preference for directional H-bond interactions (Fig. 3A). An increase in potency was unlocked by replacing pyridine with isoquinoline, which picks up additional hydrophobic interactions with N142. The SAR around the P2 pocket is considerably more tolerant to modifications and broadly favors hydrophobic moieties. A step-change in potency was achieved by rigidifying the scaffold: We introduced a tetrahydropyran ring to transform the P2 substituent into a chromane moiety (compound MAT-POS-b3e365b9-1; the racemic mixture VLA-UCB-1dbca3b4-15, which was initially synthesized, has a IC_50_ of 360 nM; Fig. 5A), chosen because of building block availability. Despite having a degree of molecular complexity, MAT-POS-b3e365b9-1 is obtained through a one-step amide coupling (Fig. 2A). We then further explored the P2 pocket with a library chemistry strategy in mind. Thus, guided by free-energy calculations (Fig. 2C), we first substituted the chromane for a tetrahydroisoquinoline to introduce a functionalizable handle (MAT-POS-3ccb8ef6-1; Fig. 5A), which maintained potency. Finally, we constructed a focused library realized through sulphonamide Schotten-Baumann coupling (fig. S14), furnishing an increase in both enzymatic inhibition and cellular antiviral efficacy. This work led to a potent antiviral chemical series (Fig. 5A) with a favorable safety profile, low brain penetrance (fig. S15 and data S5), and improved oral bioavailability but moderate in vitro–in vivo correlation in clearance (fig. S16 and data S5; all measured cellular antiviral data are available in data S6).

As an example for the aminopyridine lead series, we discuss antiviral efficacy, absorption, distribution, metabolism, and excretion (ADME) and pharmacokinetic (PK) characteristics of compound MAT-POS-e194df51-1. MAT-POS-e194df51-1 was profiled in SARS-CoV-2 antiviral assays across multiple cell lines, exhibiting a median effective concentration (EC_50_) of 64 nM in A549-ACE2-TMPRSS2 cells and 126 nM in HeLa-ACE2 cells without measurable cytotoxicity (Fig. 5B). This is in line with overall cellular efficacy for the chemical series: Of 150 compounds with enzyme assay IC_50_ < 500 nM assessed in A549-ACE2-TMPRSS2 cellular cytophatic effect (CPE) assays, 15 compounds showed lower EC_50_ values compared with the internal control nirmatrelvir that was measured at an EC_50_ of 218 nM in this assay (Fig. 5C). Similarly, good antiviral activity was measured across “crowdsourced” antiviral assays across different laboratories and cell lines, including assays performed with and without p-gp inhibitors and using nirmatrelvir as an internal control (Fig. 5D). We also observed good cross-reactivity of our lead compound MAT-POS-e194df51-1 against known SARS-CoV-2 variants Alpha, Beta, Delta, and Omicron (Fig. 5E). Closely related molecules PET-UNK-29afea89-2 and MAT-POS-932d1078-3 with EC_50_ values in HeLa-ACE2 CPE assays of 240 nM and 331 nM and with values of 657 nM and 2.57 μM in A549-ACE2-TMPRSS2 CPE assays, respectively (fig. S17, A and B), show a >100-fold reduction of intracellular viral RNA and infectious virus secretion into the apical compartment of human induced pluripotent stem cell (iPSC)–derived kidney organoids (fig. S16, D and E)—an accessible model for the human kidney, an organ that is infected in COVID-19 patients—as reported previously for earlier analogs of the same series (52). MAT-POS-e194df51-1 exhibits favorable properties required for an orally bioavailable inhibitor (Fig. 5, F and G). In addition, crystallographic studies reveal that the interaction pattern of MAT-POS-e194df51-1 with the Mpro binding site is distinct to approved Mpro inhibitors nirmatrelvir and ensitrelvir (S-217622) (fig. S18), potentially offering complementary resistance profiles and justifying further development.

## Open science presents a viable route to early drug discovery

The results presented here reflect the success of an open science, patent-free antiviral discovery program in rapidly developing a differentiated optimized lead in response to an emerging pandemic threat. As a result of the open science policy, a large number of collaborators (now the COVID Moonshot Consortium) were able to provide in-kind support, providing synthesis, assays, and in vitro and in vivo experiments. By making all data immediately available and all compounds purchasable from Enamine, we aim to accelerate research globally along parallel tracks following up on our initial work. As a notable example for the impact of open science, the Shionogi clinical candidate S-217622 [which has now received emergency approval in Japan as Xocova (ensitrelvir)] was identified in part on the basis of crystallographic data openly shared by the COVID Moonshot Consortium (53).

Despite our optimization and characterization efforts, considerable gaps from reporting a clinical candidate remain: The series requires further PK and pharmacodynamic (PD) optimization; in particular, it displays high clearance and low bioavailability. As it stands, it would likely not be able to achieve therapeutic exposure without a PK booster (such as ritonavir). To move forward, additional in-depth safety data are required as well as additional PK data from a second species to enable accurate human dose prediction. The COVID Moonshot and its lead series for COVID-19 have been adopted into the drug development portfolio of the Drugs for Neglected Diseases initiative (DND*i*) for further lead optimization and downstream preclinical development This work is funded by a $10 million award from the Wellcome Trust through the World Health Organization (WHO) Access to COVID-19 Tools Accelerator (ACT-A) program, of which results will be reported upon filing Clinical Trials Authorization (CTA) (54). To reach phase 2 readiness, we expect a further $7.5 million will be required to process route development costs (55).

Open science efforts have transformed many areas of biosciences, with examples such as the Human Genome Project (56), the Structural Genomics Consortium (57), and the RAS Initiative (58). The COVID Moonshot provides an example of open science drug discovery leading to advances in infectious diseases drug discovery—a research area of grave public importance, but one that is chronically underfunded by the private sector (59).

## Materials and methods

### 0. Compound registration and data flow process

All compound designs from the internal medicinal chemistry team, collaborators, and external submitters were captured through the online compound design submission platform (https://covid.postera.ai/covid) along with submitter identity, institution, design rationale, and any inspiration fragments. A forum thread was created to discuss these designs and attached to the compound design. Each submitted batch of related designs received a unique ID including the first three letters of the submitter name and submitter institution, and each compound design submitted received a unique ID (“PostEra ID”) that appended a unique molecule sequence ID within the submission batch ID. Internally, compound designs, synthesized compounds, and compounds with experimental data were tracked with corresponding records in a CDD Vault (Collaborative Drug Discovery Inc.).

#### Stereochemistry

Although the design platform enabled submitters to register compounds with specific defined or uncertain stereochemistry, compounds were initially synthesized and biochemically assayed as racemates, and if active, chirally separated compounds were registered and assayed separately. Because the absolute stereochemical identity of enantiopure compounds was unknown at time of receipt, assay data were attached to compound records with specified relative stereochemistry, rather than absolute stereochemistry. For compounds where sufficient data were available from a variety of sources to propose the absolute stereochemistry (e.g., x-ray data for the compound or a close analog), the “suspected_SMILES” record was updated along with an articulated rationale in the “why_suspected_SMILES” field. As a result, caution must be exercised when using data for enantiopure compounds for downstream uses (e.g., whole-dataset machine learning) without verifying whether the absolute stereochemistry is known with confidence.

#### Submission analysis

The submitter names were standardized by removing affiliations and expansion of first name abbreviations, the submissions by two users who submitted large batches of compounds in an automated way in contravention of the goal of the project were removed. The word cloud was generated by filtering against 1000 most-common words and removing grammatical inflections and generating an image with an online word cloud generator. The classification of the methodology was done by presence of keywords determined by a simple keyword classifier with manually determined words (circa 100 training, 100 test) wherein “dock,” “seesar,” “vina,” “autodock,” “screen,” “drug-hunter” were typical of docking, whereas “by-eye,” “merg[ing],” “link[ing],” “coupl[ing]” were typical of hypothesis driven methods. A large fraction could not be accurately classified due to paucity of information. SAScore was calculated with Postera Manifold under the retrosynthesis route.

### 1. Experimental methods

#### 1.1 Protease activity assays

##### 1.1.1 Fluorescence Mpro inhibition assay

Compounds were seeded into assay-ready plates (Greiner 384 low volume, cat. no. 784900) using an Echo 555 acoustic dispenser, and dimethyl sulfoxide (DMSO) was back-filled for a uniform concentration in assay plates (DMSO concentration maximum 1%) Screening assays were performed in duplicate at 20 μM and 50 μM. Hits of greater than 50% inhibition at 50 μM were confirmed by dose response assays. Dose response assays were performed in 12-point dilutions of twofold, typically beginning at 100 μM. Highly active compounds were repeated in a similar fashion at lower concentrations beginning at 10 μM or 1 μM. Reagents for Mpro assay were dispensed into the assay plate in 10 μl volumes for a final volume of 20 μl.

Final reaction concentrations were 20 mM HEPES pH 7.3, 1.0 mM TCEP, 50 mM NaCl, 0.01% Tween-20, 10% glycerol, 5 nM Mpro, 375 nM fluorogenic peptide substrate ([5-FAM]-AVLQSGFR-[Lys(Dabcyl)]-K-amide). Mpro was pre-incubated for 15 min at room temperature with compound before addition of substrate and a further 30-min incubation. Protease reaction was measured in a BMG Pherastar FS with a 480/520 excitation/emission filter set. Raw data were mapped and normalized to high (Protease with DMSO) and low (No Protease) controls using Genedata Screener software. Normalized data were then uploaded to CDD Vault (Collaborative Drug Discovery). Dose response curves were generated for IC_50_ using nonlinear regression with the Levenberg–Marquardt algorithm with minimum inhibition = 0% and maximum inhibition = 100%.

The assay was calibrated at different enzyme concentrations to confirm linearity and response of protease activity, as well as optimization of buffer components for most stable and reproducible assay conditions. Substrate concentration was chosen after titration to minimize saturation of signal in the plate reader while obtaining a satisfactory and robust dynamic range of typically five- to sixfold over control without enzyme. We used low substrate concentrations of the bright FRET peptide to avoid “inner filter effect” (60) and to bias toward detection of competitive inhibitors (61). As positive control, under our assay condition, nirmatrelvir has IC_50_ of 2.6 nM.

##### 1.1.2 RapidFire Mpro inhibition assay

The assay was performed according to the published procedure (62). Briefly, compounds were seeded into assay-ready plates (Greiner 384PP, cat. no. 781280) using an ECHO 650T dispenser and DMSO was back-filled for a uniform concentration in assay plates (DMSO concentration < 1%, final volume = 500 nl.). A 15 μM enzyme stock solution was prepared in 20 mM HEPES, pH 7.5 and 300 mM NaCl, and subsequently diluted to a working solution of 300 nM Mpro in assay buffer (20 mM HEPES, pH 7.5 and 50 mM NaCl) before the addition of 25 μl to each well using a Multidrop Combi (Thermo Scientific). After a quick centrifugation step (1000 rpm, 15 s) the plate was incubated for 15 min at room temperature. The reaction is initiated with the addition of 25 μl of 4 μM 11-nucleotide oligomer (TSAVLQSGFRK-NH2, initially custom synthesized by the Schofield group, then by GLBiochem, used until March 2021), or 10 μM 37-nucleotide oligomer (ALNDFSNSGS-DVLYQPPQTSITSAVLQSGFRKMAFPS-NH2, GLBiochem, used after March 2021), dissolved in assay buffer. After centrifugation (1000 rpm, 14 s) the reaction is incubated for 10 min (11-nucleotide oligomer) or 5 min (37-nucleotide oligomer) at room temperature before quenching with 10% formic acid. The reactions are analyzed with MS using RapidFire (RF) 365 high-throughput sampling robot (Agilent) connected to an iFunnel Agilent 6550 accurate mass quadrupole time-of-flight (Q-TOF) mass spectrometer using electrospray. All compounds are triaged by testing the percentage inhibition at 5 and 50 μM final concentration. Dose response curves uses an 11-point range of 100 to 0.0017 μM inhibitor concentrations. RF integrator software (Agilent) was used to extract the charged states from the total ion chromatogram data followed by peak integration. For the 11-nucleotide oligomer peptide the m/z (+1) charge states of both the substrate (1191.67 Da) and cleaved N-terminal product TSAVLQ (617.34 Da) were used and the 37-nucleotide oligomer peptide the m/z (+2) charge states of the substrate (3960.94 Da) and m/z (+1) of the cleaved C-terminal product SGFRKMAFPS (1125.57 Da). Percentage conversion [(product peak integral)/(product peak integral + substrate peak integral) × 100] and percentage inhibitions were calculated and normalized against DMSO control with deduction of any background signal in Microsoft Excel. IC_50_ values were calculated using Levenberg–Marquardt algorithm used to fit a restrained Hill equation to the dose-response data with both GraphPad PRISM and CDD.

#### 1.2 High-throughput x-ray crystallography

Purified protein (20) at 24 mg/ml in 20 mM HEPES pH 7.5, 50 mM NaCl buffer was diluted to 12 mg/ml with 20 mM HEPES pH 7.5, 50 mM NaCl before performing crystallization using the sitting-drop vapor diffusion method with a reservoir solution containing 11% PEG 4 K, 5% DMSO, 0.1 M MES pH 6.5. Crystals of Mpro in the monoclinic crystal form (C2), with a single monomer in the asymmetric unit, were grown with drop ratios of 0.15 μl protein, 0.3 μl reservoir solution, and 0.05 μl seeds prepared from previously produced crystals of the same crystal form (20). Crystals in the orthorhombic crystal form (P2_1_2_1_2_1_), with the Mpro dimer present in the asymmetric unit, were grown with drop ratios of 0.15 μl protein, 0.15 μl reservoir solution, and 0.05 μl seeds prepared from crystals of an immature Mpro mutant in the same crystal form (63).

Compounds were soaked into crystals by adding compound stock solutions directly to the crystallization drops using an ECHO liquid handler. In brief, 40 to 90 nl of DMSO solutions (between 20 and 100 mM) were transferred directly to crystallization drops using giving a final compound concentration of 2 to 20 mM and DMSO concentration of 10 to 20%. Drops were incubated at room temperature for ~1 to 3 hours before mounting and flash cooling in liquid nitrogen without the addition of further cryoprotectant.

Data were collected at Diamond Light Source on the beamline I04-1 at 100 K and processed with the fully automated pipelines at Diamond (64–66), which include XDS (67), xia2 (68), autoPROC (69), and DIALS (64). Further analysis was performed using XChemExplorer (70) with electron density maps generated using DIMPLE (http://ccp4.github.io/dimple/). Ligand binding events were identified using PanDDA (77) (https://github.com/ConorFWild/pandda), and ligands were manually modeled into PanDDA-calculated event maps or electron density maps using Coot (72). Ligand restraints were calculated with ACEDRG (73) or GRADE [grade v. 1.2.19 (Global Phasing Ltd., Cambridge, UK, 2010)] and structures refined with Buster [Buster v. 2.10.13 (Cambridge, UK, 2017)]. Models and quality annotations were reviewed using XChemReview (74), Buster-Report [Buster v. 2.10.13 (Cambridge, UK, 2017)] and Mogul (75, 76).

Coordinates, structure factors and PanDDA event maps for all datasets are available on Fragalysis (https://fragalysis.diamond.ac.uk/viewer/react/preview/target/Mpro).

#### 1.3 Viral screening assays

A variety of antiviral replication assays were performed in collaborating laboratories, including cytopathic effect (CPE) inhibition assays at the IIBR, Israel, and Katholieke Universiteit Leuven; quantitative reverse-transcription polymerase chain reaction (RT-qPCR) for viral RNA at Radboud University Medical Center, Netherlands; immunofluorescence assays at University of Nebraska Medical Center, USA; and plaque assays and focus-forming unit (FFU) assays at University of Oxford, UK.

##### 1.3.1 Antiviral cytopathic effect assay, VeroE6 (IIBR, Ness-Ziona, Israel)

SARS-CoV-2 (GISAID accession EPI_ISL_406862) was kindly provided by Bundeswehr Institute of Microbiology, Munich, Germany. Virus stocks were propagated (4 passages) and tittered on Vero E6 cells. Handling and working with SARS-CoV-2 virus was conducted in a BSL3 facility in accordance with the biosafety guidelines of the Israel Institute for Biological Research (IIBR). Vero E6 were plated in 96-well plates and treated with compounds in medium containing 2% fetal bovine serum (FBS). The assay plates containing compound dilutions and cells were incubated for 1 hour at 37°C before adding multiplicity of infection (MOI) 0.01 of viruses. Viruses were added to the entire plate, including virus control wells that did not contain test compound and Remdesivir drug used as positive control. After 72 hours incubation, viral CPE inhibition assay was measured with XTT reagent. Three replicate plates were used.

##### 1.3.2 Antiviral immunofluorescence assay, VeroE6 (Pathology and Microbiology, University of Nebraska Medical Center, USA, St Patrick Reid)

Vero E6 cells were pretreated with 20 uM of the Moonshot compounds for around 2 hours. Cells were then infected with SARS-CoV-2 at a MOI of 0.1 for 24 hours. Virus infection was terminated by 4% paraformaldehyde (PFA) fixation. Cells were stained using a Rabbit SARS-CoV-2 antibody (Sino Biological 40150-R007) as a primary antibody, and Alexa-488, Hoechst and Cell Mask (Thermo Fisher) as a secondary antibody. Images were collected on the Operetta system imaging system and analyzed using the Harmony software.

##### 1.3.3 Antiviral FFU assay, Calu-3 (University of Oxford, UK)

###### Cell culture

The African green monkey Vero E6 cell line (ATCC CRL-1586) was cultured in Dulbecco’s modified Eagle medium (DMEM) with Glutamax supplemented with 100 μg/ml streptomycin, 100 U/ml penicillin, and 10% heat-inactivated fetal calf serum (FCS). The human lung cancer cell line Calu-3 (Anderson Ryan, Department of Oncology, Medical Science Division, University of Oxford) was cultured in a 1:1 mixture of DMEM with Glutamax and Ham’s F-12 medium supplemented with 100 μg/ml streptomycin, 100 U/ml penicillin, and 10% heat-inactivated FCS. All cells were maintained as mycoplasma free, with regular verifications by polymerase chain reaction (PCR).

###### Virus propagation

SARS-CoV-2 England/2/2020 was provided at passage 1 from Public Health England, Collindale. Passage 2 submaster and passage 3 working stocks were produced by infecting Vero E6 cells at a MOI of 0.01 in virus propagation medium (DMEM with Glutamax supplemented with 2% FCS) and incubating until CPE was visible. The cell supernatant was then centrifuged at 500 g for 5 min, aliquoted and stored at −80°C. The titer of viral stocks was determined by plaque assay. All subsequent assays were performed using a passage 3 stock.

###### Cell viability

Cell viability was measured using the CellTiter 96 R AQueous One Solution Cell Proliferation MTA [3-(4,5-dimethylthiazol-2-yl)-5-(3-carboxy-methoxyphenyl)-2-(4-sulfophenyl)-2H - 15 tetrazolium, inner salt] Assay (Promega) according to the manufacturer’s instruction after treatment with compound. Briefly, Calu 3 cells were treated with compounds in quadruplicate for 3 days. Wells with 200 μl growth medium with and without cells were included as controls in quadruplicate. After the incubation, 100 μl of growth medium was removed and 20 μl of MTS reagent was added to the remaining medium in each well. After a further 1- to 2-hour incubation, the absorbance at 490 nm was measured on a Molecular Devices SpectraMax M5 microplate reader.

###### Antiviral assays

For FFU assays, a SARS-CoV-2 Microneutralization assay from the W. James laboratory (Dunn School of Pathology, University of Oxford) was adapted for use as a FFU assay. Briefly, 3 half log dilutions of each supernatant to be analyzed were prepared in virus propagation medium. 20 μl of each dilution was inoculated into wells of a 96-well plate in quadruplicate followed by 100 μl Vero E6 cells at 4.5 × 10^5^ cells/ml in virus propagation medium. The plates were incubated for 2 hours before the addition of 100 μl of 1.8% CMC overlay, and then incubated for a further 24 hours. After 24 hours the overlay was carefully removed and the cells washed once with PBS before fixing with 50 μl of 4% PFA, after 30 min the PFA was removed and replaced with 100 μl of 1% ethanolamine in PBS. The cells were permeabilized by replacing the ethanolamine with 2% Triton X100 in PBS and incubating at 37°C for 30 min. The plates were then washed three times with wash buffer (0.1% Tween 20 in PBS) inverted and gently tapped onto tissue to dry before the addition of 50 μl of EY2A anti-N human monoclonal antibody (mAb) [Arthur Huang (Taiwan)/Alain Townsend (Weatherall Institute of Molecular Medicine, University of Oxford)] at 10 pmol in wash buffer. The plates were rocked at room temperature for 1 hour, washed and incubated with 100 μl of secondary antibody anti-human immunoglobulin G (IgG) (Fc-specific)-peroxidase-conjugate produced in Goat diluted 1:5000 at room temperature for 1 hour. 50 μl ofTrueBlue peroxidase substrate was added to the wells and incubated at RT for 10 min on the rocker, after 10 min the substrate was removed, and the plates washed with ddH2O for 10 min. The water was removed and the plates allowed to air dry. The foci were then counted using an ELISPOT classic reader system (AID GmbH).

##### 1.3.4 Antiviral qPCR assay, Vero E6 and kidney organoids (Radboud University Medical Center, Nijmegen, Netherlands)

###### Cell culture

African green monkey Vero E6 kidney cells (ATCC CRL-1586) and Vero FM kidney cells (ATCC CCL-81) were cultured in DMEM with 4.5 g/L glucose and L-glutamine (Gibco), supplemented with 10% FCS (Sigma Aldrich), 100 μg/ml streptomycin and 100 U/ml penicillin (Gibco). Cells were maintained at 37°C with 5% CO_2_. Human iPSC-derived kidney organoids were prepared as previously described (52).

###### Virus propagation

SARS-CoV-2 (isolate BetaCoV/Munich/BavPat1/2020) was kindly provided by C. Drosten (Charité-Universitätsmedizin Berlin, Institute of Virology, Berlin, Germany) and was initially cultured in Vero E6 cells up to three passages in the laboratory of Bart Haagmans (Viroscience Department, Erasmus Medical Center, Rotterdam, Netherlands). Vero FM cells were infected with passage 3 stock at an MOI of 0.01 in infection medium (DMEM containing L-glutamine, 2% FCS, 20 mM HEPES buffer, 100 μg/ml streptomycin and 100 U/ml penicillin). Cell culture supernatant containing virus was harvested at 48 hours postinfection (hpi), centrifuged to remove cellular debris, filtered using a 0.2 μm syringe filter (Whatman), and stored in 100 μl aliquots at −80°C.

###### Virus titration

Vero E6 cells were seeded in 12-well plates at a density of 500,000 cells per well. Cell culture medium was discarded at 24 hours postseeding, cells were washed twice with PBS and infected with 10-fold dilutions of the virus stock in unsupplemented DMEM. At 1 hpi, cells were washed with PBS and replaced with overlay medium, consisting of minimum essential medium (Gibco), 2% FCS, 20 mM HEPES buffer, 100 μg/ml streptomycin, 100 U/ml penicillin, and 0.75% carboxymethyl cellulose (Sigma Aldrich). At 72 hpi, the overlay medium was discarded, cells were washed with PBS and stained with 0.25% crystal violet solution containing 4% formaldehyde for 30 min. Afterward, staining solution was discarded and plates were washed with PBS, dried and plaques were counted.

###### Antiviral assay

Vero E6 cells were seeded onto 24-well plates at a density of 150,000 cells per well. At 24 hours postseeding, cell culture medium was discarded, cells were washed twice with PBS and infected with SARS-CoV-2 at an MOI of 0.01 in the presence of six concentrations of the inhibitors (25 μM to 0.06 μM). At 1 hpi, the inoculum was discarded, cells were washed with PBS, and infection medium containing the same concentration of the inhibitors was added to the wells. SARS-CoV-2 infection in the presence of 0.1% DMSO was used as a negative control. At 24 hpi, 100 μl of the cell culture supernatant was added to RNA-Solv reagent (Omega Bio-Tek) and RNA was isolated and precipitated in the presence of glycogen according to manufacturer’s instructions. TaqMan Reverse Transcription reagent and random hexamers (Applied Biosystems) were used for cDNA synthesis. Semi-quantitative real-time PCR was performed using GoTaq qPCR (Promega) BRYT Green Dye-based kit using primers targeting the SARS-CoV-2 E protein gene (77) (forward primer, 5′-ACAGGTACGTTAATAGTTAATAGCGT-3′; reverse primer, 5′-ACAGGTACGTTAATAGTTAATAGCGT-3′). A standard curve of a plasmid containing the E gene qPCR amplicon was used to convert Ct values relative genome copy numbers. For viability assays, Vero E6 cells were seeded in 96-well white-bottom culture plates (Perkin Elmer) at a density of 30,000 cells per well. At 24 hours postseeding, cells were treated with the same concentrations of compounds as used for the antiviral assay. Cells treated with 0.1% DMSO were used as a negative control. At 24 hours post-treatment, cell viability was assessed using the Cell Titer Glo 2.0 kit (Promega) using the Victor Multilabel Plate Reader (Perkin Elmer) to measure luminescence signal.

###### Antiviral assays in organoids

Human iPSC-derived kidney organoids cultured in transwell filters (Corning) were infected with SARS-CoV-2 in the presence of 1 and 10 μM of MAT-POS-932d1078-3, PET-UNK-29afea89-2 or 0.1% DMSO using an MOI of 1.0 in Essential 6 medium (Gibco) at 37°C and 5% CO_2_, exposing the cells both basolaterally and apically to the inoculum. After 24 hours, medium containing the inoculum was removed and fresh essential 6 medium containing the same concentration of inhibitor was added to the basolateral compartment and cells were cultured for an additional 24 hours. At 48 hpi, organoids were washed in PBS, and the apical surface was exposed to Essential 6 medium for 10 min at 37°C, which was collected and used for viral titration. Individual organoids were harvested for RNA isolation using the PureLink RNA mini kit (Thermo Fisher) according to manufacturer’s instructions. Viral RNA copies were analyzed by RT-qPCR on the SARS-CoV E gene, as described previously (78).

##### 1.3.5 High-content SARS-CoV-2 antiviral screening assay, HeLa-ACE2 (Takeda via Calibr/TSRI)

###### SARS-CoV-2/HeLa-ACE2 high-content screening assay

Compounds are acoustically transferred into 384-well μclear-bottom plates (Greiner, part no. 781090-2B) and HeLa-ACE2 cells are seeded in the plates in 2% FBS at a density of 1.0 × 10^3^ cells per well. Plated cells are transported to the BSL3 facility where SARS-CoV-2 (strain USA-WA1/2020 propagated in Vero E6 cells) diluted in assay media is added to achieve ~30 to 50% infected cells. Plates are incubated for 24 hours at 34°C 5% CO_2_, and then fixed with 8% formaldehyde. Fixed cells are stained with human polyclonal sera as the primary antibody, goat anti-human H+L conjugated Alexa 488 (Thermo Fisher Scientific A11013) as the secondary antibody, and antifade 4′,6-diamidino-2-phenylindole (DAPI) (Thermo Fisher Scientific D1306) to stain DNA, with PBS 0.05% Tween 20 washes in between fixation and subsequent primary and secondary antibody staining. Plates are imaged using the ImageXpress Micro Confocal High-Content Imaging System (Molecular Devices) with a 10× objective, with four fields imaged per well. Images are analyzed using the Multi-Wavelength Cell Scoring Application Module (MetaXpress), with DAPI staining identifying the host-cell nuclei (the total number of cells in the images) and the SARS-CoV-2 immunofluorescence signal leading to identification of infected cells.

###### Uninfected host cell cytotoxicity counter screen

Compounds are acoustically transferred into 1536-well plates (Corning no. 9006BC). HeLa-ACE2 cells are maintained as described for the infection assay and seeded in the assay-ready plates at 400 cells per well in DMEM with 2% FBS. Plates are incubated for 24 hours at 37°C 5% CO_2_. To assess cell viability, 2 ml of 50% Cell-Titer Glo (Promega no. G7573) diluted in water is added to the cells and luminescence measured on an EnVision Plate Reader (Perkin Elmer).

##### Data analysis

Primary in vitro screen and the host cell cytotoxicity counter screen data are uploaded to Genedata Screener, Version 16.0. Data are normalized to neutral (DMSO) minus inhibitor controls (2.5 μM remdesivir for antiviral effect and 10 μM puromycin for infected host cell toxicity). For the uninfected host cell cytotoxicity counter screen 40 μM puromycin (Sigma) is used as the positive control. For dose response experiments compounds are tested in technical triplicates on different assay plates and dose curves are fitted with the four parameter Hill Equation.

##### 1.3.6 Cytopathic effect assay, hACE2-TMPRSS2 cells (Katholieke Universiteit Leuven)

###### Virus isolation and virus stocks

All virus-related work was conducted in the high-containment BSL3 facilities of the KU Leuven Rega Institute (3CAPS) under licenses AMV 30112018 SBB 219 2018 0892 and AMV 23102017 SBB 219 2017 0589 according to institutional guidelines. The SARS-CoV-2 strain used for this study was the Alpha variant of Concern (derived from hCoV-19/Belgium/rega-12211513/2020; EPI_ISL_791333, 2020-12-21). Virus sample was originally isolated in-house from nasopharyngeal swabs taken from travelers returning to Belgium (baseline surveillance) and were subjected to sequencing on a MinION platform (Oxford Nanopore) directly from the nasopharyngeal swabs. Virus stocks were then grown on Vero E6 cells in (DMEM 2% FBS medium) and passaged one time on A549-ACE2-TMPRSS2 cells. Median tissue culture infectious doses (TCID50) was defined by endpoint titration.

###### A549-ACE2-TMPRSS2 assay

A549-Dual hACE2-TMPRSS2 cells obtained by Invitrogen (cat. no. a549d-cov2r) were cultured in DMEM 10% FCS (Hyclone) supplemented with 10 μg/ml blasticidin (Invivogen, ant-bl-05), 100 μg/ml hygromycin (Invivogen, ant-hg-1), 0.5 μg/ml puromycin (Invivogen, ant-pr-1) and 100 μg/ml zeocin (Invivogen, ant-zn-05). For antiviral assay, cells were seeded in assay medium (DMEM 2%) at a density of 15,000 cells per well. One day after, compounds were serially diluted in assay medium (DMEM supplemented with 2% v/v FCS) and cells were infected with their respective SARS-CoV-2 strain at a MOI of ~0.003 TCID50/ml. On day 4 pi., differences in cell viability caused by virus-induced CPE or by compound-specific side effects were analyzed using MTS as described previously (79). Cytotoxic effects caused by compound treatment alone were monitored in parallel plates containing mock-infected cells.

##### 1.3.6 Immunofluorescence SARS-CoV-2 antiviral screening assay, HeLa-ACE2 (Mount Sinai)

Assessment of cross-reactivity against SARS-CoV-2 variant strains and cytotoxicity assays were performed as previously described (80). In brief, two thousand HeLa-ACE2 cells (BPS Bioscience) were seeded into 96-well plates in DMEM (10% FBS) and incubated for 24 hours at 37°C, 5% CO2. Two hours before infection, the medium was replaced with 100 μl of DMEM (2% FBS) containing the compound of interest at concentrations 50% greater than those indicated, including a DMSO control. Plates were then transferred into the BSL3 facility and 100 PFU (MOI = 0.025) was added in 50 μl of DMEM (2% FBS), bringing the final compound concentration to those indicated. Plates were then incubated for 48 hours at 37°C. After infection, supernatants were removed, and cells were fixed with 4% formaldehyde for 24 hours before being removed from the BSL3 facility. The cells were then immunostained for the viral N protein (an inhouse mAb 1C7, provided by Thomas Moran, thomas.moran@mssm.edu) with a DAPI counterstain. Infected cells (488 nm) and total cells (DAPI) were quantified using the Celigo (Nexcelcom) imaging cytometer. Infectivity was measured by the accumulation of viral N protein (fluorescence accumulation). Percent infection was quantified as [(infected cells/total cells) – background] × 100, and the DMSO control was then set to 100% infection for analysis. Data were fit using nonlinear regression and IC_50_ values for each experiment were determined using GraphPad Prism version 8.0.0 (San Diego, CA). Cytotoxicity was also performed using the MTT assay (Roche), according to the manufacturer’s instructions. Cytotoxicity was performed in uninfected cells with same compound dilutions and concurrent with viral replication assay. All assays were performed in biologically independent triplicates.

### 2. Computational methods

#### 2.1 Synthetic route planning

We use an approach based on the Molecular Transformer technology (32). Our algorithm uses natural language processing to predict the outcomes of chemical reactions and design retrosynthetic routes starting from commercially available building blocks. This proprietary platform is provided free of charge by PostEra Inc (https://postera.ai/). Additionally, Manifold (https://app.postera.ai/manifold/) was built by PostEra Inc. during the project to search the entire space of purchasable molecules, and automatically find the optimal building blocks.

#### 2.2 Alchemical free-energy calculations

Large-scale alchemical free-energy calculations were conducted in batches (“Sprints”) in which each set of calculations aimed to prioritize compounds that could be produced from a common synthetic intermediate using Enamine’s extensive building block library, resulting in synthetic libraries of hundreds to tens of thousands. Virtual synthetic libraries were organized into a star map, where all transformations were made with respect to a single reference x-ray structure and compound with experimentally measured bioactivity. x-ray structures were prepared using the OpenEye Toolkit SpruceTK with manually controlled protonation states for the key His^41^:Cys^145^ catalytic dyad (variously using zwitterionic or uncharged states) and His^163^ in P1 (which interacts with the 3-aminopyridine or isoquinoline nitrogen in our primary lead series). As the most relevant protonation states were uncertain, when computational resources afforded, calculations were carried out using multiple protonation state variants (His^41^:Cys^145^ either neutral or zwitterionic; His^163^ neutral or protonated) and the most predictive model on available retrospective data for that scaffold selected for nominating prospective predictions for that batch. Initial poses of target compounds were generated via constrained conformer enumeration to identify minimally clashing poses using Omega (from the OpenEye Toolkit) using a strategy that closely follows an exercise described in a blog post by Pat Walters (https://practicalcheminformatics.blogspot.com/2020/03/building-on-fragments-from-diamondxchem_30.html). Alchemical free-energy calculations were then prepared using the open source perses relative alchemical free-energy toolkit (40) (https://github.com/choderalab/perses), and nonequilibrium switching alchemical free-energy calculations (81) were run on Folding@home using the OpenMM compute core (43). Nonequilibrium switching calculations used 1 ns nonequilibrium alchemical trajectories, where most calculations were performed with 1 fs time steps without constraints to hydrogen due to technical limitations that have been resolved in calculations using OpenMM 7.5.1 and later. We used the Open Force Field Initiative OpenFF “Parsley” small molecule force fields (39) (multiple generations between 1.1.1 and 1.3.1 were released and used as the project evolved) and the AMBER14SB protein force field (82) with recommended ion parameters (83, 84), and TIP3P water (85). As many assayed compounds as possible were included in each batch of transformations to enable continual retrospective assessment and to leverage existing measured affinities in extrapolating predicted affinities. Analysis of free-energy calculations used the maximum likelihood estimator (86) to reconstruct the optimal predicted absolute free energy (and hence pIC_50_) estimate from available experimental measurements. Calculations were analyzed using the fah-xchem dashboard (https://github.com/choderalab/fah-xchem) using the Bennett acceptance ratio (87, 88) (https://threeplusone.com/pubs/gecthesis) and posted online in real time for the medicinal chemistry team to consult in making decisions about which compounds to prioritize.

We note that our primary aim was computing estimates of relative binding free energies for large alchemical transformations using abundant computing resources [which exceeded 1 exaFLOP/s (38)] rather than aggressive optimization of the cost/transformation. Batches of transformations used between 100 and 200 parallel 4 ns nonequilibrium cycles per transformation, selected based on the number of atoms modified in the transformation, resulting in 100 to 200 ns per transformation in aggregate. A Tesla V100 achieves ~200 ns/day for our solvated Mpro complex, meaning ~2 to 4 GPU-days per transformation was consumed on a V100 equivalent GPU. To give typical scales, Fig. 2C, panel 1, ran 6319 transformations of 140 cycles, resulting in ~3.5 ms of simulation time or ~424K GPU-hours; Fig. 2C, panel 2, ran 5077 transformations of ~200 cycles, resulting in ~4 ms simulation time, or ~480K GPU-hours; Fig. 2C, panel 3, ran 686 transformations of ~200 cycles, resulting in ~548 μs of simulation time, or ~66K GPU-hours.

Scripts for setting up and analyzing the perses alchemical free-energy calculations on Folding@home, as well as an index of computed datasets and dashboards are available at https://github.com/foldingathome/covid-moonshot

Code used for generating the COVID Moonshot alchemical free-energy calculation web dashboards is available here: https://github.com/choderalab/fah-xchem

Retrospective calculations for transformations in the main synthetic series shown in Fig. 5A were performed with an early release of perses 0.10.2 constructed as a simplified example that anyone can run to illustrate how these calculations work on standard GPU workstations, and use standard alchemical replica exchange protocols of 5 ns per replica (which just take a few hours on standard workstations, as opposed to the expensive nonequilibrium protocols used in the Sprints). Input scripts for this calculation are available in the perses distribution under “examples/moonshot-mainseries/” (https://github.com/choderalab/perses/tree/main/examples/moonshot-mainseries).

#### 2.3 Structural flexibility and interactions analysis

Protein-ligand interactions are the driving forces for molecular recognition. In this work, the *PLIPify* repo (https://github.com/volkamerlab/plipify) is used to detect shared interaction hotspots within the different Mpro structures. *PLIPify* is a python wrapper built on top of PLIP (89), a tool that enables automatic generation of protein-ligand interaction profiles for single complexes, to allow combining these profiles for multiple structures.

To generate the hotspots (depicted in Fig. 3A), the fragalysis data were downloaded (as of July 2021, https://fragalysis.diamond.ac.uk/api/targets/?format=json&title=Mpro). The respective prealigned complex structures were further investigated (found under data/{target}/aligned/{crystal_name}/{crystal_name}_bound.pdb). Only one chain per structure is kept, and the structures are protonated using Amber’s *reduce* function. *PLIPify* is invoked, and structures are excluded from further analysis if they do not contain exactly one binding site (i.e., PLIP detects either zero or more than one binding sites), the sequence contains gaps (‘-’), or the sequence length differs more than a standard deviation from the average length across all investigated structures.

This procedure resulted in a final set of 367 complex structures, used to generate the interaction fingerprints. Note for this study, only hbond-donor, hbond-acceptor, salt bridge, hydrophobic, pi-stacking, and halogen interactions are inspected. Additional code was added to *PLIPify* to split the hbond-donor and hbond-acceptor interactions into backbone and sidechain interactions (https://github.com/volkamerlab/plipify/pull/18). Interacting residues are only included if the summed interaction count per residue over all investigated structures is greater than five. Careful examination of examples of the interactions led us to filter out the S144 interactions from the final report as none of the interactions were convincing (24 hbond-don-bb, 168 hbond-don-sc, and 4 hbond-acc-sc interactions). The resulting structural depiction (Fig. 3A) were generated using pymol, and structure Mpro-P1788_0A_bound_chainA (protonated) is displayed (scripts available at https://github.com/volkamerlab/plipify/blob/master/projects/01/fragalysis.ipynb). Finally, structures containing compounds exhibiting some of the major interactions identified were used to generate the figures in Fig. 3B.

### 4. Chemical methods

#### 4.1 HTC library synthesis

##### 4.1.1 Chan-Lam reaction

The arylamine library was made by reacting the boronic acid (fig. S7D), under the optimized reaction conditions (1 eq. amine; 0.2 eq. CuI; 0.8 eq. DMAP; 2 eq. Hex_3_N; DMSO; under air; RT; 2 days) with 296 amines (200 aromatic, 48 primary, and 48 secondary aliphatic amines; data S2). For library production, we used Echo LDV plates and an Echo *555* acoustic dispenser for liquid handling. After the allotted reaction time, plate copies were made after diluting the reaction mixture with 4.6 μl DMSO and transferring 1 μl of the obtained solution to a 384-well plate, for either biochemical assay or yield estimation.

##### 4.1.2 Amide coupling

The amide library was made by reacting the carboxylic acid (fig. S8E) under the optimized reaction conditions (2 eq. amine; 2 eq. EDC; 2 eq. HOAt; *5* eq. DIPEA; DMSO; RT; 24 hours) with 300 amines (202 aromatics, 49 primary, and 49 secondary aliphatic amines; data S2). For library production, we used Echo LDV plates and an Echo *555* acoustic dispenser for liquid handling. Plate copies were made after diluting the reaction mixture with 4 μl DMSO. For yield estimation, 1 μl of the diluted library was transferred to an LC/MS-ready 384-well plate, followed by dilution with 20% ACN in water to the final volume of 50 μl. The desired product was identified in 60% of wells.

#### 4.2 General compounds synthesis and characterization

All compounds were directly purchased from Enamine Inc., following Enamine’s standard quality control (QC) for compound collections. In addition, in the supplementary chemistry section of the supplementary materials, we discuss the synthesis procedure, as well as liquid chromatography–mass spectrometry (LC-MS) and 1H nuclear magnetic resonance (NMR) characterization of compounds which were discussed in the manuscript with associated bioactivity data.

All COVID Moonshot compounds are publicly available as a screening collection that can be ordered in bulk or as singleton through Enamine. The compound identifiers of the COVID Moonshot collection are in the supplementary data files, together with Enamine’s internal QC data comprising LC-MS spectra for all compounds and NMR spectra for selected compounds.

## Supplementary Material

Moonshot consortium members and funding list

Supplementary text

Supplementary data tables

## Data Availability

All compound designs, datasets, and x-ray structures can be browsed on the COVID Moonshot website (https://postera.ai/moonshot/). The compound submissions and experimental data are available via GitHub (https://github.com/postera-ai/COVID_moonshot_submissions), and the bioactivity data can be interactively browsed (https://covid.postera.ai/covid/activity_data). All data are also available in a permanent archive on Zenodo (91). Alchemical free-energy calculations code and datasets are indexed on GitHub (https://github.com/foldingathome/covid-moonshot) and are stored in a permanent archive on Zenodo (92). Analysis of alchemical free-energy calculations versus experimental potencies is stored at https://github.com/asapdiscovery/COVID_moonshot_FECs_data. All x-ray structures are available for interactive viewing and comparison or bulk download via Fragalysis (https://fragalysis.diamond.ac.uk/viewer/react/preview/target/Mpro). Structures were deposited to the PDB (data S4) and are also available in a permanent archive on Zenodo (93). With regard to synthesized compounds, we have made all compounds assayed here available from the current Enamine catalog and readily available for purchase from Enamine (and other suppliers) via the Manifold platform, accessible for each compound page on the COVID Moonshot website (https://covid.postera.ai/covid/activity_data).

## The COVID Moonshot Consortium

Hagit Achdout^12^, Anthony Aimon^13,14^, Dominic S. Alonzi^15^, Robert Arbon^16^, Jasmin C. Aschenbrenner^13,14^, Blake H. Balcomb^13,14^, Elad Bar-David^12^, Haim Barr^17^, Amir Ben-Shmuel^12^, James Bennett^18,19^, Vitaliy A. Bilenko^20,21^, Bruce Borden^23^, Pascale Boulet^24^, Gregory R. Bowman^25^, Lennart Brewitz^26^, Juliane Brun^15^, Sarma BVNBS^27^, Mark Calmiano^28^, Anna Carbery^13,29^ Daniel W. Carney^30^, Emma Cattermole^15^, Edcon Chang^30^, Eugene Chernyshenko^20^, Austin Clyde^31^, Joseph E. Coffland^32^, Galit Cohen^17^, Jason C. Cole^33^, Alessandro Contini^34^, Lisa Cox^35^, Tristan Ian Croll^36,37^, Milan Cvitkovic^38^, Steven De Jonghe^39^, Alex Dias^13,14^, Kim Donckers^39^, David L. Dotson^40^, Alice Douangamath^13,14^, Shirly Duberstein^17^, Tim Dudgeon^41^, Louise E. Dunnett^13,14^, Peter Eastman^42^, Noam Erez^12^, Charles J. Eyermann^43^, Michael Fairhead^18^, Gwen Fate^44^, Oleg Fedorov^18,19^, Rafaela S. Fernandes^45^, Lori Ferrins^43^, Richard Foster^47^, Holly Foster^47,48^, Laurent Fraisse^24^, Ronen Gabizon^46^, Adolfo García-Sastre^49,50,51,52,53^ Victor O. Gawriljuk^45^,^54^, Paul Gehrtz^46,35^, Carina Gileadi^18^ Charline Giroud^18,19^, William G. Glass^16,48^, Robert C. Glen^56^, Itai Glinert^12^, Andre S. Godoy^45^, Marian Gorichko^21^, Tyler Gorrie-Stone^13,14^, Ed J. Griffen^57^, Amna Haneef^58^, Storm Hassell Hart^59^, Jag Heer^60^, Michael Henry^16^, Michelle Hill^15,61^, Sam Horrell^13,14^, Qiu Yu Judy Huang^62^, Victor D. Huliak^20^ Matthew F. D. Hurley^63^, Tomer Israely^12^, Andrew Jajack^38^, Jitske Jansen^64^, Eric Jnoff^65^, Dirk Jochmans^39^, Tobias John^26,66^, Benjamin Kaminow^16,67^, Lulu Kang^68^, Anastassia L. Kantsadi^15,69^, Peter W. Kenny^70^, J. L. Kiappes^15,71^, Serhii O. Kinakh^20^ Boris Kovar^72^, Tobias Krojer^18,73^ Van Ngoc Thuy La^58^, Sophie Laghnimi-Hahn^24^, Bruce A. Lefker^44^, Haim Levy^12^, Ryan M. Lithgo^13,14^, Ivan G. Logvinenko^20^, Petra Lukacik^13,14^, Hannah Bruce Macdonald^16,74^, Elizabeth M. MacLean^18^, Laetitia L. Makower^15^, Tika R. Malla^18^, Peter G. Marples^13,14^, Tatiana Matviiuk^20^ Willam McCorkindale^75,74^, Briana L. McGovern^49,50^, Sharon Melamed^12^, Kostiantyn P. Melnykov^20,21^, Oleg Michurin^20^, Pascal Miesen^76^, Halina Mikolajek^13,14^, Bruce F. Milne^77,78^, David Minh^79^, Aaron Morris^38^, Garrett M. Morris^29^, Melody Jane Morwitzer^80^, Demetri Moustakas^81^, Charles E. Mowbray^24^, Aline M. Nakamura^45,82^, Jose Brandao Neto^13,14^, Johan Neyts^39^, Luong Nguyen^38^, Gabriela D. Noske^45^, Vladas Oleinikovas^28,83^, Glaucius Oliva^45^, Gijs J. Overheul^76^, C. David Owen^13,14^, Ruby Pai^38^, Jin Pan^38^, Nir Paran^12^, Alexander Matthew Payne^16,67^, Benjamin Perry^24,84^, Maneesh Pingle^27^, Jakir Pinjari^27,85^, Boaz Politi^12^, Ailsa Powell^13,14^, Vladimír Pšenák^72^, Iván Pulido^16^, Reut Puni^12^, Victor L. Rangel^86,87^, Rambabu N. Reddi^46^, Paul Rees^88^, St Patrick Reid^80^, Lauren Reid^57^, Efrat Resnick^46^, Emily Grace Ripka^38^, Ralph P. Robinson^44^, Jaime Rodriguez-Guerra^89^, Romel Rosales^49,50^, Dominic A. Rufa^16,67^, Kadi Saar^75^, Kumar Singh Saikatendu^30^, Eidarus Salah^26^, David Schaller^89^, Jenke Scheen^16^, Celia A. Schiffer^62^, Christopher J. Schofield^26^, Mikhail Shafeev^20^ Aarif Shaikh^27^, Ala M. Shaqra^62^, Jiye Shi^65,90^, Khriesto Shurrush^17^, Sukrit Singh^16^, Assa Sittner^12^, Peter Sjö^24^, Rachael Skyner^13,14^, Adam Smalley^28^, Bart Smeets^91^, Mihaela D. Smilova^18^, Leonardo J. Solmesky^17^, John Spencer^59^, Claire Strain-Damerell^13,14^, Vishwanath Swamy^27,92^, Hadas Tamir^12^, Jenny C. Taylor^93^, Rachael E. Tennant^94^, Warren Thompson^13,14^, Andrew Thompson^18,95^, Susana Tomásio^96^, Charles W. E. Tomlinson^13,14^, Igor S. Tsurupa^20^, Anthony Tumber^26^, Ioannis Vakonakis^15,97^, Ronald P. van Rij^76^, Laura Vangeel^39^, Finny S. Varghese^76,98^, Mariana Vaschetto^96^, Einat B. Vitner^12^, Vincent Voelz^63^, Andrea Volkamer^89,99^, Martin A. Walsh^13,14^, Walter Ward^101^, Charlie Weatherall^102^, Shay Weiss^12^, Kris M. White^49,50^, Conor Francis Wild^13,44^, Karolina D. Witt^103^, Matthew Wittmann^16^, Nathan Wright^18^, Yfat Yahalom-Ronen^12^, Nese Kurt Yilmaz^62^, Daniel Zaidmann^46^, Ivy Zhang^16^, Hadeer Zidane^17^, Nicole Zitzmann^15^, Sarah N. Zvornicanin^62^

^12^Israel Institute for Biological Research, Department of Infectious Diseases, Ness-Ziona, Israel. ^13^Diamond Light Source Ltd, Harwell Science and Innovation Campus, Didcot, OX11 0DE, UK. ^14^Research Complex at Harwell, Harwell Science and Innovation Campus, Didcot OX11 0FA, UK. ^15^University of Oxford, Department of Biochemistry, Oxford Glycobiology Institute, South Parks Road, Oxford OX1 3QU, UK. ^16^Sloan Kettering Institute, Memorial Sloan Kettering Cancer Center, Computational and Systems Biology Program, New York, NY 10065, USA. ^17^The Weizmann Institute of Science, Wohl Institute for Drug Discovery of the Nancy and Stephen Grand Israel National Center for Personalized Medicine, Rehovot, 7610001, Israel. ^18^University of Oxford, Nuffield Department of Medicine, Centre for Medicines Discovery, Oxford, OX3 7DQ, UK. ^19^University of Oxford, Nuffield Department of Medicine, Target Discovery Institute, Oxford, OX3 7FZ, UK. ^20^Enamine Ltd, Kyiv, 02094, Ukraine. ^21^Taras Shevchenko National University of Kyiv, Kyiv, 01601, Ukraine. ^22^Sloan Kettering Institute, Memorial Sloan Kettering Cancer Center, Pharmacology Graduate Program, New York, NY 10065, USA. ^23^Folding@Home Consortium. ^24^Drugs for Neglected Diseases Initiative (DNDi), Geneva, 1202, Switzerland. ^25^University of Pennsylvania, Departments of Biochemistry and Biophysics and Bioengineering, Philadelphia, PA 19083, USA. ^26^University of Oxford, Department of Chemistry, Chemistry Research Laboratory, Oxford, OX1 3TA, UK. ^27^Sai Life Sciences Limited, ICICI Knowledge Park, Shameerpet, Hyderabad 500 078, Telangana, India. ^28^UCB, Slough, SL13WE, UK. ^29^University of Oxford, Department of Statistics, Oxford OX1 3LB, UK. ^30^Takeda Development Center Americas, Inc., San Diego, CA 92121, USA. ^31^Argonne National Lab, Lemont, IL 60439, USA. ^32^Cauldron Development Oy, Helsinki, 00140, Finland. ^33^Cambridge Crystallographic Data Centre, Cambridge, CB2 1EZ, UK. ^34^University of Milan, Department of General and Organic Chemistry, Milan, 20133, Italy. ^35^Life Compass Consulting Ltd, Macclesfield, SK10 5UE, UK. ^36^The University of Cambridge, Cambridge Institute for Medical Research, Department of Haematology, Cambridge CB2 0XY, UK. ^37^Present address: Altos Labs, BioML group, Great Abington, CB216GP. ^38^PostEra Inc., Cambridge, MA, 02142, USA. ^39^KU Leuven, Department of Microbiology, Immunology and Transplantation, Rega Institute for Medical Research, Laboratory of Virology and Chemotherapy, Leuven, Belgium. ^40^Datryllic LLC, Phoenix AZ, 85003, USA. ^41^Informatics Matters Ltd, Bicester, OX26 6JU, UK. ^42^Stanford University, Department of Chemistry, Stanford, CA 94305, USA. ^43^Northeastern University, Department of Chemistry and Chemical Biology, Boston MA 02115, USA. ^44^Thames Pharma Partners LLC, Mystic, CT 06355, USA. ^45^University of Sao Paulo, Sao Carlos Institute of Physics, Sao Carlos, 13563-120, Brazil. ^46^The Weizmann Institute of Science, Department of Chemical and Structural Biology, Rehovot, 7610001, Israel. ^47^University of Leeds, School of Chemistry, Leeds, LS2 9JT, UK. ^48^Present address: Exscientia, Oxford Science Park, Oxford, OX4 4GE, UK. ^49^Icahn School of Medicine at Mount Sinai, Department of Microbiology, New York, NY 10029, USA. ^50^Icahn School of Medicine at Mount Sinai, Global Health and Emerging Pathogens Institute, New York, NY 10029, USA. ^51^Icahn School of Medicine at Mount Sinai, Department of Medicine, Division of Infectious Diseases, New York, NY 10029, USA. ^52^Icahn School of Medicine at Mount Sinai, The Tisch Cancer Institute, New York, NY 10029, USA. ^53^Icahn School of Medicine at Mount Sinai, Department of Pathology, Molecular and Cell-Based Medicine, New York, NY 10029, USA. ^54^Present address: University of Groningen, Groningen Research Institute of Pharmacy, Department of Drug Design, Groningen, 9700 AV, Netherlands. ^55^Present address: Merck Healthcare KGaA, Darmstadt, 64293, Germany. ^56^University of Cambridge, Department of Chemistry, Cambridge, CB2 1EW, UK. ^57^MedChemica Ltd, Macclesfield, Cheshire. SK11 6PU UK. ^58^Illinois Institute of Technology, Department of Biology, Chicago IL 60616 USA. ^59^University of Sussex, Department of Chemistry, School of Life Sciences, Brighton, East Sussex, BN1 9QJ, UK. ^60^Syngene International Limited, Headington, Oxford, OX3 7BZ, UK. ^61^Present address: Sir William Dunn School of Pathology, Oxford. OX13RE, UK. ^62^University of Massachusetts, Chan Medical School, Department of Biochemistry and Molecular Biotechnology, Worcester MA 01655, USA. ^63^Temple University, Department of Chemistry, Philadelphia, PA 19122, USA. ^64^RWTH Aachen University, Institute of Experimental Medicine and Systems Biology, Aachen, 52074, Germany. ^65^UCB, Chemin du Foriest, 1420 Braine-l’Alleud, Belgium. ^66^Present address: AMSilk, Neuried, 82061, Germany. ^67^Sloan Kettering Institute, Memorial Sloan Kettering Cancer Center, Tri-Institutional Program in Computational Biology and Medicine, New York, NY 10065, USA. ^68^Illinois Institute of Technology, Department of Applied Mathematics, Chicago IL 60616 USA. ^69^University of Thessaly, Department of Biochemistry and Biotechnology, Larissa, 415 00, Greece. ^70^Berwick-on-Sea, North Coast Road, Blanchisseuse, Saint George, Trinidad and Tobago. ^71^Present address: University College of London, Department of Chemistry, London WC1H 0AJ, UK. ^72^M2M solutions s.r.o. Žilina, 010 01, Slovakia. ^73^MAX IV Laboratory, Fotongatan 2, 224 84 Lund, Sweden. ^74^Present address: Charm Therapeutics, London, N1C 4AG, UK. ^75^University of Cambridge, Cavendish Laboratory, Cambridge, CB3 0HE UK. ^76^Radboud University Medical Center, Department of Medical Microbiology, Radboud Institute for Molecular Life Sciences, Nijmegen, 6525 GA, Netherlands. ^77^University of Aberdeen, Department of Chemistry, Old Aberdeen, AB24 3UE Scotland, UK. ^78^University of Coimbra, CFisUC, Department of Physics, Coimbra, 3004-516, Portugal. ^79^Illinois Institute of Technology, Department of Chemistry, Chicago IL 60616 USA. ^80^University of Nebraska Medical Centre, Dept of Pathology and Microbiology, Omaha, NE 68198-5900, USA. ^81^Relay Therapeutics, Cambridge, MA 02139, USA. ^82^Present address: Instituto Butantan, Sao Paulo, 05503-900, Brazil. ^83^Present address: Monte Rosa Therapeutics, Basel, CH 4057, Switzerland. ^84^Present address: Medicxi, Geneva, 1204, Switzerland. ^85^Present address: Sun Pharma Advanced Research Company (SPARC), Baroda, India. ^86^University of São Paulo, Ribeirão Preto School of Pharmaceutical Sciences, Ribeirao Preto - SP/CEP 14040-903, Brazil. ^87^Present address: Evotec (UK) Ltd, Milton Park, Abingdon, Oxfordshire, OX14 4RZ, UK. ^88^Compass Bussiness Partners Ltd, Southcliffe, Bucks, SL9 0PD, UK. ^89^Charité – Universitätsmedizin Berlin, In silico Toxicology and Structural Bioinformatics, Berlin, 10117, Germany. ^90^Present address: Eli Lilly and Company, San Diego, CA 92121, USA. ^91^Radboud University Medical Center, Department of pathology, Radboud Institute for Molecular Life Sciences, Nijmegen, 6525 GA, Netherlands. ^92^Present address: TCG Life Sciences, Pune, India. ^93^Uni versity of Oxford, Nuffield Department of Medicine, Wellcome Centre for Human Genetics, Oxford OX3 7BN, UK. ^94^Lhasa Limited, Leeds, LS11 5PS, UK. ^95^Present address: Walter and Eliza Hall Institute, Parkville 3052, Victoria, Australia. ^96^Collaborative Drug Discovery, Cambridge, CB2 1GE, UK. ^97^Present address: Lonza Biologics, Lonza Ltd, Lonzastrasse, CH-3930 Visp, Switzerland. ^98^Present address: uniQure Biopharma, Amsterdam, 1105 BP, Netherlands. ^99^Present address: Saarland University, Data Driven Drug Design, Campus - E2.1, 66123 Saarbrücken, Germany. ^100^University of Johannesburg, Department of Biochemistry, Auckland Park, 2006, South Africa. ^101^Walter Ward Consultancy and Training, Derbyshire, SK22 4AA, UK. ^102^Collaborative Drug Discovery, Burlingame, CA 94010, USA. ^103^University of Oxford, Nuffield Department of Medicine, Pandemic Sciences Institute, Oxford, Oxon, OX3 7DQ, UK.
