# Supplementary material for: Open Science Discovery of Potent Non-Covalent SARS-CoV-2 Main Protease Inhibitors
Source: Science. Author manuscript; Available in PMC 2024 Apr 17. (PMC7615835; doi:10.1126/science.abo7201)
Supplement: Supplementary material [file EMS194244-supplement-Supplementary_material.pdf]

Supplementary Chemistry

Abbreviations Used
EDC, 1-ethyl-3-(3-dimethylaminopropyl)carbodiimide;
TEA, triethylamine;
CDI, 1,1′-carbonyldiimidazole;
DMSO, dimethyl sulfoxide;
DIPEA, N-ethyl-N-isopropylpropan-2-amine;
DCM, dichloromethane;
HOAc, acetic acid;
MeOH, methanol;
DMSO, dimethyl sulfoxide;
HPLC, high-performance liquid chromatography;
LCMS, liquid chromatography-mass spectrometry;
min, minute(s);
h, hour(s);
HOAt, 1-hydroxy-7-azabenzotriazole;
HATU, *N*-[(dimethylamino)-1*H*-1,2,3-triazolo-[4,5-*b*]pyridin-1-ylmethylene]-*N*-methylmethanaminium hexafluorophosphate *N*-oxide;
IPA, isopropanol;
RT, retention time;
equiv., equivalent;
TFA, trifluoroacetic acid;

General procedure

2-(3-Chlorophenyl)-N-(isoquinolin-4-yl)acetamide (Z1530724813)

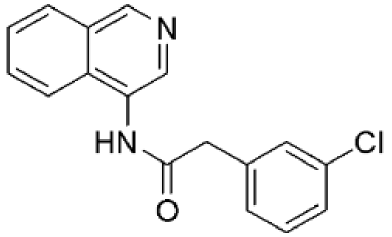

Isoquinolin-4-amine hydrochloride (61.6 mg, 342.14 mmol), 2-(3-chlorophenyl)acetic acid (64.0 mg, 376.45 mmol), EDC (64.1 mg, 413.18 mmol), TEA (41.4 mg, 409.41 mmol), and HOAt (50.82 mmol, 410.12 mmol) were mixed in anhydrous DMSO (0.5 mL). The reaction mixture was sealed and kept at 25 °C for 18 h. After that, the mixture was evaporated under reduced pressure, and the residue was dissolved in DMSO (0.6 mL). The solution was filtered, analyzed by LCMS, and then purified by HPLC to afford 2-(3-chlorophenyl)-N-(isoquinolin-4-yl)acetamide (32.0 mg, 31.62%) as a yellow solid.

^{1}H NMR (500 MHz, DMSO-*d*$_6$) δ (ppm) 10.26 (s, 1H), 9.14 (s, 1H), 8.68 (s, 1H), 8.14 (d, *J* = 8.1 Hz, 1H), 8.05 (d, *J* = 8.5 Hz, 1H), 7.82 (dd, *J* = 8.4, 6.9 Hz, 1H), 7.70 (t, *J* = 7.5, 7.5 Hz, 1H), 7.48 (s, 1H), 7.42-7.36 (m, 2H), 7.36-7.30 (m, 1H), 3.87 (s, 2H).

MS (ESI+) m/z calculated for $C_{17}H_{14}ClN_2O^+$ ([M+H]$^+$) 297.1, found: 297.0.

HPLC condition:
Column: Chromatorex 18 SMB100-5T 100x19 mm)
Mobile phase: 40:40:90% at 0:1:5 min, H_2O/MeOH
Flow rate: 30 mL/min

2-(3-Chlorophenyl)-N-(4-methylpyridin-3-yl)acetamide (Z1129289650)

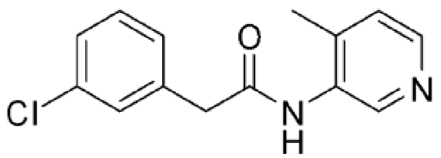

4-Methylpyridin-3-amine (40.9 mg, 378.46 mmol), 2-(3-chlorophenyl)acetic acid (71.0 mg, 417.62 mmol), EDC (71.1 mg, 458.30 mmol), and HOAt (53.69 mg, 438.50 mmol) were mixed in anhydrous DMSO (0.5 mL). The reaction mixture was sealed and kept at 25 °C for 18 h. After that, the mixture was evaporated under reduced pressure; and the residue was dissolved in DMSO (0.7 mL). The solution was filtered, analyzed by LCMS, and then purified by HPLC (eluting: 40-40-90% 0-1-5 min H_2O/MeOH, flow: 30 mL/min (loading pump 4 mL/min methanol); column: Chromatorex 18 SMB100-5T 100x19 mm) to afford 2-(3-chlorophenyl)-N-(4-methylpyridin-3-yl)acetamide (62.6 mg, 63.46%) as a yellow solid.
^{1}H NMR (500 MHz, DMSO-*d$_6$*) δ (ppm) 9.76 (s, 1H), 8.47 (s, 1H), 8.22 (d, *J* = 5.0 Hz, 1H), 7.44-7.39 (m, 1H), 7.39-7.34 (m, 1H), 7.34-7.28 (m, 2H), 7.23 (d, *J* = 4.9 Hz, 1H), 3.72 (s, 2H), 2.16 (s, 3H).
MS (ESI+) m/z calculated for $C_{14}H_{14}ClN_2O^+$ ($[M+H]^+$) 261.1, found: 261.0.

(R)-6-Chloro-N-(isoquinolin-4-yl)chromane-4-carboxamide (Z4643752419) and (S)-6-Chloro-N-(isoquinolin-4-yl)chromane-4-carboxamide (Z4646694589)

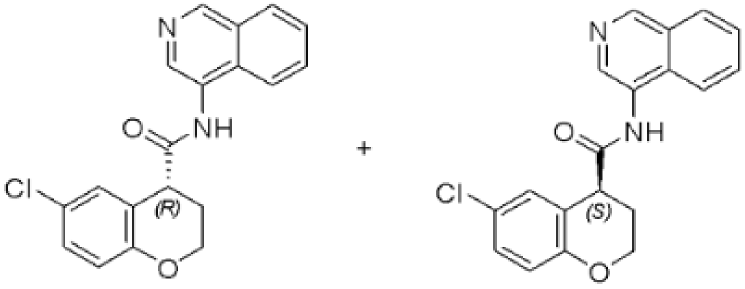

6-chlorochromane-4-carboxylic acid (0.156 g, 735.84 µmol, 1 eq), isoquinolin-4-amine (0.127 g, 937.72 µmol, 1.2 eq), HATU (377.5 mg, 0.99 mmol, 1.35 eq), and DIPEA (284.77 mg, 2.20 mmol, 3 eq) were mixed in anhydrous DMSO (3 mL), and the solution was stirred at 20 °C for 12 h . After that, the reaction mixture was purified by reverse phase HPLC to afford (R,S)-6-chloro-N-(isoquinolin-4-yl)chromane-4-carboxamide (90 mg, 36%).

(R)-6-Chloro-N-(isoquinolin-4-yl)chromane-4-carboxamide (Z4643752419) and (S)-6-Chloro-N-(isoquinolin-4-yl)chromane-4-carboxamide (Z4646694589) were obtained as two fractions (RT = 32.069 min and 46.990 min respectively) from separation of the (R,S)-6-chloro-N-(isoquinolin-4-yl)chromane-4-carboxamide (90 mg) using a Chiralpak IC-III (250*20 mm, 5 µm) column, eluting with hexane:IPA:MeOH (80-10-10) with flow rate of 13 mL/min.
^{1}H NMR (500 MHz, DMSO-*d$_6$*) δ (ppm) 10.38 (s, 1H), 9.17 (s, 1H), 8.69 (s, 1H), 8.16 (d, *J* = 8.2 Hz, 1H), 8.09 (d, *J* = 8.5 Hz, 1H), 7.85 (dd, *J* = 8.4, 7.0 Hz, 1H), 7.72 (t, *J* = 7.5, 7.5 Hz, 1H), 7.37 (d, *J* = 2.6 Hz, 1H), 7.19 (dd, *J* = 8.8, 2.7 Hz, 1H), 6.85 (d, *J* = 8.8 Hz, 1H), 4.43-4.35 (m, 1H), 4.26-4.18 (m, 1H), 4.17 (t, *J* = 5.7, 5.7 Hz, 1H), 2.30-2.20 (m, 2H).
MS (ESI+) m/z calculated for $C_{19}H_{16}ClN_2O_2^+$ ($[M+H]^+$) 339.1, found: 339.0.

(R,S)-6-chloro-N-(isoquinolin-4-yl)-1,2,3,4-tetrahydroisoquinoline-4-carboxamide

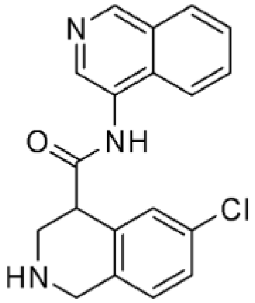

Step1. 4-bromo-6-chloroisoquinoline (6.9 g, 28.64 mmol), triethylamine (2.88 g, 28.47 mmol) and Pd(dppf)Cl_2.2CH_2Cl_2 (1.16 g, 1.43 mmol) were dissolved in MeOH (100 mL). The reaction mixture was stirred at 80 °C under CO atmosphere (25 bar) for 16 h. After cooling, the reaction mixture was filtered, and the combined MeOH solution was evaporated under reduced pressure to give a residue. The residue was then suspended in water, and the suspension was filtered, air-dried to afford methyl 6-chloroisoquinoline-4-carboxylate (5.0 g, 79.3%). which was used in the subsequent step without further purification.

Step 2. Methyl 6-chloroisoquinoline-4-carboxylate (5.0 g, 22.62 mmol) was dissolved in acetic acid (50 mL). Sodium cyanoborohydride (4.25 g, 67.48 mmol) was then added portionwise to the solution at room temperature, and the reaction mixture was stirred overnight. After that, the reaction mixture was evaporated, and the residue was diluted with water then extracted with $CHCl_3$ (2 x 300 mL). The combined organic layer was dried over sodium sulfate and evaporated *in vacuo* to give methyl 6-chloro-1,2,3,4-tetrahydroisoquinoline-4-carboxylate (3.4 g, 66.8%) which was used in subsequent step without further purification.

Step 3. di-*tert*-butyl dicarbonate (3.14 g, 14.4 mmol, 1.2 equiv.) was added to the solution of methyl 6-chloro-1,2,3,4-tetrahydroisoquinoline-4-carboxylate (3.4 g, 12.0 mmol) and $NaHCO_3$ (5 equiv., aqueous solution) in MeOH (100 mL). The reaction mixture was stirred at 50 °C overnight. Then, the mixture was diluted with water and extracted with chloroform (2 x 50 mL). The combined organic layer was dried over sodium sulfate and evaporated to give 2-tert-butyl 4-methyl 6-chloro-3,4-dihydroisoquinoline-2,4(1H)-dicarboxylate (3.4g), which was used in the subsequent step without further purification.

Step 4. 2-tert-butyl 4-methyl 6-chloro-3,4-dihydroisoquinoline-2,4(1H)-dicarboxylate (3.4 g, 10.4 mmol) was mixed in MeOH (30 mL) and H_2O (60 mL), and then NaOH (1.24 g, 31 mmol) was added to the solution. The reaction mixture was heated at 50 °C overnight. Then, the mixture was diluted with water, acidified with $NaHSO_4$ to pH 4 and extracted with chloroform (2 x 50 mL). The combined organic layer was dried over sodium sulfate and evaporated to give 2-(tert-butoxycarbonyl)-6-chloro-1,2,3,4-tetrahydroisoquinoline-4-carboxylic acid (2.9 g), which was used in the subsequent step without further purification.

Step 5. 2-(tert-butoxycarbonyl)-6-chloro-1,2,3,4-tetrahydroisoquinoline-4-carboxylic acid (4.0 g, 12.86 mmol), isoquinolin-4-amine hydrochloride (2.21 g, 12.26 mmol), (3-[(ethylimino)methylidene]aminopropyl)dimethylamine hydrochloride (3.51 g, 18.39 mmol), N,N-dimethylpyridin-4-amine (298.58 mg, 2.45 mmol), and triethylamine (1.85 g, 18.34 mmol, 2.55 mL) were suspended in DMF, and the reaction mixture was heated at 50 °C overnight. After cooling to room temperature, the mixture was evaporated to give a residue, which was then suspended in water and filtered to give tert-butyl 6-chloro-4-(isoquinolin-4-ylcarbamoyl)-3,4-dihydroisoquinoline-2(1H)-carboxylate (1.45 g, 35.1%), which was used in the subsequent step without further purification.

Step 6. tert-butyl 6-chloro-4-(isoquinolin-4-ylcarbamoyl)-3,4-dihydroisoquinoline-2(1H)-carboxylate (1.39 g, 3.18 mmol) was dissolved in MeOH, and then acetyl chloride (748.0 mg, 9.59 mmol, 680.0 µL) was added at to the solution at room temperature. The reaction mixture was heated at 50 °C overnight. Then, the mixture was diluted with water, basified with potassium carbonate to pH 12, and extracted with $CHCl_3$. The organic layer was dried over sodium sulfate. The volatiles were removed under reduced pressure to give (R,S)-6-chloro-N-(isoquinolin-4-yl)-1,2,3,4-tetrahydroisoquinoline-4-carboxamide (0.9 g, 84%).
^{1}H NMR (500 MHz, DMSO-*d*$_6$) δ (ppm) 11.25 (s, 1H), 9.09 (s, 1H), 8.92 (s, 1H), 8.14 (d, *J* = 8.2 Hz, 1H), 8.00 (d, *J* = 8.5 Hz, 1H), 7.86-7.79 (m, 1H), 7.71 (t, *J* = 7.5, 7.5 Hz, 1H), 7.37 (d, *J* = 2.2 Hz, 1H), 7.26 (dd, *J* = 8.2, 2.3 Hz, 1H), 7.18 (d, *J* = 8.3 Hz, 1H), 4.04 (d, *J* = 16.3 Hz, 1H), 3.92 (d, *J* = 16.4 Hz, 1H), 3.83 (t, J = 4.0, 4.0 Hz, 1H), 3.46 (dd, J = 12.7, 3.5 Hz, 1H), 3.13 (dd, *J* = 12.8, 4.4 Hz, 1H).
MS (ESI+) m/z calculated for $C_{19}H_{17}ClN_3O^+$($[M+H]^+$) 338.1, found: 338.2.

rel-(R)-6-chloro-N-(isoquinolin-4-yl)-1,2,3,4-tetrahydroisoquinoline-4-carboxamide (Z4943052515, rel-BEN-DND-f2e727cd-5-1) and rel-(R)-6-chloro-N-(isoquinolin-4-yl)-1,2,3,4-tetrahydroisoquinoline-4-carboxamide (Z4943052518, rel-BEN-DND-f2e727cd-5-2)

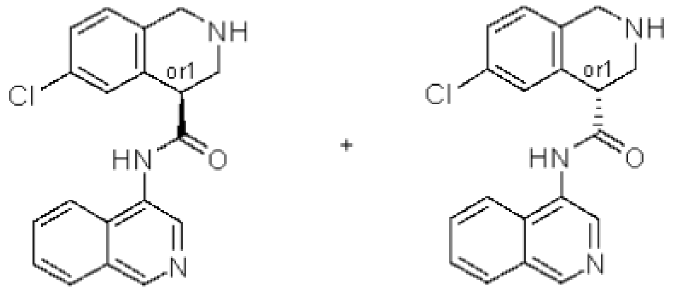

(R,S)-6-chloro-N-(isoquinolin-4-yl)-1,2,3,4-tetrahydroisoquinoline-4-carboxamide (50 mg) was separated using the condition mentioned below to afford two isomers (**Z4943052515, Z4943052518**) at RT = 21.465 min and RT = 31.200 min.
Chiral separation condition:
Column: Chiralcel OD-H (250*20 mm, 5 µm)
Mobile phase: hexane:IPA:MeOH, 80:10:10
Flow rate: 12 mL/min
Z4943052515, rel-BEN-DND-f2e727cd-5-1:
Yield: 18.02 mg (36.04%)
Analytical RT = 11.878 min (Chiralcel OD-H (250*4.6 mm, 5 µm), hexane:IPA:MeOH, 70:15:15, 0.6 mL/min) .
Z4943052518, rel-BEN-DND-f2e727cd-5-2:
Yield: 17.94 mg (35.88%)
Analytical RT = 15.304 min (Chiralcel OD-H (250*4.6 mm, 5 µm), hexane:IPA:MeOH, 70:15:15, 0.6 mL/min).

(R,S)-6-Chloro-N-(isoquinolin-4-yl)-2-(methylsulfonyl)-1,2,3,4-tetrahydroisoquinoline-4-carboxamide

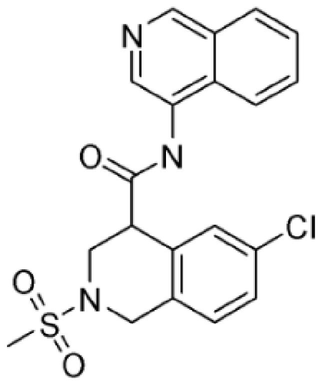

6-chloro-N-(isoquinolin-4-yl)-1,2,3,4-tetrahydroisoquinoline-4-carboxamide (100 mg, 0.3 mmol) and DIPEA (0.1 mL) were dissolved in DMF (4 mL), and then methanesulfonyl chloride (40 mg, 0.36 mmol) was added to the solution. The reaction mixture was stirred at room temperature for 16 h. Then, the mixture was purified by HPLC (25-50% 0-5 min H_2O/ACN, flow: 30 mL/min (loading pump 4 mL/min acetonitrile); column: Chromatorex 18 SMB100-5T 100x19 mm 5 µm) to give (R,S)-6-chloro-N-(isoquinolin-4-yl)-2-(methylsulfonyl)-1,2,3,4-tetrahydroisoquinoline-4-carboxamide (34 mg).
^{1}**H NMR (500 MHz, DMSO-*d*$_6$)** δ (ppm) 10.37 (s, 1H), 9.18 (s, 1H), 8.67 (s, 1H), 8.16 (dd, *J* = 8.3, 4.9 Hz, 2H), 7.87-7.80 (m, 1H), 7.72 (t, *J* = 7.5, 7.5 Hz, 1H), 7.42 (d, *J* = 2.1 Hz, 1H), 7.35 (dd, *J* = 8.3, 2.2 Hz, 1H), 7.31 (d, *J* = 8.3 Hz, 1H), 4.43 (s, 2H), 4.36 (t, *J* = 5.9, 5.9 Hz, 1H), 3.83 (d, J = 5.8 Hz, 2H), 3.00 (s, 3H).
MS (ESI+) m/z calculated for $C_{20}H_{19}ClN_3O_3S^+$([M+H]$^+$) 416.1, found: 416.0.

rel-(R)-6-chloro-N-(isoquinolin-4-yl)-2-(methylsulfonyl)-1,2,3,4-tetrahydroisoquinoline-4-carboxamide (Z4988872945) and rel-(R)-6-chloro-N-(isoquinolin-4-yl)-2-(methylsulfonyl)-1,2,3,4-tetrahydroisoquinoline-4-carboxamide (Z4988873021)

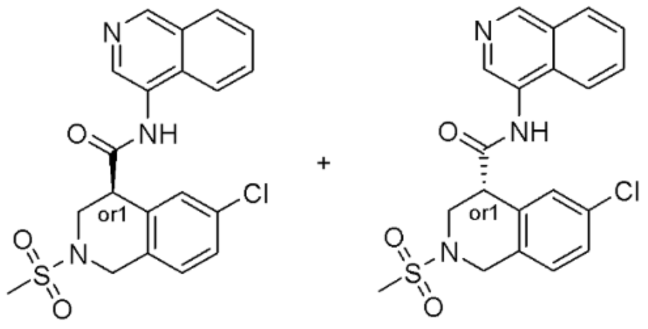

(R,S)-6-Chloro-N-(isoquinolin-4-yl)-2-(methylsulfonyl)-1,2,3,4-tetrahydroisoquinoline-4-carboxamide (50 mg) was separated using the condition mentioned below to afford two isomers (***Z4988872945 and Z4988873021***) at RT = 41.218 min and RT = 51.863 min.

Chiral separation condition:

Column: Chiralpak IG (250*20 mm, 5 µm)
Mobile phase: hexane:IPA:MeOH, 50:25:25
Flow rate: 12 mL/min

Z4988872945:
Yield: 10.49 mg (42.30%)
Analytical RT = 20.869 min (Chiralpak IG (250*4.6 mm, 5 µm), IPA:MeOH, 50:50, 0.6 mL/min).
Z4988873021:
Yield: 10.40 mg (41.94%)
Analytical RT = 16.342 min (Chiralpak IG (250*4.6 mm, 5 µm), IPA:MeOH, 50:50, 0.6 mL/min).

(R,S)-6-chloro-2-(((1-cyanocyclopropyl)methyl)sulfonyl)-N-(isoquinolin-4-yl)-1,2,3,4-tetrahydroisoquinoline-4-carboxamide

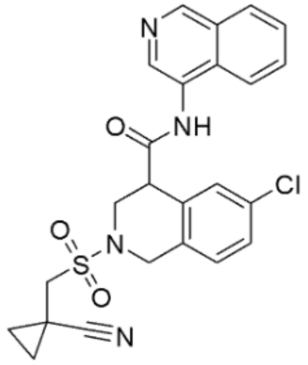

Step 1. tert-butyl 6-chloro-4-(isoquinolin-4-ylcarbamoyl)-3,4-dihydroisoquinoline-2(1H)-carboxylate (2.2 g, 97% purity) was dissolved in dioxane, and HCl (8 % solution in dioxane, 3 equiv.) was added to the solution. The reaction mixture was stirred at room temperature overnight. The crystalline substance was filtered off, washed with acetone, and dried to afford crude 6-chloro-N-(isoquinolin-4-yl)-1,2,3,4-tetrahydroisoquinoline-4-carboxamide dihydrochloride, which was used in the next step without further purification (1.8 g, 98%).

Step 2. 6-chloro-N-(isoquinolin-4-yl)-1,2,3,4-tetrahydroisoquinoline-4-carboxamide dihydrochloride (600.0 mg, 1.47 mmol) and triethylamine (739.52 mg, 7.31 mmol, 1.02 mL) were dissolved in DCM and cooled to 5-10 °C. (1-cyanocyclopropyl)methanesulfonyl chloride (393.82 mg, 2.2 mmol) was then added to the reaction, and the reaction mixture was stirred at room temperature for 48 h. The reaction mixture was evaporated, and the crude product was purified by preparative HPLC to afford (R,S)-6-chloro-2-(((1-cyanocyclopropyl)methyl)sulfonyl)-N-(isoquinolin-4-yl)-1,2,3,4-tetrahydroisoquinoline-4-carboxamide (142.0 mg, 20.2%).
^{1}H NMR (500 MHz, DMSO-d_6) δ (ppm) 10.43 (s, 1H), 9.18 (s, 1H), 8.68 (s, 1H), 8.16 (t, *J* = 8.5, 8.5 Hz, 2H), 7.84 (t, *J* = 7.7, 7.7 Hz, 1H), 7.72 (t, *J* = 7.5, 7.5 Hz, 1H), 7.43 (d, *J* = 2.1 Hz, 1H), 7.39-7.33 (m, 1H), 7.31 (d, *J* = 8.4 Hz, 1H), 4.57-4.46 (m, 2H), 4.36 (t, *J* = 6.0, 6.0 Hz, 1H), 3.97-3.84 (m, 2H), 3.61-3.50 (m, 2H), 1.43-1.38 (m, 2H), 1.24-1.19 (m, 2H).
MS (ESI+) m/z calculated for $C_{24}H_{22}ClN_4O_3S^+$([M+H]$^+$) 481.1, found: 481.2.

(4S)-6-chloro-2-[(1-cyanocyclopropyl)methylsulfonyl]-N-(4-isoquinolyl)-3,4-dihydro-1H-isoquinoline-4-carboxamide (Z5129808241, rel-MAT-POS-dc2604c4-1-2) and (4R)-6-chloro-2-[(1-cyanocyclopropyl)methylsulfonyl]-N-(4-isoquinolyl)-3,4-dihydro-1H-isoquinoline-4-carboxamide (Z5129808244, re-MAT-POS-dc2604c4-1-1)

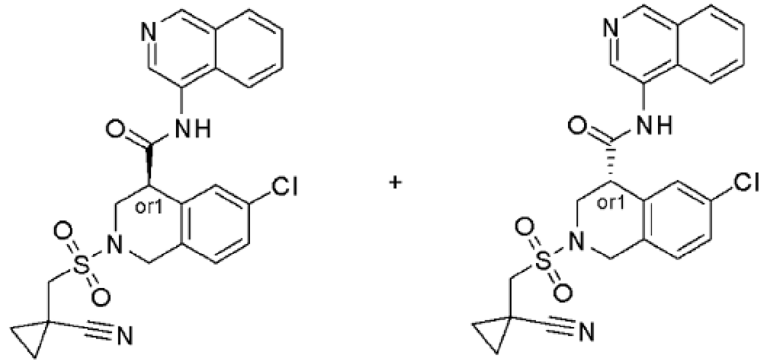

(R,S)-6-chloro-2-(((1-cyanocyclopropyl)methyl)sulfonyl)-N-(isoquinolin-4-yl)-1,2,3,4-tetrahydroisoquinoline-4-carboxamide (90 mg) was separated using the condition mentioned below to afford (***Z4988872945 and Z4988873021***) at RT = 15.399 min and 23.429 min.

Chiral separation condition:

Column: CHIRALPAK IC (250x21 mm, 5 µm)
Mobile phase: IPA:MeOH, 50:50
Flow rate: 12 mL/min

Z5129808241, rel-MAT-POS-dc2604c4-1-2:

Yield: 480.37 mg (48.04%)

Analytical RT = 12.281 min (Chiralpak IA (250x4.6 mm, 5 µm); MeOH:IPA, 50:50; flow rate: 0.6 mL/min).

Z5129808244, rel-MAT-POS-dc2604c4-1-1:

Yield: 436.07 mg (43.61%)

Analytical RT = 20.221 min (Chiralpak IA (250x4.6 mm, 5 µm); MeOH:IPA, 50:50; flow rate: 0.6 mL/min).

(2-hydroxyquinolin-4-yl)(4-(3-(trifluoromethyl)phenyl)piperazin-1-yl)methanone

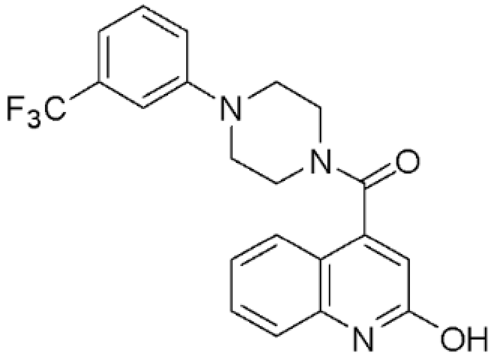

ERI-UCB-a0b0dbcb-4

A vial was charged with 2-hydroxyquinoline-4-carboxylic acid (typically 0.1 mmol, 1.0 equiv.), and CDI (prepared in advance at 15% solution in DMSO, 1.1 equiv.) was then added to the vial. The reaction mixture was heated with stirring at 50 °C for 2 h. After that, 1-(3-(trifluoromethyl)phenyl)piperazine (1.1 equiv.) was added to the reaction, and the vial was sealed and heated at 100 °C for 6 h. In the case of using a salt of the amine, an additional amount of DIPEA was added to the reaction mixture to convert the amine to the basic form. After cooling to the ambient temperature, the mixture was filtered and the solution was subjected to HPLC purification.

^{1}H NMR (600 MHz, DMSO-*d_6*) δ (ppm) 11.92 (s, 1H), 7.53 (m, 1H), 7.42 (m, 1H), 7.34 (m, 1H), 7.19 (m, 3H), 7.07 (d, *J* = 5.0 Hz, 1H), 6.51 (s, 1H), 3.80 (m, 2H), 3.32 (m, 4H), 3.20 (m, 1H), 3.01 (m, 1H).

MS (ESI+) m/z calculated for $C_{21}H_{19}F_3N_3O_2^+$ ([M+H]$^+$) 402.1, found: 402.4.

Separation condition:

Column: Waters Sunfire C18 OBD Prep Column, 100 Å^2, 5 µm, 100x19 mm with SunFire C18 Prep Guard Cartridge

Mobile phase: H_2O:MeOH. In some cases, ammonia or TFA was used as an additive to improve the separation of the products.

6-chloro-*N*-(isoquinolin-4-yl)thiochromane-4-carboxamide 1,1-dioxide

Step 1. To the solution of 6-chloro-3,4-dihydro-2H-1-benzothiopyran-4-one (2.0 g, 10.07 mmol) in DCM (20 mL) were added trimethylsilanecarbonitrile (1.3 g, 13.09 mmol) and a catalytic amount of diiodozinc (160.69 mg, 503.4 µmol). The reaction mixture was stirred at room temperature overnight, at which time, a check by NMR showed full conversion. The volatiles were removed in vacuo to give a crude 6-chloro-4-[(trimethylsilyl)oxy]-3,4-dihydro-2H-1-benzothiopyran-4-carbonitrile (2.99 g, 99.7%), which was used in the next step without further purification.

Step 2. To a 50-mL single neck round bottom reaction flask equipped with a magnetic stirrer, a reflux condenser, and a nitrogen inlet were added 6-chloro-4-[(trimethylsilyl)oxy]-3,4-dihydro-2H-1-benzothiopyran-4-carbonitrile (2.99 g, 10.04 mmol), tin(II) chloride dihydrate (9.14 g, 40.16 mmol), glacial acetic acid (8 mL), and concentrated hydrochloric acid (8 mL). The reaction apparatus was immediately flushed with Argon and plunged into a preheated (100 °C) oil bath. With vigorous stirring, the reaction mixture was heated for 48 h. After cooling to room temperature, the mixture was diluted with water (20 mL) and extracted with DCM (2 x 20 mL). The combined organic layer was dried over Na_2SO_4 and evaporated to give a residue, which was then suspended in NaOH (1 N, 30 mL) and DCM (20 mL). The aqueous layer was carefully acidified with HCl (10%) to pH = 2 and then extracted with DCM (3 x 15 mL). The organic layers were combined, dried over Na_2SO_4, and then evaporated to give 6-chloro-3,4-dihydro-2H-1-benzothiopyran-4-carboxylic acid (1.44 g, 62.7%)

Step 3. To a mixture of 6-chloro-3,4-dihydro-2H-1-benzothiopyran-4-carboxylic acid (1.0 g, 4.37 mmol) in acetonitrile:H_2O (1:1, 20 mL) was added trichlororuthenium hydrate (19.72 mg, 87.46 µmol). After that, sodium periodate (2.34 g, 10.93 mmol) was added in portions. The reaction mixture was then stirred for 3 h. Solids were filtered and washed with water (10 mL) and ethyl acetate (25 ml). The organic layer was separated, and the aqueous layer was re-extracted with ethyl acetate (2 x 10 mL). The combined organic layer was dried over Na_2SO_4 and evaporated to give a residue, which was then dissolved in acetonitrile (25 mL), and SiliaMetS Thiol Metal Scavengers was added to the solution. The mixture was stirred for 30 min and then filtered. The filtrate was evaporated in vacuo to give 6-chloro-1,1-dioxo-3,4-dihydro-2H-1lambda6-benzothiopyran-4-carboxylic acid (940.0 mg, 82.5%).

Step 4. DIPEA (86.76 mg, 671.32 µmol) was added to a mixture of 6-chloro-1,1-dioxo-3,4-dihydro-2H-1lambda6-benzothiopyran-4-carboxylic acid (50.0 mg, 191.8 µmol), isoquinolin-4-amine hydrochloride (41.58 mg, 230.17 µmol), and HATU (109.39 mg, 287.71 µmol) in DMF (2 mL). The reaction mixture was

stirred at room temperature overnight. The reaction progress was checked by LCMS. After consumption of starting material, the reaction mixture was subjected to prepHPLC to give 6-chloro-N-(isoquinolin-4-yl)-1,1-dioxo-3,4-dihydro-2H-1lambda6-benzothiopyran-4-carboxamide (42.8 mg, 57.7%).
MS (ESI+) m/z calculated for $C_{19}H_{16}ClN_2O_3S^+$ ([M+H]$^+$) 387.1, found: 387.2.
Separation condition:

Column: Chromatorex 18 SMB100-5T 100x19 mm 5 µm,
Mobile phase: 20-35% in 0-6 min, H_2O/acetonitrile)
Flow rate: 30 mL/min

6,7-dichloro-N-(isoquinolin-4-yl)-4-methyl-1,2,3,4-tetrahydroquinoline-4-carboxamide

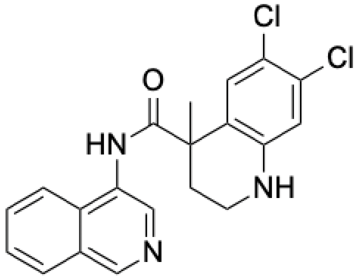

Step 1. Methyl 6,7-dichloro-1,2,3,4-tetrahydroquinoline-4-carboxylate (1.98 g, 7.6 mmol) in anhydrous THF (20 mL) was added dropwise at -30 °C under a nitrogen atmosphere to a solution of lithium diisopropylamide (1 equiv.) in THF. The mixture was stirred at room temperature for 1 h. After that, iodomethane (1.62 g, 11.4 mmol, 710.0 µL, 1.5 equiv.) was added at -50 °C to the mixture, and then the reaction was stirred at room temperature overnight. The reaction mixture was quenched with saturated aqueous NH_4Cl and extracted with ethyl acetate. The organic layer was washed with saturated brine, dried over anhydrous Na_2SO_4, and evaporated in vacuo to give a residue, which was purified by column chromatography (SiO_2, hexane/ethyl acetate) to give methyl 6,7-dichloro-4-methyl-1,2,3,4-tetrahydroquinoline-4-carboxylate (400.0 mg, 19.2%).

Step 2. Methyl 6,7-dichloro-4-methyl-1,2,3,4-tetrahydroquinoline-4-carboxylate (400.0 mg, 1.46 mmol) was dissolved in methanol (15 mL), and then sodium hydroxide (70.12 mg, 1.75 mmol) in water (8 mL) was added to the solution. The obtained mixture was stirred at room temperature overnight, concentrated in vacuo to give sodium 6,7-dichloro-4-methyl-1,2,3,4-tetrahydroquinoline-4-carboxylate (400.0 mg, 97.5%)

Step 3. To a suspension of sodium 6,7-dichloro-4-methyl-1,2,3,4-tetrahydroquinoline-4-carboxylate (400.0 mg, 1.42 mmol) in dioxane (8 mL) and H_20 (8 mL) was added sodium hydrogen carbonate (357.32 mg, 4.25 mmol) and prop-2-en-1-yl carbonochloridate (256.34 mg, 2.13 mmol, 230.0 µL, 1.5 equiv.). After stirring overnight at room temperature, the reaction mixture was washed with methyl tertiary-butyl ether. Hydrochloric acid was then added to the water layer, and the mixture was extracted with ethyl acetate. The organic layer was washed with saturated brine, dried over anhydrous Na_2SO_4, and evaporated in vacuo to give 6,7-dichloro-4-methyl-1-[(prop-2-en-1-yloxy)carbonyl]-1,2,3,4-tetrahydroquinoline-4-carboxylic acid (300.0 mg, 61.5%).

Step 4. HATU (321.66 mg, 845.97 µmol) was added to a mixture of 6,7-dichloro-4-methyl-1-[(prop-2-en-1-yloxy)carbonyl]-1,2,3,4-tetrahydroquinoline-4-carboxylic acid (253.2 mg, 735.63 µmol), isoquinolin-4-amine (106.06 mg, 735.63 µmol), and ethylbis(propan-2-yl)amine (237.44 mg, 1.84 mmol, 320.0 µL, 2.5 equiv.) in DMF (10 mL). The obtained mixture was stirred at room temperature overnight, then poured into water, and extracted with ethyl acetate. The organic layer was washed with saturated brine, dried over anhydrous Na_2SO_4, and evaporated in vacuo to give a residue, which was purified

by HPLC (Chromatorex (18 SMB100-5T 100x19 mm), H_2O/acetonitrile) to give prop-2-en-1-yl 6,7-dichloro-4-[(isoquinolin-4-yl)carbamoyl]-4-methyl-1,2,3,4-tetrahydroquinoline-1-carboxylate (14.0 mg, 4%) .

Step 5. To a solution of prop-2-en-1-yl 6,7-dichloro-4-[(isoquinolin-4-yl)carbamoyl]-4-methyl-1,2,3,4-tetrahydroquinoline-1-carboxylate (14.0 mg, 29.77 µmol) in DCM (5 mL) under argon were added $Pd(PPh_3)4$ (3.45 mg, 2.98 µmol) and morpholine (5.2 mg, 59.69 µmol, 10.0 µL, 2.0 equiv.) . After stirring overnight at room temperature, the obtained mixture was concentrated in vacuo and then subjected to separation (Chiralpak AS-H (250*20 mm, 5 µm), hexane-IPA-MeOH) without additional work-up to give 6,7-dichloro-N-(isoquinolin-4-yl)-4-methyl-1,2,3,4-tetrahydroquinoline-4-carboxamide (9.91 mg, 86.2% yield).
MS (ESI+) m/z calculated for $C_{20}H_{18}Cl_2N_3O^+$ ([M+H]) 386.1, found: 386.0.

6-chloro-N-(isoquinolin-4-yl)-3-methyl-3,4-dihydro-2H-1-benzopyran-4-carboxamide

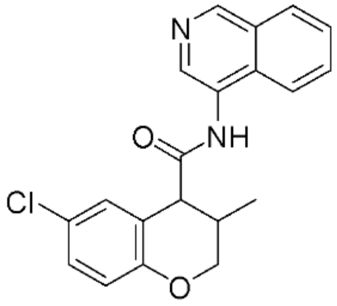

EDJ-MED-e4b030d8-11

To a solution of 6-chloro-3-methyl-3,4-dihydro-2H-1-benzopyran-4-carboxylic acid (1.1 g, 4.85 mmol) in DMF (5 mL) were added isoquinolin-4-amine hydrochloride (876.51 mg, 4.85 mmol), DIPEA (1.25 g, 9.7 mmol, 1.69 mL, 2.0 equiv.) and HATU (2.03 g, 5.34 mmol), and the reaction mixture was stirred overnight at room temperature. Water (30 mL) was then added to the reaction, and the solution was extracted with ethyl acetate (4 x 50 mL). The combined organic layers were washed with brine, dried with sodium sulfate, filtered, and evaporated in vacuo to give a residue, which was purified by flash chromatography using a CombiFlash to afford 6-chloro-N-(isoquinolin-4-yl)-3-methyl-3,4-dihydro-2H-1-benzopyran-4-carboxamide (1.2 g, 93.0% purity, 65.2%).
^{1}H NMR (600 MHz, DMSO-d_6) δ (ppm) 10.42 (s, 1H), 9.12 (s, 1H), 8.66 (s, 1H), 8.18-8.11 (m, 2H), 7.90-7.88 (m, 1H), 7.73-7.71 (m, 1H), 7.32 (s, 1H), 7.19-7.17 (m, 1H), 6.83 (d, *J* = 6.0 Hz, 1H), 4.31-4.28 (m, 1H), 4.10 - 4.07 (m, 2H), 2.32-2.30 (m, 1H), 1.07 (d, *J* = 8.4 Hz, 3H).
MS (ESI+) m/z calculated for $C_{20}H_{18}ClN_2O_2^+$ ($[M+H]^+$) 353.1, found: 353.0.

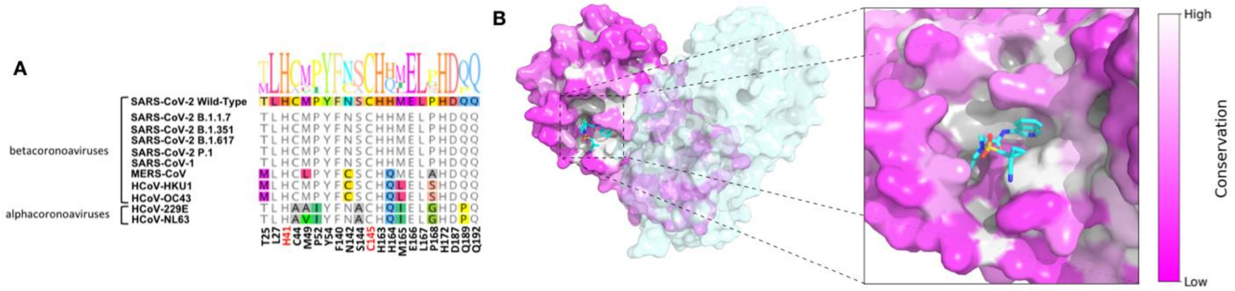

Fig. S1. The SARS-CoV-2 main viral protease (Mpro) is highly conserved across coronaviruses. A. Mpro sequences across coronaviruses are highly conserved due to their requirement to cleave viral polyproteins in numerous locations, showing very little variation in residues lining the active site near the scissile bond. **B.** Available structural data for Mpro from multiple coronaviruses shows a high degree of sequence and structural conservation, especially in the vicinity of the active site.

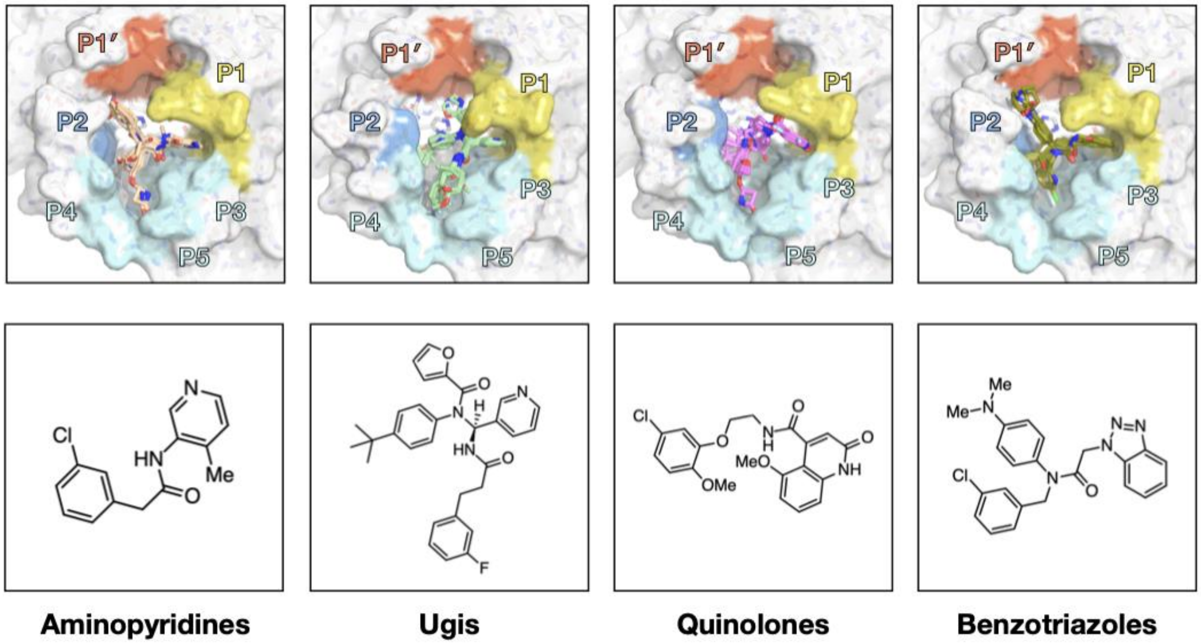

Fig. S2. Initial crowdsourcing efforts produced structures and hits with biochemical potency for four distinct chemical series. Ugi, quinolones and benzotriazoles were based on literature compounds for SARS-CoV main protease. *Top:* Early representative X-ray structures of each series. *Bottom*: Representative compounds from each series.

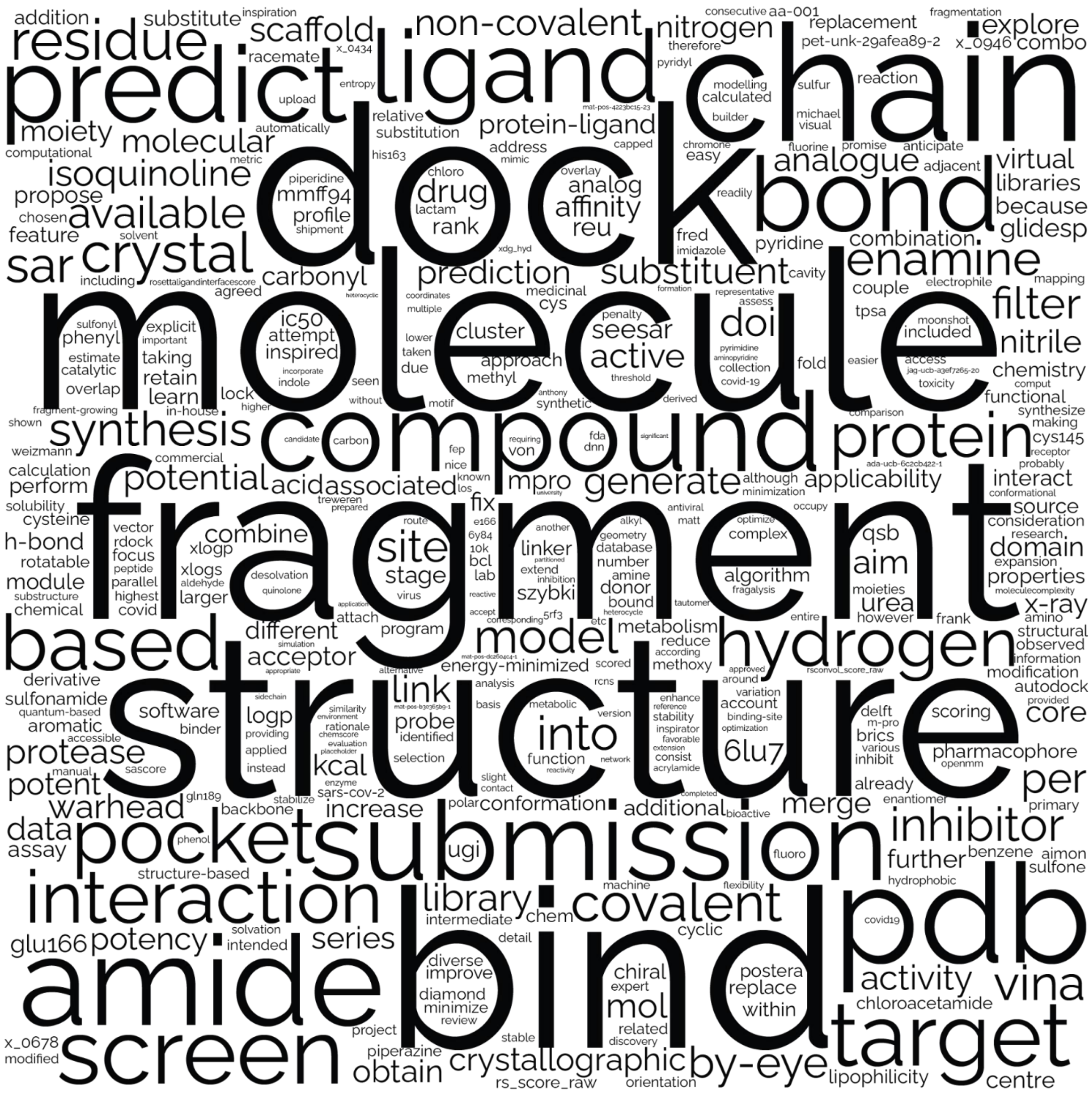

Fig S3. Word cloud representation of crowdsourced submission description. Representation of word frequency from 2,127 crowdsourced submission descriptions. Although this is just a qualitative assessment, some trends may emerge (beyond the obvious theme of **structure** based **fragment** optimization): any of the predictions relied on docking (“**dock**”, “**vina**”); fewer were “**by-eye**”; several of the predictions attempted to “**link**”, “**combine**”, or “**merge**” fragments.

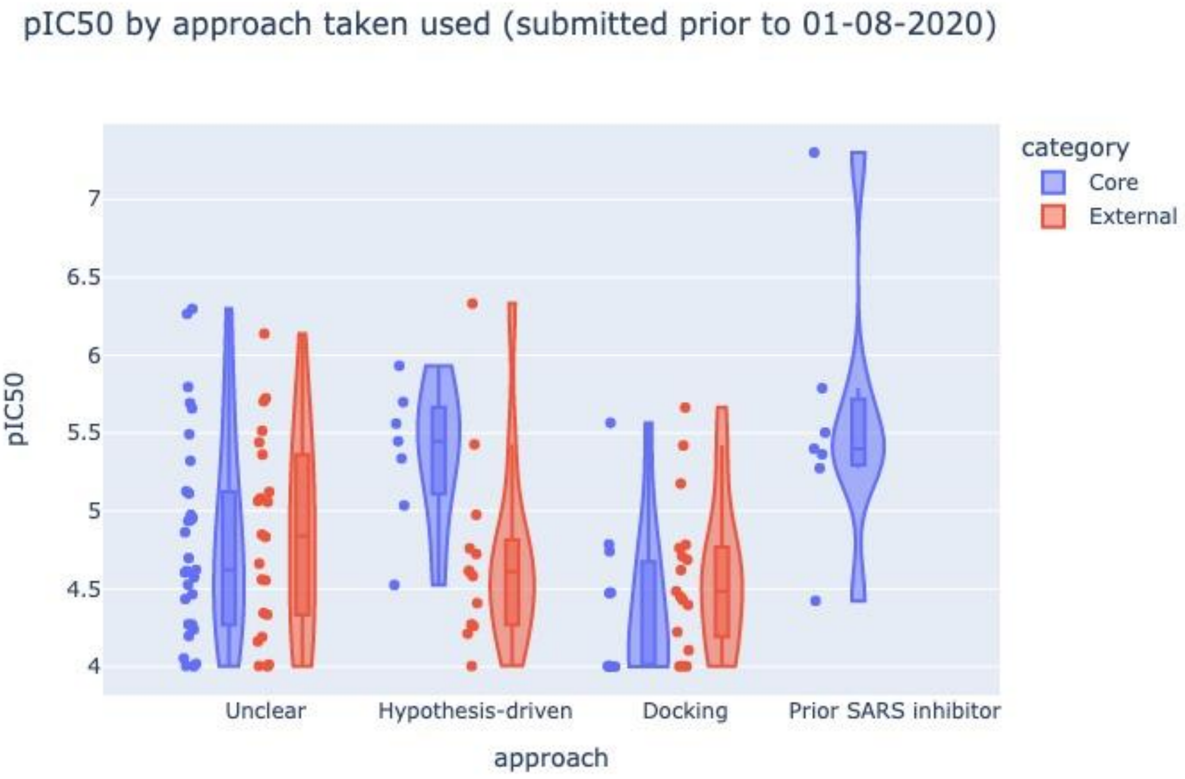

Fig S4. Breakdown of early submitted designs by methods. We attempted to evaluate if a specific 'design method' was more successful based on the descriptions of early designs. It is clear the historical SARS inhibitors perform the best (therefore it might be best to start from a known inhibitor when one is available). Other approaches, however, do not give a clear signal. While hypothesis-driven designs seem to be more potent than 'docking' driven designs, this was driven mainly by a group of covalent designs submitted by the core group.

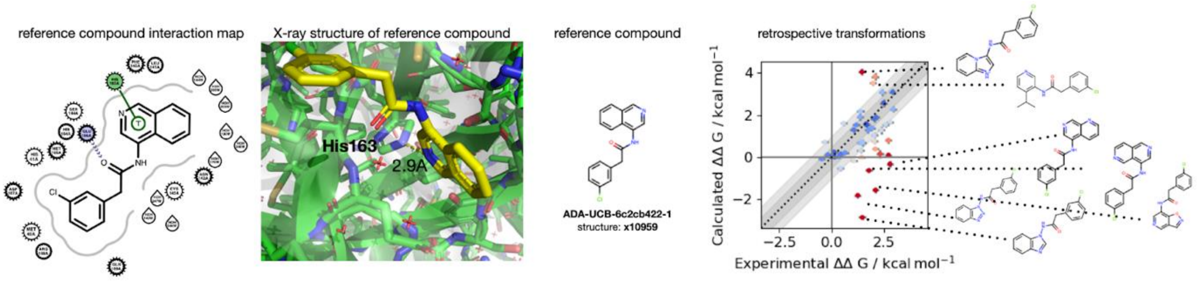

Fig S5. Outliers in retrospective alchemical free energy calculations drove new chemical insight that informed improvements in modeling strategy. Alchemical free energy calculation <u>Sprint 10</u> considered transformations proposed from **ADA-UCB-6c2cb422-1** as a reference compound. <u>Retrospective calculations</u> included in the batch showed excellent correspondence for many compounds, as well as a near-vertical band of outliers with no correlation with experiment. Examination of the outliers in this band (right) revealed they shared conservative modifications to the quinoline scaffold engaging P1 that made very small changes to steric contacts but significantly modified the pKa of the nitrogen engaging His163, suggesting we needed to consider multiple protonation state variants of both His163 and the part of the compound engaging it in hydrogen bonding. Future iterations of free energy calculations considered multiple protonation states of His163 and design compounds.

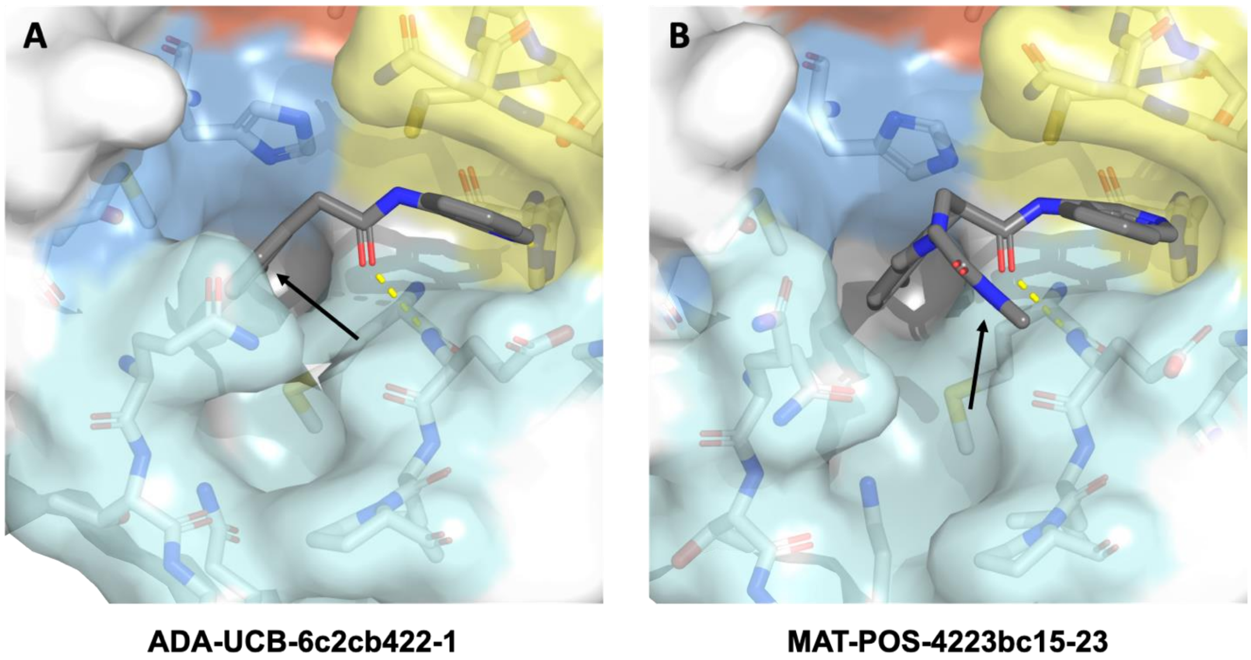

Fig S6. Parent compounds for HTC optimization. A. Structure of the precursor for the Chan-Lam reaction, in which we attempted to functionalize Position C5 of the chloro-phenyl (marked with an arrow) to expand into the P3-P5 pockets (cyan). PDB ID: 7GLP; Resolution 1.92Å **B.** Structure of an example of a methyl-derived amide for the parent acid **MAT-POS-4223bc15-21** (for which we were not able to get a good co-crystal structure, possibly due to its relatively weak affinity). We attempted to derivatize the amide bond to expand towards the P3-P5 pockets (cyan). PDB ID: 7GKV; Resolution 1.88Å.

A

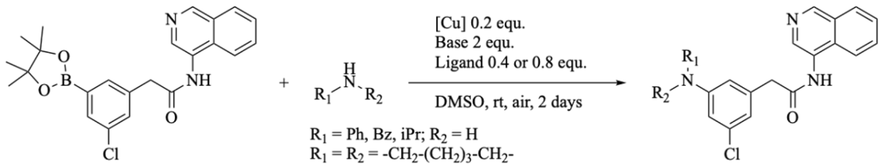

B

Ligand	BiPy				TMP				DMAP			
Base	Py	Et3N	DBU	DABCO	Py	Et3N	DBU	DABCO	Py	Et3N	DBU	DABCO
Aniline	<1	<1	<1	4	<1	<1	<1	3	16	15	4	21
Benzylamine	12	10	17	12	7	4	10	6	32	35	18	38
Isopropylamine	1	4	4	5	1	1	2	2	17	30	2	22
Piperidine	21	10	6	18	8	10	1	5	57	54	3	48

C

	Cu(OAc)2*H2O	CuI	CuCl
Aniline	1	10	13
Benzylamine	12	32	44
Isopropylamine	22	67	49
Piperidine	39	65	67

D

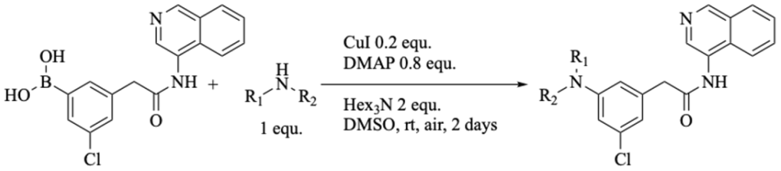

Fig S7. Optimization of HTC Chan-Lam Reaction. A. Model reaction scheme. **B.** Initial reaction condition screening was performed to find the most suitable base (Py: Pyridine; Et_3N: triethylamine; DBU: 1,8-Diazabicyclo(5.4.0)undec-7-ene; DABCO: 1,4-diazabicyclo[2.2.2]octane) and ligand (BiPy: Bipyridine; TMP: 3,4,7,8-Tetramethyl1,10-phenanthroline; DMAP: 4-Dimethylaminopyridine) for the synthesis of arylamines using DMSO as a solvent, atmospheric oxygen as an oxidant, and Cu(OAc)2*H_2O as a copper source. The experiment was done in a 384-well plate and the reactants were arrayed manually. We report estimated yields (%). Under the tested conditions, the yields correlate with the nucleophilicity of the tested model amines. The highest yields were obtained using DMAP and Et_3N. **C.** We investigated the effect of copper source on product yield, using the previously optimized reaction conditions (DMAP, Et_3N). There was no significant difference between copper-iodide (CuI) and copper-chloride (CuCl), thus we decided to proceed with CuI since it is a known promoter for this reaction in combination with DMAP (*94*). **D.** The final optimization step included switching the pinacol boronic ester to an unprotected boronic acid as well as the Et_3N to trihexylamine - a less volatile homolog of Et_3N, which yielded 76% product formation when piperidine was used as the model amine, without phenol or homocoupling side-products and 4% of protodeboronated starting material. These reaction conditions were used for library synthesis.

A

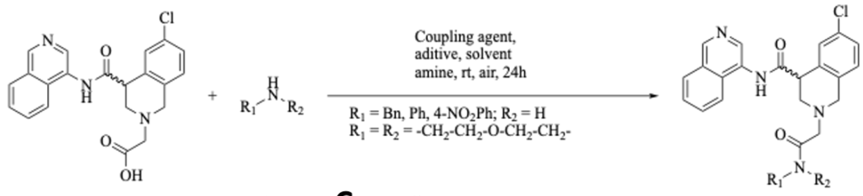

B

	DIPEA, DMSO		
	DMTMM	HATU	EDC
Benzylamine	2	49	22
Aniline	10	19	22
Morpholine	26	44	overlap
sBuNH2	11	16	0

C

	DIPEA, DMSO			
acid:HATU:amine	1:1.5:1.5	1:2:2	1:1:1.5	1:1:2
Benzylamine	22	5	31	15
Aniline	28	33	58	54
Morpholine	11	3	15	6
sBuNH2	58	41	65	58

D

	EDC, DIPEA, DMSO				
	HOAt	HOBt	OSu	HOAt DMAP	HOBt DMAP
Aniline	86	85	44	95	68
Benzylamine	84	85	89	85	86
p-nitroaniline	0	0	0	0	0
Morpholine	93	92	92	92	92

E

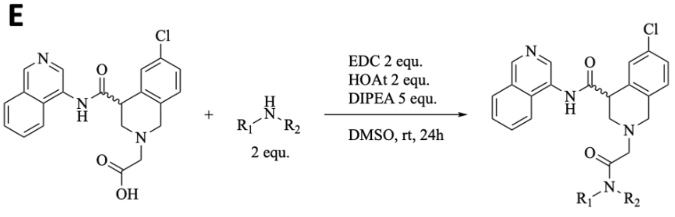

Fig S8. Optimization of HTC amide coupling. A. Model reaction scheme. **B.** We investigated suitable coupling agents. Reactions in DMSO, with equimolar amounts of DIPEA (N,N-Diisopropylethylamine), coupling agent (DMT-MM: 4-(4,6-dimethoxy-1,3,5-triazin-2-yl)-4-methyl- morpholinium chloride; EDC: 1-Ethyl-3- (3-dimethylaminopropyl) carbodiimide HCl; HATU: 1-[Bis(dimethylamino) methylene]-1H-1,2,3-triazolo[4,5-b] pyridinium 3-oxide hexafluorophosphate), model amine (aniline, benzylamine, morpholine, and sec-butylamine), and carboxylic acid each. After pre-activating the acid with the coupling agent for five minutes, model amines were arrayed to each well. After overnight incubation, each well was diluted with 50% ACN (Acetonitrile) in water, and the yields were estimated from the UV chromatograms. We report estimated yields (%). In all cases, unreacted acid was observed. **C.** With higher ratios of coupling agent and base, the yields did increase, but uronium modified starting material was observed**.** We tried repeating the reaction in DMA (Dimethylacetamide) or NMP (N-methylpyrrolidone), but the yields were even lower (data not shown). **D.** We increased the amount of DIPEA to 5 equ. and replaced HATU with EDC to avoid formation of side products. Using EDC, we observed 100% acid consumption. To avoid isomerization of the activated acid to unreactive N-acylurea, we explored the use of additives (HOAt: 1-Hydroxy-7-azabenzotriazole; HOBt: Hydroxybenzotriazole; OSu:N,N-Disuccinimidyl carbonate). We report estimated yields (%). **E.** Since DMAP did not significantly improve the yield, we decided to proceed with HOAt as an additive to simplify the UV chromatograms. These reaction conditions were used for library synthesis.

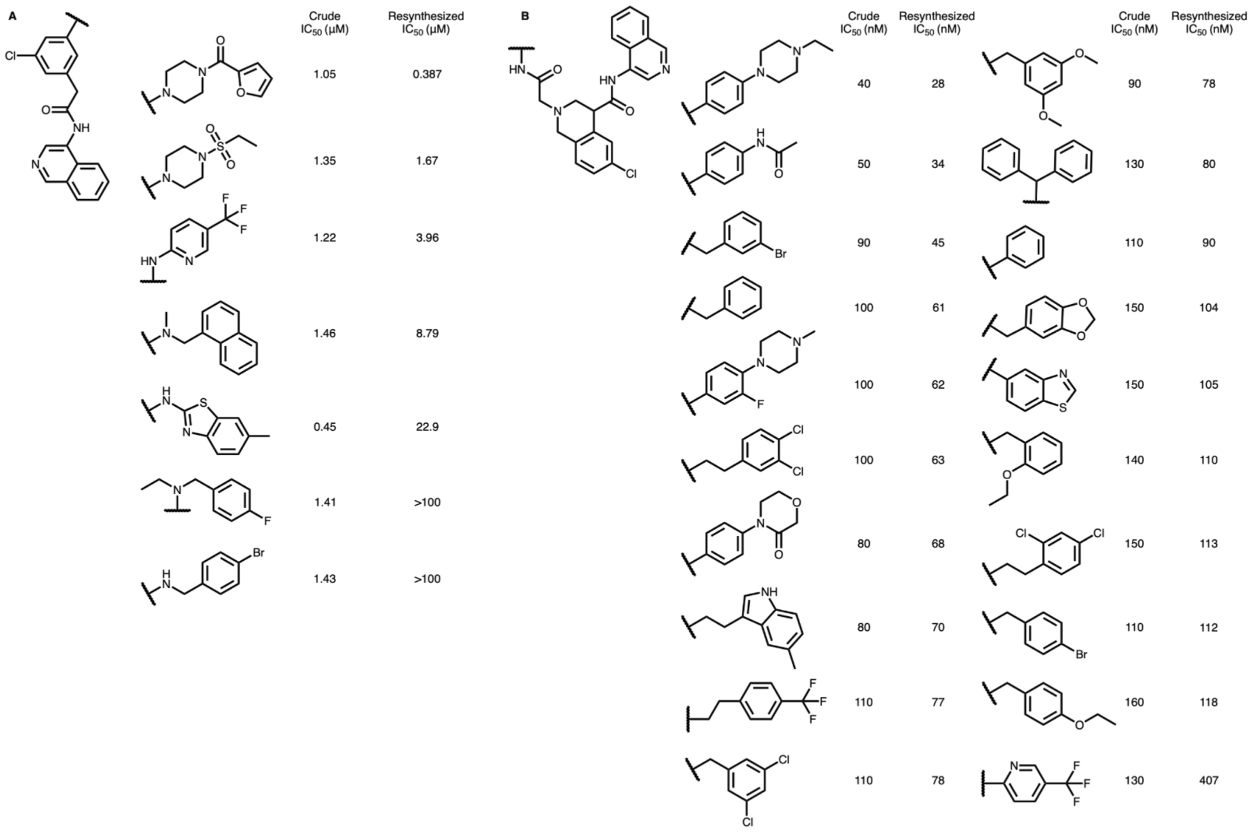

Fig S9. HTC optimization of Mpro inhibitors towards P3-5. A. Selected Chan-Lam installed derivatives of **ADA-UCB-6c2cb422-1** and **B.** Amide coupling derivatives of **MAT-POS-4223bc15-23** were tested both as crude reaction mixtures, as well as pure resynthesized compounds in the Mpro biochemical assay.

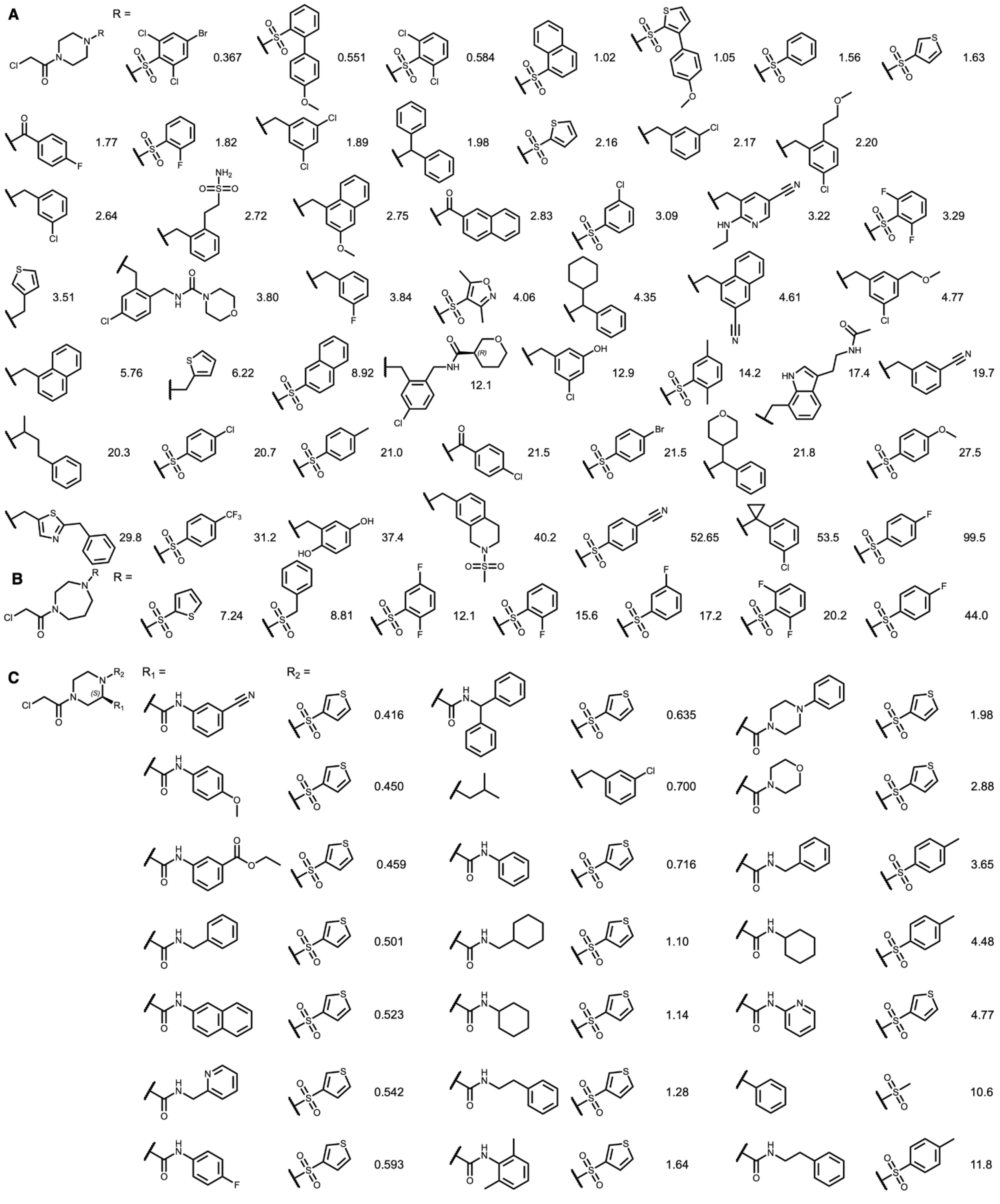
A
R =
0.367 0.551 0.584 1.02 1.05 1.56 1.63
1.77 1.82 1.89 1.98 2.16 2.17 2.20
2.64 2.72 2.75 2.83 3.09 3.22 3.29
3.51 3.80 3.84 4.06 4.35 4.61 4.77
5.76 6.22 8.92 12.1 12.9 14.2 17.4 19.7
20.3 20.7 21.0 21.5 21.5 21.8 27.5
29.8 31.2 37.4 40.2 52.65 53.5 99.5
B
R =
7.24 8.81 12.1 15.6 17.2 20.2 44.0
C
R1 =
R2 =
0.416 0.635 1.98
0.450 0.700 2.88
0.459 0.716 3.65
0.501 1.10 4.48
0.523 1.14 4.77
0.542 1.28 10.6
0.593 1.64 11.8

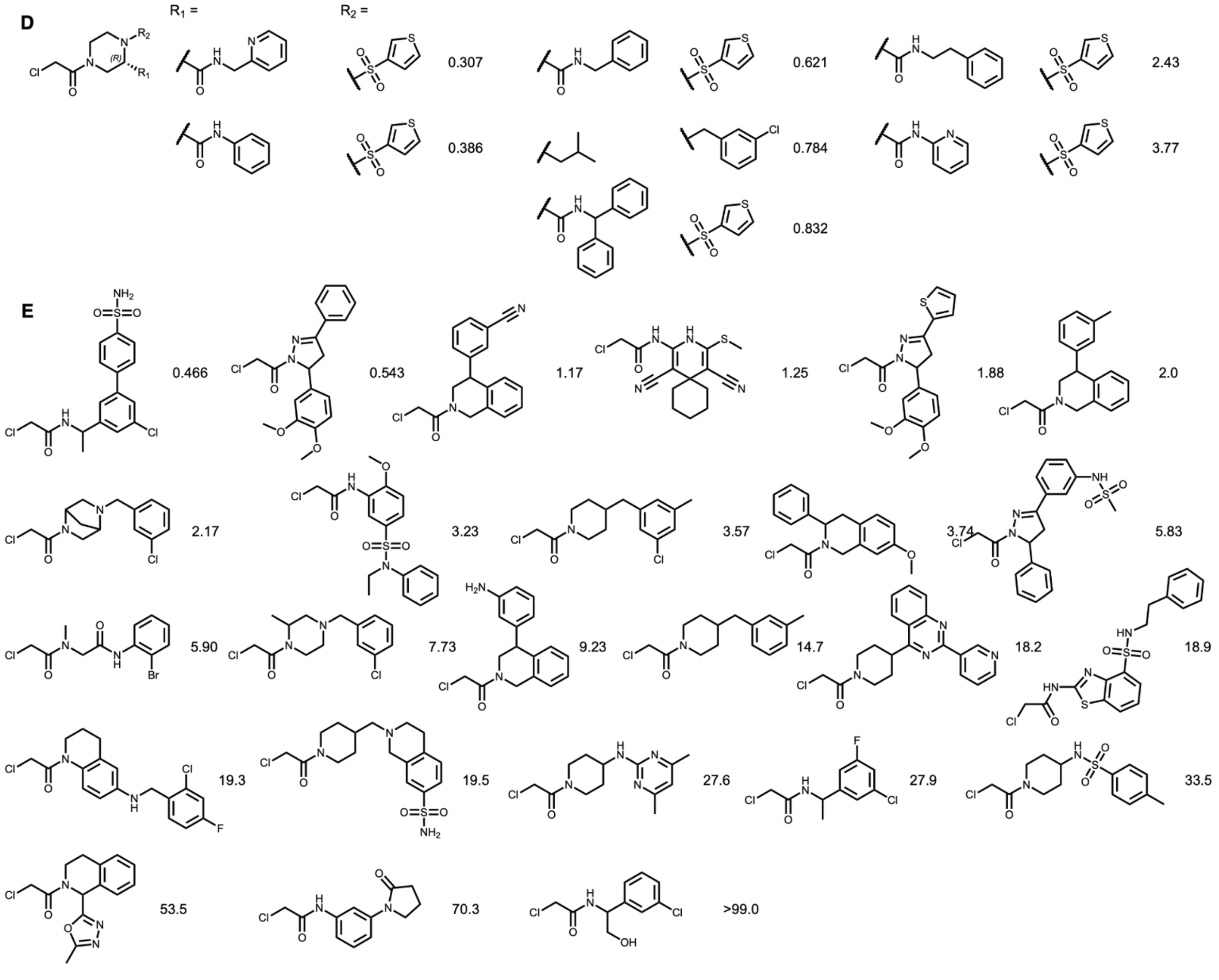

Fig S10. Chloroacetamide fragment optimization. Measured IC_{50} are reported in µM. **A.** Optimization of a piperazine chloroacetamide series **B.** Optimization of a diazepane chloroacetamide series **C.** Further derivatization of the (s) isomer of the piperazine chloroacetamides **D.** Further derivatization of the (r) isomer of the piperazine chloroacetamides **E.** Singelton chloroacetamides tested.

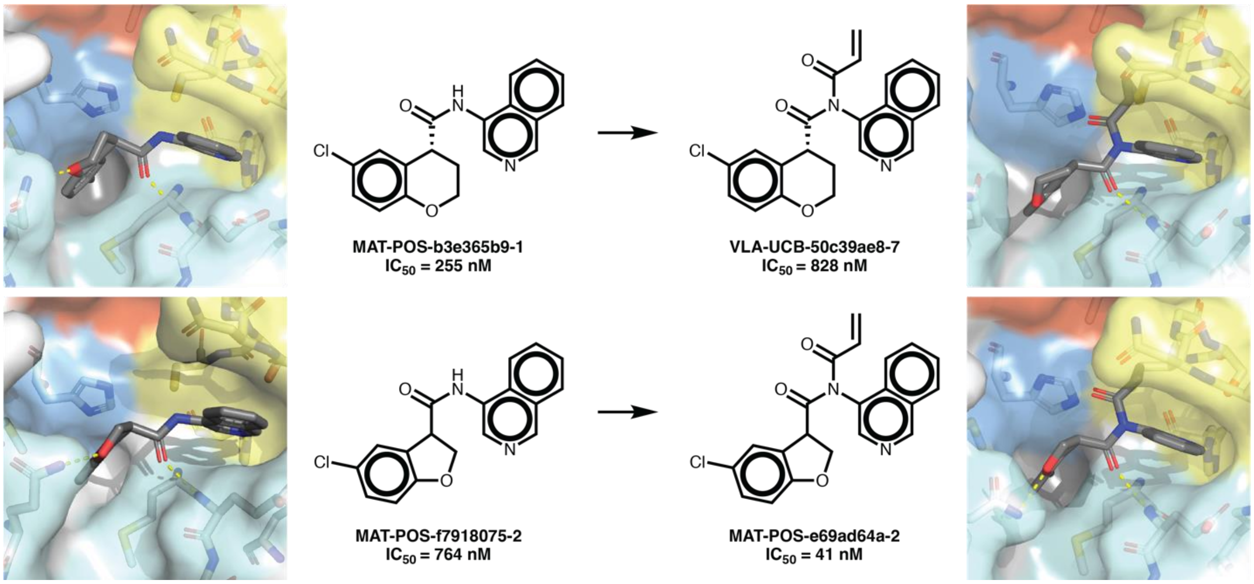

Supplementary Figure 11: Covalentization of potent binders leads to a 40 nM Mpro inhibitor. The structure of **MAT-POS-b3e365b9-1** (top left; PDB: 7GFB) suggested that installation of an acrylamide moiety off the central amide might be able to engage covalently with the catalytic Cys145. While the acrylamide version **VLA-UCB-50c39ae8-7** proved 3-fold less potent, the co-crystal structure showed it was able indeed to form the designed covalent bond (top right; PDB: 7GJ7). Moreover, similar covalentization of a close analog (**MAT-POS-f7918075-2**; bottom left; PDB: 7GAV**)** led to **MAT-POS-e69ad64a-2** (bottom right; PDB: 7GJE) with almost 19-fold improvement in IC_{50} down to 41 nM. This suggests that very slight modification of the overall binding pose can lead to dramatic rate enhancements as they place the electrophile more accurately for nucleophilic attack by the cysteine.

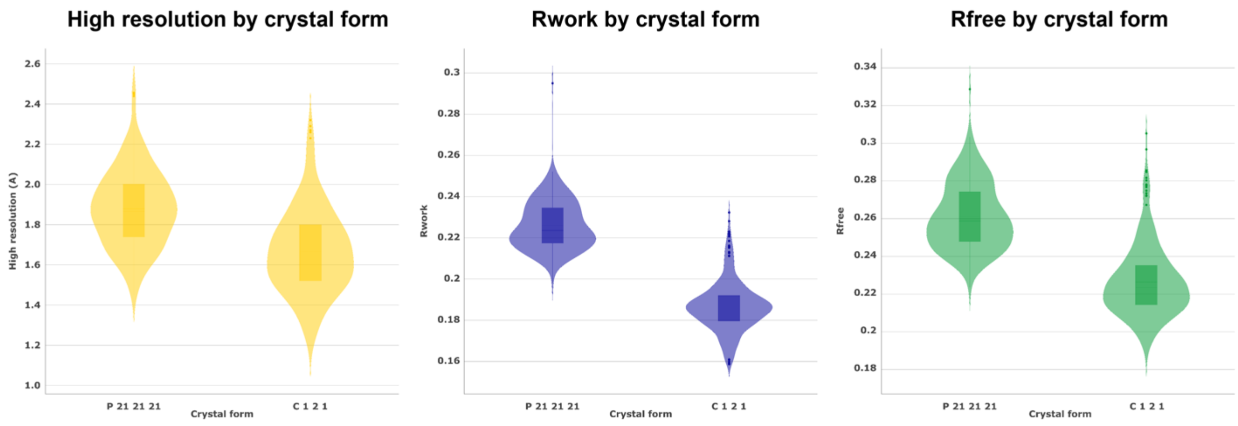

Supplementary Figure 12: Distribution of Crystallographic statistics over collected Mpro datasets in two crystal forms. See **Data S4** for a breakdown by structure.

Supplementary Figure 13: Electron density maps for ligands highlighted in this manuscript. 2FoFc maps (blue) and PanDDA event maps (orange). See **Data S4** for PDB IDs.

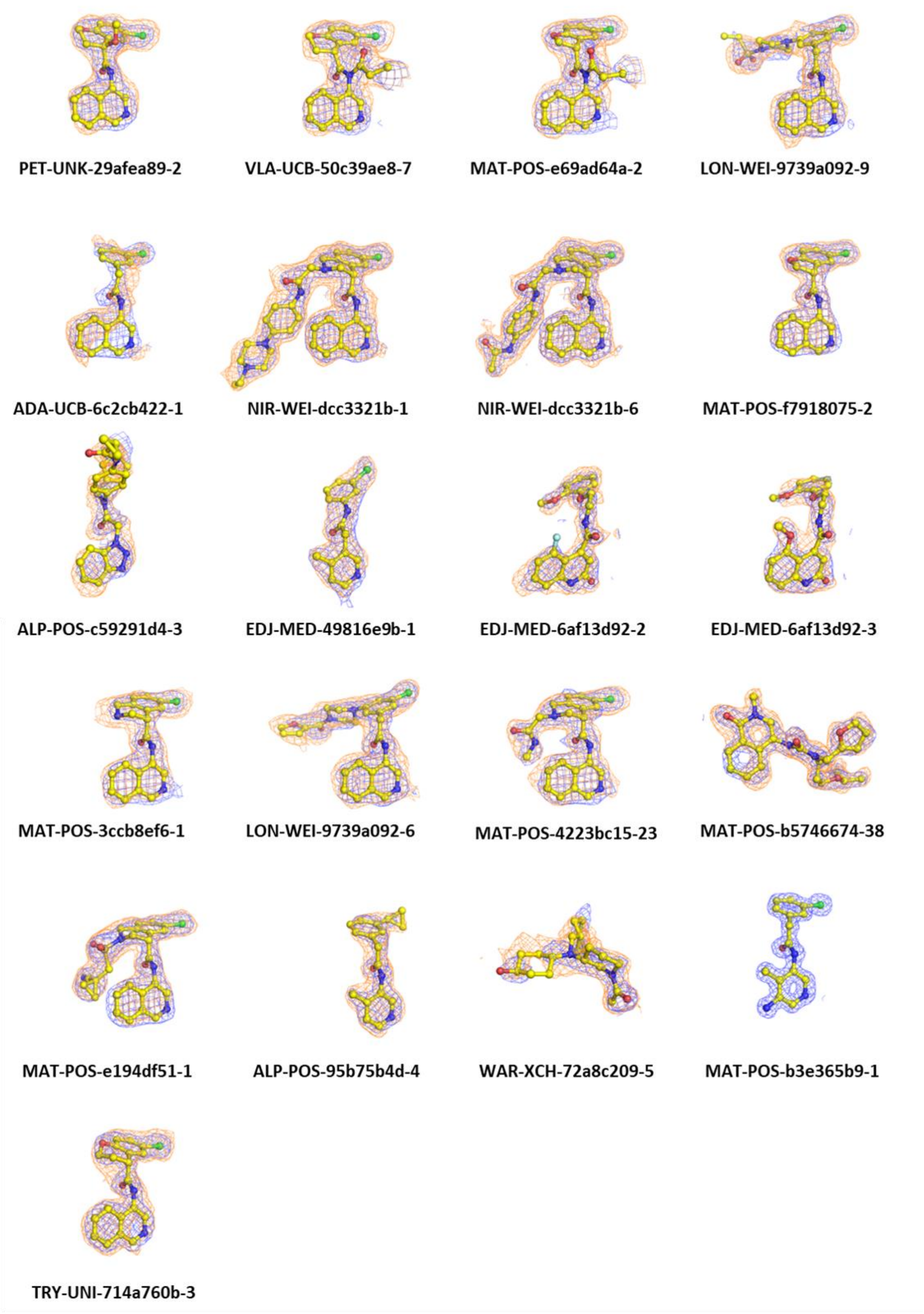

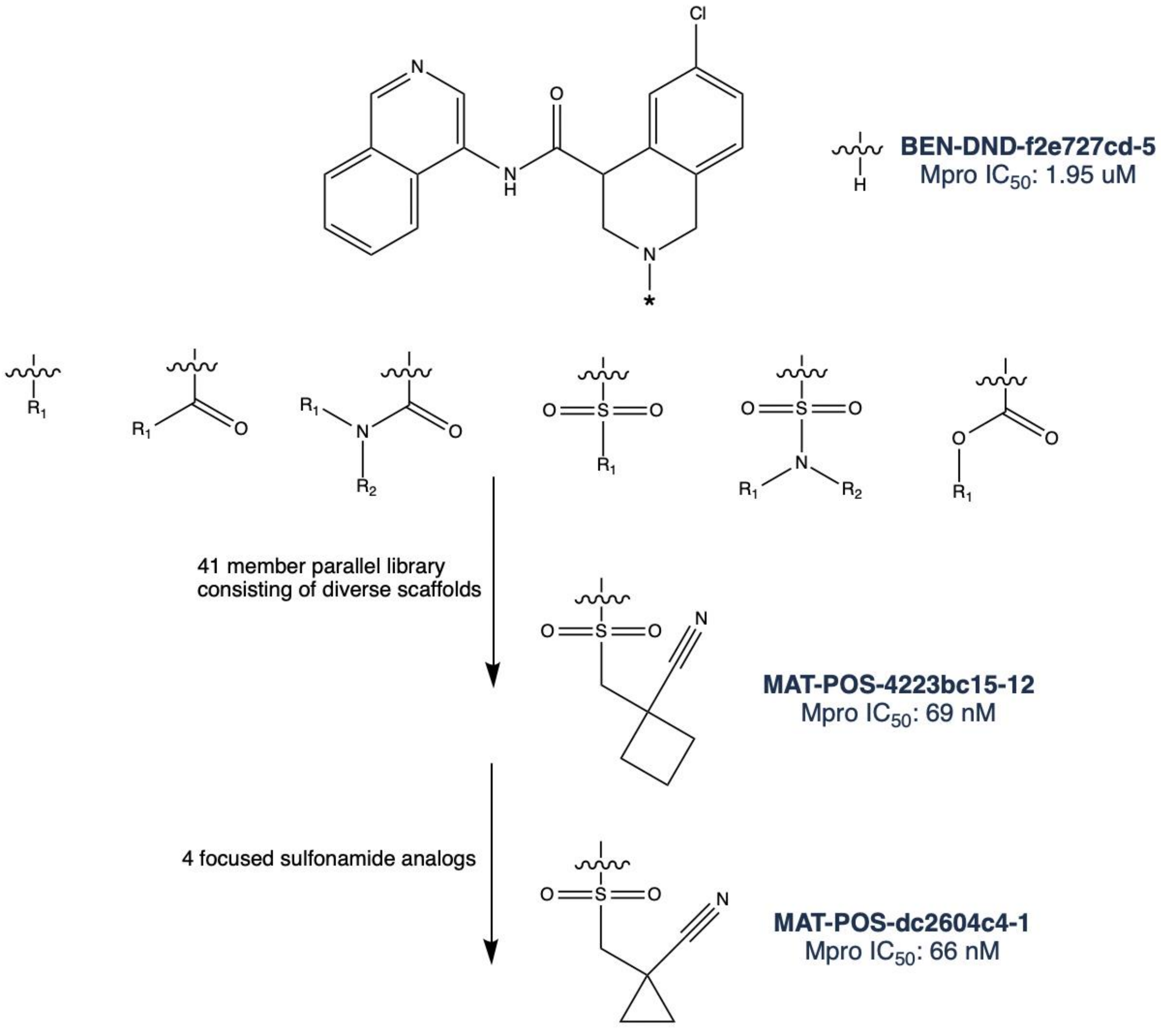

Supplementary Figure 14: A diverse library of potential designs accessible through robust, parallel chemistry was designed using the amine functionality of BEN-DND-f2e727cd-5 (racemate of MAT-POS-3ccb8ef6-1) as the reactive handle. The library consisted of targets accessible via standard reactions such as amide, sulfonamide, and urea couplings from large in-stock monomer classes. The large enumerated libraries were filtered down to a diverse 41 member library MAT-POS-4223bc15, which included the potent sulfonamide MAT-POS-4223bc15-12. More focused exploration of that promising compound from in-stock building blocks (MAT-POS-dc2604c4) then led to advanced compound MAT-POS-dc2604c4-1 (racemate of MAT-POS-e194df51-1).

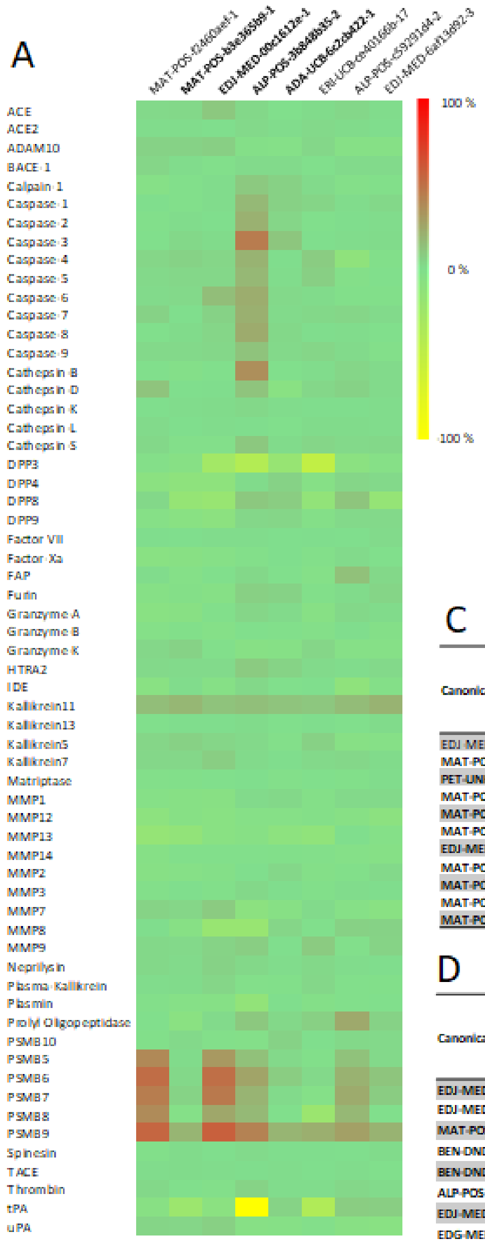

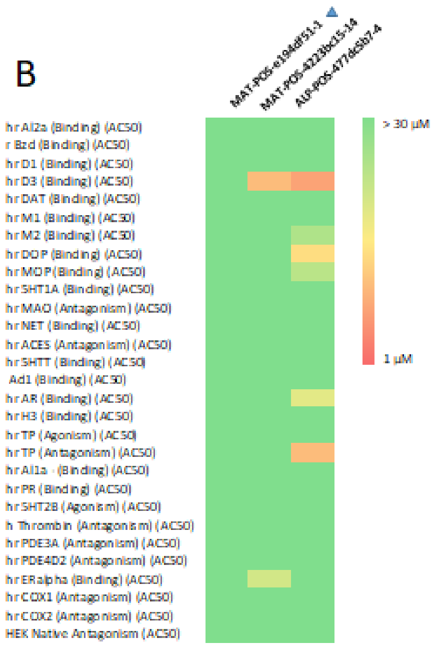

C

Canonical PostEra ID	MDCK-MDR1 P app A>B (10^-6 cm/s)	MDCK-MDR1 P app B>A (10^-6 cm/s)	MDCK-MDR1 Ratio	MDCK-MDR1 Recovery A>B (%)	MDCK-MDR1 Recovery B>A (%)	MDCK-MDR1 ER	MDCK-LE P app A>B (10^-6 cm/s)	MDCK-LE FA (%)	MDCK-LE recovery (%)
EDJ-MED-6af13d92-3	1.96			83.2					
**MAT-POS-b3e365b9-1**	40.8			63.8					
PET-UNK-29afea89-2	20.4	22.2	1.09	77.5	77.9				
MAT-POS-4223bc15-2	16	21	1.31	87.5	84.8				
**MAT-POS-4223bc15-14**	0.517	0.896	1.73	79.2	71.8				
MAT-POS-ec6d90b7-7	11.5	14	1.22	43.1	46.5				
EDJ-MED-1981ceba-3	11.5	29.6	2.58	86.6	72.7				
MAT-POS-dc2604c4-1	1.97	23.4	11.9	74.4	72.8				
**MAT-POS-4223bc15-14**	0.7	0.9	1.29	85	103	1.3	1.6	30.7	93
MAT-POS-dc2604c4-1	0.5	21	42	84	72	38.9	25	94	91
**MAT-POS-e194df51-1** ▲	0.6	17	28.3	72	64	27.6	21.8	92.9	84

D

Canonical PostEra ID	CYP2C9 IC50 (µM)	CYP2C19 IC50 (µM)	CYP2D6 IC50 (µM)	CYP3A4 IC50 (µM)	CYP3A4 TDI k obs (min-1)	CYP3A4 TDI t 1/2 (min)	CYP3A4 TDI rever-sible	CYP3A PXR induction fold	CYP3A PXR induction dose @ max	CYP3A Induction EC50 (uM)	CYP3A PXR Induction Risk
EDJ-MED-50fe53e8-1	> 100			22.4							
EDJ-MED-50fe53e8-2	3.45			2.7							
**MAT-POS-b3e365b9-1**	7.95			2.88							
BEN-DND-c852c98b-5	5.18			4.85							
BEN-DND-4f474d93-1	1.26			5.23							
ALP-POS-ce760d3f-3	> 100			67.7							
EDJ-MED-92e193ae-1	1.46	> 33.3	> 100	11.9							
EDG-MED-5d232de5-1	> 100			< 0.0457							
**MAT-POS-f9802937-7**	21.9			0.422							
**MAT-POS-4223bc15-11**	20.7		7.9	3.3	0.012	28	< 10.0	12.4	57.8	3.2	Moderate
**MAT-POS-4223bc15-14**	0.6		25	15	0.004	28	< 10.0	2.1	5.4		None
EDJ-MED-1981ceba-3	23		20	4.3	0.054	12.8	< 10.0	8.4	37.6	4.2	Low
EDJ-MED-1981ceba-4	23.1		17.7	4.7	0.016	28	< 10.0	5.4	22.3		None
MAT-POS-dc2604c4-1	25		0.6	12.4	0.013	28	< 10.0	9	39.2	4.1	Low
**MAT-POS-e194df51-1** ▲	25.0		9.4	10.3	0.006	28	< 10.0	5.7	31.5	3	Low

Supplementary Figure 15: Visualization of selected safety data of the COVID Moonshot lead series. (A) Protease enzyme selectivity measured at 100 µM (Nanosyn panel) shows high selectivity of COVID Moonshot compounds from different series against a large panel of proteases. Compounds from the aminopyridine lead series are marked in bold throughout Fig S12, with the lead compound marked by a triangle. (B) **MAT-POS-e194df51-1** shows IC_{50}s >30 µM across the Eurofins principle panel (yellow circle), with two other compounds from the aminopyridine series also demonstrating a favorable profile. (C) Permeability data for the

aminopyridine series is detailed, showing a high MDCK-MDR1 efflux ratio (ER) for **MAT-POS-e194df51-1** that suggests low brain penetrance, whilst the high MDCK-LE permeability indicates high intrinsic permeability. (D) CYP reactivity of selected COVID Moonshot compounds, showing variable engagement of Cyp3A4 across the series, but no risk of time dependent inhibition. Further, the risk of CYP3A4 induction via PXR is low. All raw data is available as supplementary material.

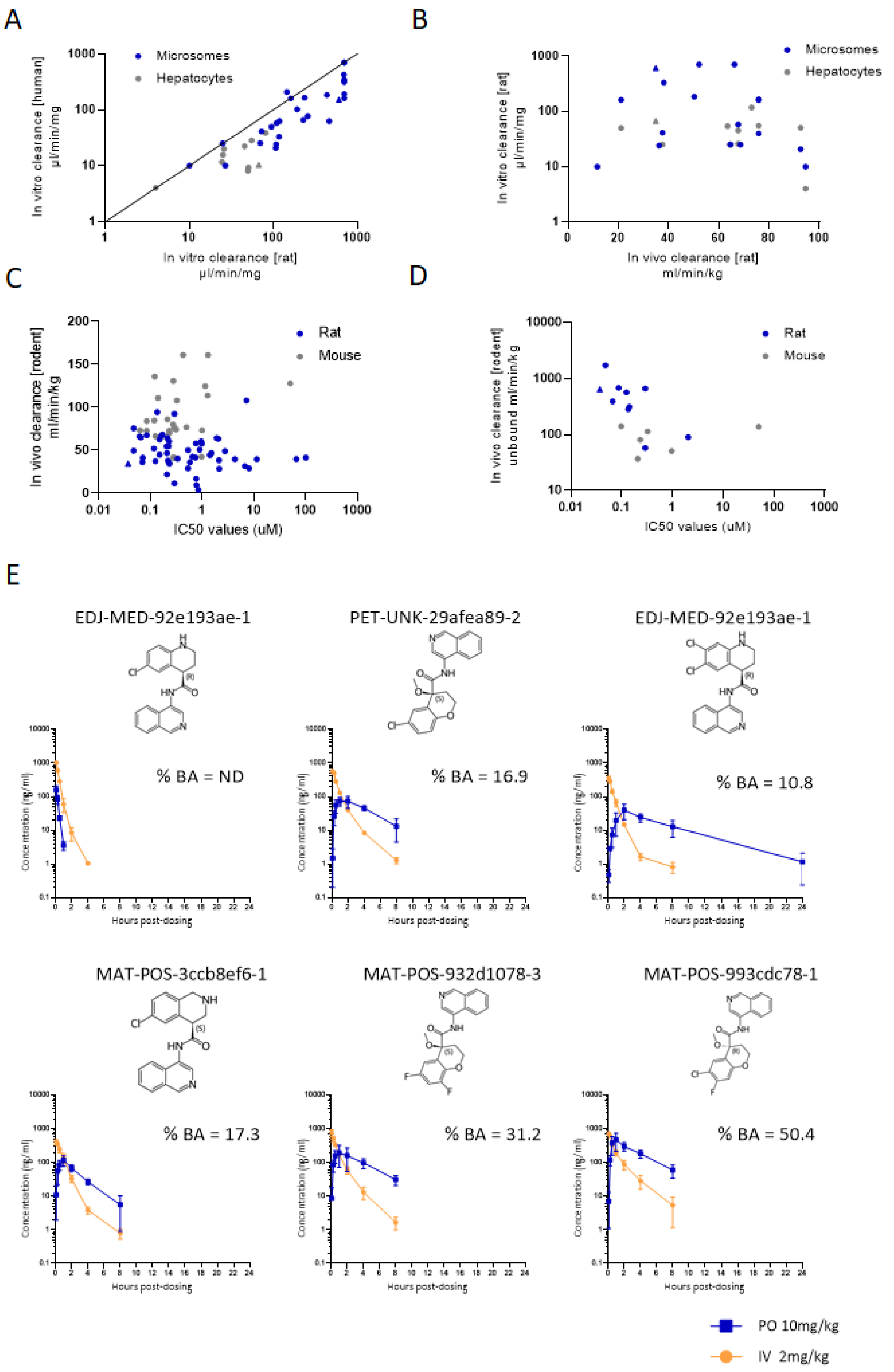
A
In vitro clearance [human]
µl/min/mg
In vitro clearance [rat]
µl/min/mg
Microsomes
Hepatocytes
B
In vitro clearance [rat]
µl/min/mg
In vivo clearance [rat]
ml/min/kg
Microsomes
Hepatocytes
C
In vivo clearance [rodent]
ml/min/kg
IC50 values (uM)
Rat
Mouse
D
In vivo clearance [rodent]
unbound ml/min/kg
IC50 values (uM)
Rat
Mouse
E
EDJ-MED-92e193ae-1
% BA = ND
PET-UNK-29afea89-2
% BA = 16.9
EDJ-MED-92e193ae-1
% BA = 10.8
MAT-POS-3ccb8ef6-1
% BA = 17.3
MAT-POS-932d1078-3
% BA = 31.2
MAT-POS-993cdc78-1
% BA = 50.4
Concentration (ng/ml)
Hours post-dosing
PO 10mg/kg
IV 2mg/kg

Supplementary Figure 16: Visualization of selected ADME, in vitro and in vivo PK data of the COVID Moonshot lead series. (A) Both in vitro clearance measurements in microsomes (blue) and hepatocytes (grey) show a higher clearance in rats than humans. Data suggests dominant Phase 1 metabolism for the aminopyridine series. (B) Linked rat in vitro and rat in vivo clearances are depicted for microsomes and hepatocytes. (C) For all in vivo PK measurements, rat (blue) and mouse (grey) clearance is depicted in relation to fluorescence IC50 values. (D) For compounds with plasma protein binding assessed, unbound clearance is shown for rodent PK experiments. In panels A to D, **MAT-POS-e194df51-1** is marked by a triangle. (E) Rat intravenous (IV, at 2 mg/kg) and oral (PO, at 10 mg/kg) PK experiments show the progression of oral bioavailability (BA) of the aminopyridine lead series. All raw data is available in Data S3.

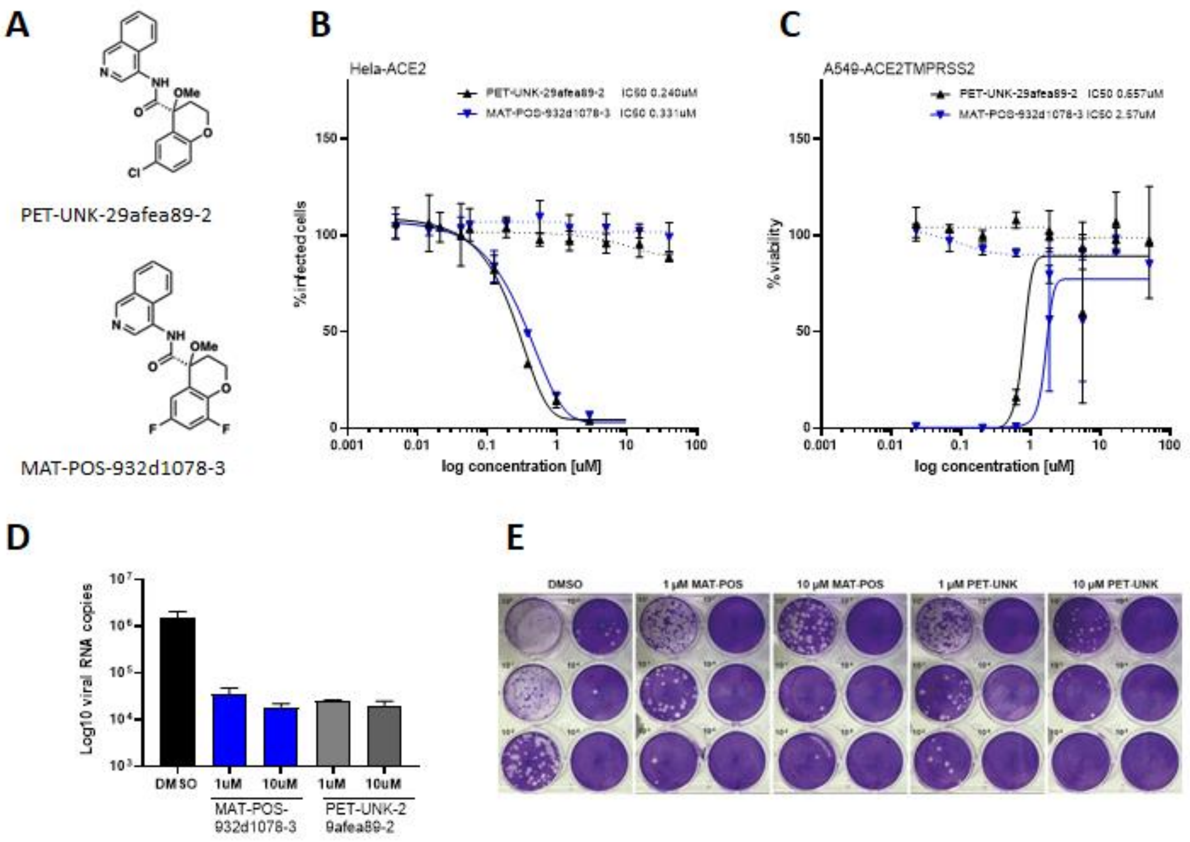

Supplementary Figure 17: Closely related analogues of the lead compound, PET-UNK-29afea89-2 and MAT-POS-932d1078-3, demonstrate antiviral activity across different cellular antiviral assays and a kidney organoid model. **A**: shows the chemical structure of PET-UNK-29afea89-2 and MAT-POS-932d1078-3. **B:** dose-response curves of both compounds in Immunofluorescence assays in Hela-ACE2 cells, and **C**: Cytopathic Effect assays in A549-ACE2-TMPRSS2 cells. The curves also show the cytotoxicity data (dotted lines), demonstrating the lack of cytotoxic activity across all three cell lines. **D-E**: Antiviral activity of MAT-POS-932d1078-3 and PET-UNK-29afea89-2 in kidney organoids infected with SARS-CoV-2 in the presence of 1 µM and 10 µM of compounds or DMSO as a control. **D:** Intracellular viral RNA measured by qPCRand **E**: infectious viral titers released from the apical side of the organoids at 48 hpi, measured by plaque assay on Vero E6 cells. Data in D are mean and SD of 2 biological replicates from a representative experiment of 2 independent experiments. Intracellular viral RNA levels in **D** were normalized to expression of the β-actin housekeeping gene.

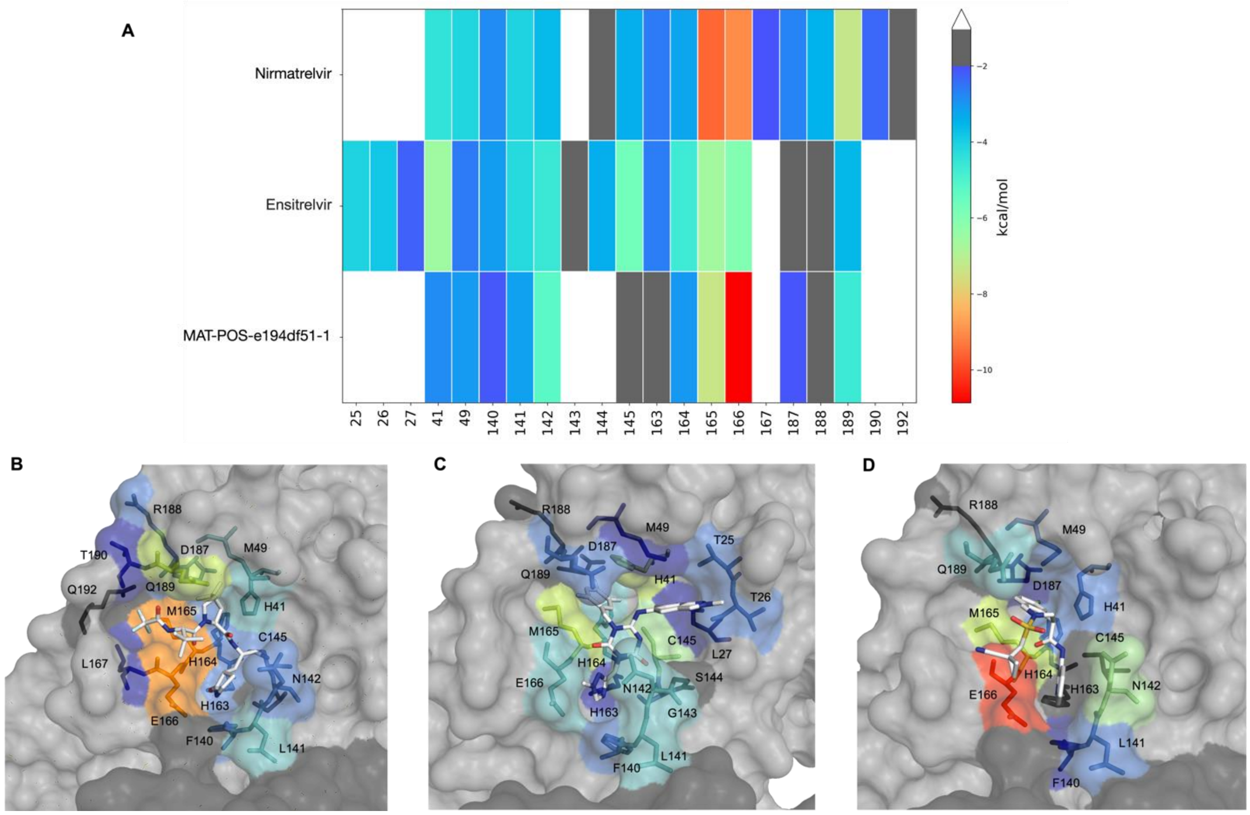

Supplementary Figure 18: **The interaction patterns of our lead compound MAT-POS-e194df51-1 with the Mpro binding site is distinct to known clinical antivirals nirmatrelvir and ensitrelvir (S-217622), thus likely to be active against resistant variants**. (A) The van der Waals interaction energy between the inhibitors and key residues in the Mpro binding site, and the binding poses of (B) nirmatrelvir (PDB ID: 8DZ2), (C) ensitrelvir (S-217622; PDB ID: 8DZ0) and (D) MAT-POS-e194df51-1 (PDB ID: 7GAW). The interaction energy is computed using a method previously reported in ref (*95*) and validated for hepatitis C virus protease.

Supplementary Table 1: **Summary crystallographic statistics for all collected datasets**

Space group	C 1 2 1	P 21 21 21
No. of datasets	359	228
High resolution range (A)	1.18 - 2.84	1.43 - 2.44
Mean high resolution (A)	1.67	1.82
Standard deviation high resolution (A)	0.22	0.17
Mean abs. dev. high resolution (A)	0.17	0.13
Median high resolution (A)	1.65	1.83
Median abs. dev. high resolution (A)	0.14	0.09
Mean Unique reflections (High res shell)	92663 (1508)	78964 (1931)
Mean CCHalf (High res shell)	0.99 (0.33)	1 (0.6)
Mean I/SigI (High res shell)	8.31 (0.68)	9.28 (1.53)
Mean overall completeness (High res shell)	98.03 (93.56)	93.49 (67.18)
Mean Rmerge (High res shell)	0.12 (1.32)	0.15 (1.29)
Mean multiplicity (High res shell)	3.22 (2.76)	6.69 (6.12)
Rwork range	0.16 - 0.23	0.2 - 0.3
Mean Rwork	0.19	0.23
Standard deviation Rwork	0.01	0.01
Mean abs. dev. Rwork	0.01	0.01
Median Rwork	0.19	0.22
Median abs. dev. Rwork	0.01	0.01
Rfree range	0.19 - 0.31	0.22 - 0.33
Mean Rfree	0.23	0.26
Standard deviation Rfree	0.02	0.02
Mean abs. dev. Rfree	0.01	0.01
Median Rfree	0.22	0.26
Median abs. dev. Rfree	0.01	0.01

Data S1. (separate file)
Summary of all collected biochemical data.

Data S2. (separate file)
Summary of designs uploaded to the COVID Moonshot platform.

Data S3. (separate file)
Summary of nanomole high throughput optimization.

Data S4. (separate file)
Summary of crystallographic and refinement statistics

Data S5. (separate file)
Summary of ADMET and PK data.

Data S6. (separate file)
Summary of all cellular anti-viral data produced in this project.

Data S7. (separate file)
The Covid Moonshot consortium member list

References and Notes

1. N. Phillips, The coronavirus is here to stay – Here's what that means. *Nature* **590**, 382–384 (2021). doi:10.1038/d41586-021-00396-2 Medline

2. N. Winchester, "Covid-19 vaccinations: Is the Global South falling behind?" (UK House of Lords Library, 2021); https://lordslibrary.parliament.uk/covid-19-vaccinations-is-the-global-south-falling-behind/.

3. T. Zhao, C. Hu, M. Ayaz Ahmed, C. Cheng, Y. Chen, C. Sun, Warnings regarding the potential coronavirus disease 2019 (COVID-19) transmission risk: Vaccination is not enough. *Infect. Control Hosp. Epidemiol.* **43**, 679–680 (2022). doi:10.1017/ice.2021.63 Medline

4. J. Hammond, H. Leister-Tebbe, A. Gardner, P. Abreu, W. Bao, W. Wisemandle, M. Baniecki, V. M. Hendrick, B. Damle, A. Simón-Campos, R. Pypstra, J. M. Rusnak, EPIC-HR Investigators, Oral Nirmatrelvir for High-Risk, Nonhospitalized Adults with Covid-19. *N. Engl. J. Med.* **386**, 1397–1408 (2022). doi:10.1056/NEJMoa2118542 Medline

5. M. W. McCarthy, Ensitrelvir as a potential treatment for COVID-19. *Expert Opin. Pharmacother.* **23**, 1995–1998 (2022). doi:10.1080/14656566.2022.2146493 Medline

6. A. Jayk Bernal, M. M. Gomes da Silva, D. B. Musungaie, E. Kovalchuk, A. Gonzalez, V. Delos Reyes, A. Martín-Quirós, Y. Caraco, A. Williams-Diaz, M. L. Brown, J. Du, A. Pedley, C. Assaid, J. Strizki, J. A. Grobler, H. H. Shamsuddin, R. Tipping, H. Wan, A. Paschke, J. R. Butterton, M. G. Johnson, C. De Anda, MOVe-OUT Study Group, Molnupiravir for Oral Treatment of Covid-19 in Nonhospitalized Patients. *N. Engl. J. Med.* **386**, 509–520 (2022). doi:10.1056/NEJMoa2116044 Medline

7. E. de Wit, N. van Doremalen, D. Falzarano, V. J. Munster, SARS and MERS: Recent insights into emerging coronaviruses. *Nat. Rev. Microbiol.* **14**, 523–534 (2016). doi:10.1038/nrmicro.2016.81 Medline

8. S. Ullrich, C. Nitsche, The SARS-CoV-2 main protease as drug target. *Bioorg. Med. Chem. Lett.* **30**, 127377 (2020). doi:10.1016/j.bmcl.2020.127377 Medline

9. T. Pillaiyar, M. Manickam, V. Namasivayam, Y. Hayashi, S.-H. Jung, An Overview of Severe Acute Respiratory Syndrome-Coronavirus (SARS-CoV) 3CL Protease Inhibitors: Peptidomimetics and Small Molecule Chemotherapy. *J. Med. Chem.* **59**, 6595–6628 (2016). doi:10.1021/acs.jmedchem.5b01461 Medline

10. L. Zhang, D. Lin, X. Sun, U. Curth, C. Drosten, L. Sauerhering, S. Becker, K. Rox, R. Hilgenfeld, Crystal structure of SARS-CoV-2 main protease provides a basis for design of improved α-ketoamide inhibitors. *Science* **368**, 409–412 (2020). doi:10.1126/science.abb3405 Medline

11. Z. Jin, X. Du, Y. Xu, Y. Deng, M. Liu, Y. Zhao, B. Zhang, X. Li, L. Zhang, C. Peng, Y. Duan, J. Yu, L. Wang, K. Yang, F. Liu, R. Jiang, X. Yang, T. You, X. Liu, X. Yang, F. Bai, H. Liu, X. Liu, L. W. Guddat, W. Xu, G. Xiao, C. Qin, Z. Shi, H. Jiang, Z. Rao, H. Yang, Structure of M^{pro} from SARS-CoV-2 and discovery of its inhibitors. *Nature* **582**, 289–293 (2020). doi:10.1038/s41586-020-2223-y Medline

12. D. R. Owen, C. M. N. Allerton, A. S. Anderson, L. Aschenbrenner, M. Avery, S. Berritt, B. Boras, R. D. Cardin, A. Carlo, K. J. Coffman, A. Dantonio, L. Di, H. Eng, R. Ferre, K. S. Gajiwala, S. A. Gibson, S. E. Greasley, B. L. Hurst, E. P. Kadar, A. S. Kalgutkar, J. C. Lee, J. Lee, W. Liu, S. W. Mason, S. Noell, J. J. Novak, R. S. Obach, K. Ogilvie, N. C. Patel, M. Pettersson, D. K. Rai, M. R. Reese, M. F. Sammons, J. G.

Sathish, R. S. P. Singh, C. M. Steppan, A. E. Stewart, J. B. Tuttle, L. Updyke, P. R. Verhoest, L. Wei, Q. Yang, Y. Zhu, An oral SARS-CoV-2 M^{pro} inhibitor clinical candidate for the treatment of COVID-19. *Science* **374**, 1586–1593 (2021). doi:10.1126/science.abl4784 Medline

13. H. Mukae, H. Yotsuyanagi, N. Ohmagari, Y. Doi, T. Imamura, T. Sonoyama, T. Fukuhara, G. Ichihashi, T. Sanaki, K. Baba, Y. Takeda, Y. Tsuge, T. Uehara, A Randomized Phase 2/3 Study of Ensitrelvir, a Novel Oral SARS-CoV-2 3C-Like Protease Inhibitor, in Japanese Patients with Mild-to-Moderate COVID-19 or Asymptomatic SARS-CoV-2 Infection: Results of the Phase 2a Part. *Antimicrob. Agents Chemother.* **66**, e0069722 (2022). doi:10.1128/aac.00697-22 Medline

14. H. Mukae, H. Yotsuyanagi, N. Ohmagari, Y. Doi, H. Sakaguchi, T. Sonoyama, G. Ichihashi, T. Sanaki, K. Baba, Y. Tsuge, T. Uehara, Efficacy and safety of ensitrelvir in patients with mild-to-moderate Coronavirus Disease 2019: The phase 2b part of a randomized, placebo-controlled, phase 2/3 study. *Clin. Infect. Dis.* **76**, 1403–1411 (2023). doi:10.1093/cid/ciac933 Medline

15. Study of PF-07321332 in Healthy Participants, ClinicalTrials.gov ID NCT04756531 (NIH, 2022); https://clinicaltrials.gov/study/NCT04756531.

16. Study of PBI-0451 in Healthy Subjects, ClinicalTrials.gov ID NCT05011812 (NIH, 2022); https://clinicaltrials.gov/study/NCT05011812.

17. J. Heskin, S. J. C. Pallett, N. Mughal, G. W. Davies, L. S. P. Moore, M. Rayment, R. Jones, Caution required with use of ritonavir-boosted PF-07321332 in COVID-19 management. *Lancet* **399**, 21–22 (2022). doi:10.1016/S0140-6736(21)02657-X Medline

18. F. von Delft, M. Calmiano, J. Chodera, E. Griffen, A. Lee, N. London, T. Matviuk, B. Perry, M. Robinson, A. von Delft, A white-knuckle ride of open COVID drug discovery. *Nature* **594**, 330–332 (2021). doi:10.1038/d41586-021-01571-1 Medline

19. J. Chodera, A. A. Lee, N. London, F. von Delft, Crowdsourcing drug discovery for pandemics. *Nat. Chem.* **12**, 581 (2020). doi:10.1038/s41557-020-0496-2 Medline

20. A. Douangamath, D. Fearon, P. Gehrtz, T. Krojer, P. Lukacik, C. D. Owen, E. Resnick, C. Strain-Damerell, A. Aimon, P. Ábrányi-Balogh, J. Brandão-Neto, A. Carbery, G. Davison, A. Dias, T. D. Downes, L. Dunnett, M. Fairhead, J. D. Firth, S. P. Jones, A. Keeley, G. M. Keserü, H. F. Klein, M. P. Martin, M. E. M. Noble, P. O’Brien, A. Powell, R. N. Reddi, R. Skyner, M. Snee, M. J. Waring, C. Wild, N. London, F. von Delft, M. A. Walsh, Crystallographic and electrophilic fragment screening of the SARS-CoV-2 main protease. *Nat. Commun.* **11**, 5047 (2020). doi:10.1038/s41467-020-18709-w Medline

21. Main protease structure and XChem fragment screen (Diamond Light Source, 2020); https://www.diamond.ac.uk/covid-19/for-scientists/Main-protease-structure-and-XChem.html.

22. XChem @ Diamond; https://fragalysis.diamond.ac.uk/viewer/react/preview/target/Mpro.

23. D. C. Rees, M. Congreve, C. W. Murray, R. Carr, Fragment-based lead discovery. *Nat. Rev. Drug Discov.* **3**, 660–672 (2004). doi:10.1038/nrd1467 Medline

24. D. A. Erlanson, S. W. Fesik, R. E. Hubbard, W. Jahnke, H. Jhoti, Twenty years on: The impact of fragments on drug discovery. *Nat. Rev. Drug Discov.* **15**, 605–619 (2016). doi:10.1038/nrd.2016.109 Medline

25. S. Cooper, F. Khatib, A. Treuille, J. Barbero, J. Lee, M. Beenen, A. Leaver-Fay, D. Baker, Z. Popović, F. Players, Predicting protein structures with a multiplayer online game. *Nature* **466**, 756–760 (2010). doi:10.1038/nature09304 Medline

26. J. Lee, W. Kladwang, M. Lee, D. Cantu, M. Azizyan, H. Kim, A. Limpaecher, S. Gaikwad, S. Yoon, A. Treuille, R. Das, EteRNA Participants, RNA design rules from a massive open laboratory. *Proc. Natl. Acad. Sci. U.S.A.* **111**, 2122–2127 (2014). doi:10.1073/pnas.1313039111 Medline

27. O. B. Cox, T. Krojer, P. Collins, O. Monteiro, R. Talon, A. Bradley, O. Fedorov, J. Amin, B. D. Marsden, J. Spencer, F. von Delft, P. E. Brennan, A poised fragment library enables rapid synthetic expansion yielding the first reported inhibitors of PHIP(2), an atypical bromodomain. *Chem. Sci.* **7**, 2322–2330 (2016). doi:10.1039/C5SC03115J Medline

28. A. Carbery, R. Skyner, F. von Delft, C. M. Deane, Fragment Libraries Designed to Be Functionally Diverse Recover Protein Binding Information More Efficiently Than Standard Structurally Diverse Libraries. *J. Med. Chem.* **65**, 11404–11413 (2022). doi:10.1021/acs.jmedchem.2c01004 Medline

29. N. Kitamura, M. D. Sacco, C. Ma, Y. Hu, J. A. Townsend, X. Meng, F. Zhang, X. Zhang, M. Ba, T. Szeto, A. Kukuljac, M. T. Marty, D. Schultz, S. Cherry, Y. Xiang, Y. Chen, J. Wang, Expedited Approach toward the Rational Design of Noncovalent SARS-CoV-2 Main Protease Inhibitors. *J. Med. Chem.* **65**, 2848–2865 (2022). doi:10.1021/acs.jmedchem.1c00509 Medline

30. D. Zaidman, P. Gehrtz, M. Filep, D. Fearon, R. Gabizon, A. Douangamath, J. Prilusky, S. Duberstein, G. Cohen, C. D. Owen, E. Resnick, C. Strain-Damerell, P. Lukacik, Covid-Moonshot Consortium, H. Barr, M. A. Walsh, F. von Delft, N. London, An automatic pipeline for the design of irreversible derivatives identifies a potent SARS-CoV-2 M^{pro} inhibitor. *Cell Chem. Biol.* **28**, 1795–1806.e5 (2021). doi:10.1016/j.chembiol.2021.05.018 Medline

31. S. H. Han, C. M. Goins, T. Arya, W.-J. Shin, J. Maw, A. Hooper, D. P. Sonawane, M. R. Porter, B. E. Bannister, R. D. Crouch, A. A. Lindsey, G. Lakatos, S. R. Martinez, J. Alvarado, W. S. Akers, N. S. Wang, J. U. Jung, J. D. Macdonald, S. R. Stauffer, Structure-Based Optimization of ML300-Derived, Noncovalent Inhibitors Targeting the Severe Acute Respiratory Syndrome Coronavirus 3CL Protease (SARS-CoV-2 $3CL^{pro}$). *J. Med. Chem.* **65**, 2880–2904 (2022). doi:10.1021/acs.jmedchem.1c00598 Medline

32. P. Schwaller, T. Laino, T. Gaudin, P. Bolgar, C. A. Hunter, C. Bekas, A. A. Lee, Molecular Transformer: A Model for Uncertainty-Calibrated Chemical Reaction Prediction. *ACS Cent. Sci.* **5**, 1572–1583 (2019). doi:10.1021/acscentsci.9b00576 Medline

33. A. Morris, W. McCorkindale, T. C. M. Consortium, N. Drayman, J. D. Chodera, S. Tay, N. London, A. A. Lee, Discovery of SARS-CoV-2 main protease inhibitors using a synthesis-directed *de novo* design model. *Chem. Commun.* **57**, 5909–5912 (2021). doi:10.1039/D1CC00050K Medline

34. A. S. J. S. Mey, B. Allen, H. E. Bruce Macdonald, J. D. Chodera, M. Kuhn, J. Michel, D. L. Mobley, L. N. Naden, S. Prasad, A. Rizzi, J. Scheen, M. R. Shirts, G. Tresadern, H. Xu, Best Practices for Alchemical Free Energy Calculations. arXiv:2008.03067 [q-bio.BM] (2020).

35. R. Abel, L. Wang, D. L. Mobley, R. A. Friesner, A Critical Review of Validation, Blind Testing, and Real- World Use of Alchemical Protein-Ligand Binding Free Energy Calculations. *Curr. Top. Med. Chem.* **17**, 2577–2585 (2017). doi:10.2174/1568026617666170414142131 Medline

36. K. A. Armacost, S. Riniker, Z. Cournia, Exploring Novel Directions in Free Energy Calculations. *J. Chem. Inf. Model.* **60**, 5283–5286 (2020). doi:10.1021/acs.jcim.0c01266 Medline

37. M. Shirts, V. S. Pande, Screen Savers of the World Unite! *Science* **290**, 1903–1904 (2000). doi:10.1126/science.290.5498.1903 Medline

38. M. I. Zimmerman, J. R. Porter, M. D. Ward, S. Singh, N. Vithani, A. Meller, U. L. Mallimadugula, C. E. Kuhn, J. H. Borowsky, R. P. Wiewiora, M. F. D. Hurley, A. M. Harbison, C. A. Fogarty, J. E. Coffland, E. Fadda, V. A. Voelz, J. D. Chodera, G. R. Bowman, SARS-CoV-2 simulations go exascale to predict dramatic spike opening and cryptic pockets across the proteome. *Nat. Chem.* **13**, 651–659 (2021). doi:10.1038/s41557-021-00707-0 Medline

39. Y. Qiu, D. Smith, S. Boothroyd, H. Jang, J. Wagner, C. C. Bannan, T. Gokey, V. T. Lim, C. Stern, A. Rizzi, X. Lucas, B. Tjanaka, M. R. Shirts, M. Gilson, J. Chodera, C. I. Bayly, D. Mobley, L.-P. Wang, Development and Benchmarking of Open Force Field v1.0.0, the Parsley Small Molecule Force Field, ChemRxiv [Preprint] (2020); https://doi.org/10.26434/chemrxiv-2021-l070l-v4.

40. D. A. Rufa, H. E. Bruce Macdonald, J. Fass, M. Wieder, P. B. Grinaway, A. E. Roitberg, O. Isayev, J. D. Chodera, Towards chemical accuracy for alchemical free energy calculations with hybrid physics-based machine learning / molecular mechanics potentials. bioRxiv 2020.07.29.227959 [Preprint] (2020); https://doi.org/10.1101/2020.07.29.227959.

41. D. A. Rufa, I. Zhang, H. E. Bruce Macdonald, P. B. Grinaway, I. Pulido, M. M. Henry, J. Rodríguez-Guerra, M. Wittmann, S. K. Albanese, W. G. Glass, A. Silveira, D. Schaller, L. N. Naden, J. D. Chodera, Perses, version 0.10.2, Zenodo (2023); https://doi.org/10.5281/zenodo.8092391.

42. I. Zhang, D. A. Rufa, I. Pulido, M. M. Henry, L. E. Rosen, K. Hauser, S. Singh, J. D. Chodera, Identifying and Overcoming the Sampling Challenges in Relative Binding Free Energy Calculations of a Model Protein:Protein Complex. *J. Chem. Theory Comput.* **19**, 4863–4882 (2023). doi:10.1021/acs.jctc.3c00333 Medline

43. P. Eastman, J. Swails, J. D. Chodera, R. T. McGibbon, Y. Zhao, K. A. Beauchamp, L.-P. Wang, A. C. Simmonett, M. P. Harrigan, C. D. Stern, R. P. Wiewiora, B. R. Brooks, V. S. Pande, OpenMM 7: Rapid development of high performance algorithms for molecular dynamics. *PLOS Comput. Biol.* **13**, e1005659 (2017). doi:10.1371/journal.pcbi.1005659 Medline

44. A. Buitrago Santanilla, E. L. Regalado, T. Pereira, M. Shevlin, K. Bateman, L.-C. Campeau, J. Schneeweis, S. Berritt, Z.-C. Shi, P. Nantermet, Y. Liu, R. Helmy, C. J. Welch, P. Vachal, I. W. Davies, T. Cernak, S. D. Dreher, Organic chemistry. Nanomole-scale high-throughput chemistry for the synthesis of complex molecules. *Science* **347**, 49–53 (2015). doi:10.1126/science.1259203 Medline

45. D. Perera, J. W. Tucker, S. Brahmbhatt, C. J. Helal, A. Chong, W. Farrell, P. Richardson, N. W. Sach, A platform for automated nanomole-scale reaction screening and micromole-scale synthesis in flow. *Science* **359**, 429–434 (2018). doi:10.1126/science.aap9112 Medline

46. N. J. Gesmundo, B. Sauvagnat, P. J. Curran, M. P. Richards, C. L. Andrews, P. J. Dandliker, T. Cernak, Nanoscale synthesis and affinity ranking. *Nature* **557**, 228–232 (2018). doi:10.1038/s41586-018-0056-8 Medline

47. R. P. Thomas, R. E. Heap, F. Zappacosta, E. K. Grant, P. Pogány, S. Besley, D. J. Fallon, M. M. Hann, D. House, N. C. O. Tomkinson, J. T. Bush, A direct-to-biology high-throughput chemistry approach to reactive fragment screening. *Chem. Sci.* **12**, 12098–12106 (2021). doi:10.1039/D1SC03551G Medline

48. P. Gehrtz, S. Marom, M. Bührmann, J. Hardick, S. Kleinbölting, A. Shraga, C. Dubiella, R. Gabizon, J. N. Wiese, M. P. Müller, G. Cohen, I. Babaev, K. Shurrush, L. Avram, E. Resnick, H. Barr, D. Rauh, N. London, Optimization of Covalent MKK7 Inhibitors *via* Crude Nanomole-Scale Libraries. *J. Med. Chem.* **65**, 10341–10356 (2022). doi:10.1021/acs.jmedchem.1c02206 Medline

49. J.-Q. Chen, J.-H. Li, Z.-B. Dong, A Review on the Latest Progress of Chan-Lam Coupling Reaction. *Adv. Synth. Catal.* **362**, 3311–3331 (2020). doi:10.1002/adsc.202000495

50. E. Resnick, A. Bradley, J. Gan, A. Douangamath, T. Krojer, R. Sethi, P. P. Geurink, A. Aimon, G. Amitai, D. Bellini, J. Bennett, M. Fairhead, O. Fedorov, R. Gabizon, J. Gan, J. Guo, A. Plotnikov, N. Reznik, G. F. Ruda, L. Díaz-Sáez, V. M. Straub, T. Szommer, S. Velupillai, D. Zaidman, Y. Zhang, A. R. Coker, C. G. Dowson, H. M. Barr, C. Wang, K. V. M. Huber, P. E. Brennan, H. Ovaa, F. von Delft, N. London, Rapid Covalent-Probe Discovery by Electrophile-Fragment Screening. *J. Am. Chem. Soc.* **141**, 8951–8968 (2019). doi:10.1021/jacs.9b02822 Medline

51. M. N. L. Nalam, A. Ali, M. D. Altman, G. S. K. K. Reddy, S. Chellappan, V. Kairys, A. Ozen, H. Cao, M. K. Gilson, B. Tidor, T. M. Rana, C. A. Schiffer, Evaluating the substrate-envelope hypothesis: Structural analysis of novel HIV-1 protease inhibitors designed to be robust against drug resistance. *J. Virol.* **84**, 5368–5378 (2010). doi:10.1128/JVI.02531-09 Medline

52. J. Jansen, K. C. Reimer, J. S. Nagai, F. S. Varghese, G. J. Overheul, M. de Beer, R. Roverts, D. Daviran, L. A. S. Fermin, B. Willemsen, M. Beukenboom, S. Djudjaj, S. von Stillfried, L. E. van Eijk, M. Mastik, M. Bulthuis, W. den Dunnen, H. van Goor, J.-L. Hillebrands, S. H. Triana, T. Alexandrov, M.-C. Timm, B. T. van den Berge, M. van den Broek, Q. Nlandu, J. Heijnert, E. M. J. Bindels, R. M. Hoogenboezem, F. Mooren, C. Kuppe, P. Miesen, K. Grünberg, T. Ijzermans, E. J. Steenbergen, J. Czogalla, M. F. Schreuder, N. Sommerdijk, A. Akiva, P. Boor, V. G. Puelles, J. Floege, T. B. Huber, COVID Moonshot consortium, R. P. van Rij, I. G. Costa, R. K. Schneider, B. Smeets, R. Kramann, SARS-CoV-2 infects the human kidney and drives fibrosis in kidney organoids. *Cell Stem Cell* **29**, 217–231.e8 (2021). doi:10.1016/j.stem.2021.12.010 Medline

53. Y. Unoh, S. Uehara, K. Nakahara, H. Nobori, Y. Yamatsu, S. Yamamoto, Y. Maruyama, Y. Taoda, K. Kasamatsu, T. Suto, K. Kouki, A. Nakahashi, S. Kawashima, T. Sanaki, S. Toba, K. Uemura, T. Mizutare, S. Ando, M. Sasaki, Y. Orba, H. Sawa, A. Sato, T. Sato, T. Kato, Y. Tachibana, Discovery of S-217622, a Noncovalent Oral SARS-CoV-2 3CL Protease Inhibitor Clinical Candidate for Treating COVID-19. *J. Med. Chem.* **65**, 6499–6512 (2022). doi:10.1021/acs.jmedchem.2c00117 Medline

54. "COVID Moonshot funded by COVID-19 Therapeutics Accelerator to rapidly develop a safe, globally accessible and affordable antiviral pill," Drugs for Neglected Diseases Initiative (DNDi) (2021); https://dndi.org/press-releases/2021/covid-moonshot-

funded-by-wellcome-to-rapidly-develop-safe-globally-accessible-affordable-antiviral-pill/.

55. J. Strovel, S. Sittampalam, N. P. Coussens, M. Hughes, J. Inglese, A. Kurtz, A. Andalibi, L. Patton, C. Austin, M. Baltezor, M. Beckloff, M. Weingarten, S. Weir, "Early Drug Discovery and Development Guidelines: For Academic Researchers, Collaborators, and Start-up Companies," in *Assay Guidance Manual*, S. Markossian, A. Grossman, K. Brimacombe, M. Arkin, D. Auld, C. Austin, J. Baell, T. D. Y. Chung, N. P. Coussens, J. L. Dahlin, V. Devanarayan, T. L. Foley, M. Glicksman, K. Gorshkov, J. V. Haas, M. D. Hall, S. Hoare, J. Inglese, P. W. Iversen, S. C. Kales, M. Lal-Nag, Z. Li, J. McGee, O. McManus, T. Riss, P. Saradjian, G. S. Sittampalam, M. Tarselli, O. J. Trask Jr., Y. Wang, J. R. Weidner, M. J. Wildey, K. Wilson, M. Xia, X. Xu, Eds. (Eli Lilly & Company and the National Center for Advancing Translational Sciences, 2016).

56. International Human Genome Sequencing Consortium, Initial sequencing and analysis of the human genome. *Nature* **409**, 860–921 (2001). doi:10.1038/35057062 Medline

57. A. R. Williamson, Creating a structural genomics consortium. *Nat. Struct. Biol.* **7**, 953 (2000). doi:10.1038/80726 Medline

58. J. Kaiser, "U.S. Cancer Institute 'Megaproject' to Target Common Cancer-Driving Protein," *ScienceInsider*, 24 June 2013; https://www.science.org/content/article/us-cancer-institute-megaproject-target-common-cancer-driving-protein.

59. A. Lee, J. Chodera, F. von Delft, "Why we are developing a patent-free Covid antiviral therapy," *Knowable Magazine*, 27 September 2021; https://doi.org/10.1146/knowable-092721-1.

60. S. Legare, F. Heide, B. A. Bailey-Elkin, J. Stetefeld, Improved SARS-CoV-2 main protease high-throughput screening assay using a 5-carboxyfluorescein substrate. *J. Biol. Chem.* **298**, 101739 (2022). doi:10.1016/j.jbc.2022.101739 Medline

61. M. G. Acker, D. S. Auld, Considerations for the design and reporting of enzyme assays in high-throughput screening applications. *Perspect. Sci.* **1**, 56–73 (2014). doi:10.1016/j.pisc.2013.12.001

62. T. R. Malla, A. Tumber, T. John, L. Brewitz, C. Strain-Damerell, C. D. Owen, P. Lukacik, H. T. H. Chan, P. Maheswaran, E. Salah, F. Duarte, H. Yang, Z. Rao, M. A. Walsh, C. J. Schofield, Mass spectrometry reveals potential of β-lactams as SARS-CoV-2 M^{pro} inhibitors. *Chem. Commun.* **57**, 1430–1433 (2021). doi:10.1039/D0CC06870E Medline

63. G. D. Noske, A. M. Nakamura, V. O. Gawriljuk, R. S. Fernandes, G. M. A. Lima, H. V. D. Rosa, H. D. Pereira, A. C. M. Zeri, A. F. Z. Nascimento, M. C. L. C. Freire, D. Fearon, A. Douangamath, F. von Delft, G. Oliva, A. S. Godoy, A Crystallographic Snapshot of SARS-CoV-2 Main Protease Maturation Process. *J. Mol. Biol.* **433**, 167118 (2021). doi:10.1016/j.jmb.2021.167118 Medline

64. G. Winter, D. G. Waterman, J. M. Parkhurst, A. S. Brewster, R. J. Gildea, M. Gerstel, L. Fuentes-Montero, M. Vollmar, T. Michels-Clark, I. D. Young, N. K. Sauter, G. Evans, *DIALS*: Implementation and evaluation of a new integration package. *Acta Cryst.* **D74**, 85–97 (2018). doi:10.1107/S2059798317017235 Medline

65. G. Winter, R. J. Gildea, N. G. Paterson, J. Beale, M. Gerstel, D. Axford, M. Vollmar, K. E. McAuley, R. L. Owen, R. Flaig, A. W. Ashton, D. R. Hall, How best to use photons. *Acta Cryst.* **D75**, 242–261 (2019). doi:10.1107/S2059798319003528 Medline

66. G. Winter, K. E. McAuley, Automated data collection for macromolecular crystallography. *Methods* **55**, 81–93 (2011). doi:10.1016/j.ymeth.2011.06.010 Medline

67. W. Kabsch, Integration, scaling, space-group assignment and post-refinement. *Acta Cryst.* **D66**, 133–144 (2010). doi:10.1107/S0907444909047374 Medline

68. G. Winter, C. M. C. Lobley, S. M. Prince, Decision making in *xia*2. *Acta Cryst.* **D69**, 1260–1273 (2013). doi:10.1107/S0907444913015308 Medline

69. C. Vonrhein, C. Flensburg, P. Keller, A. Sharff, O. Smart, W. Paciorek, T. Womack, G. Bricogne, Data processing and analysis with the *autoPROC* toolbox. *Acta Cryst.* **D67**, 293–302 (2011). doi:10.1107/S0907444911007773 Medline

70. T. Krojer, R. Talon, N. Pearce, P. Collins, A. Douangamath, J. Brandao-Neto, A. Dias, B. Marsden, F. von Delft, The *XChemExplorer* graphical workflow tool for routine or large-scale protein–ligand structure determination. *Acta Cryst.* **D73**, 267–278 (2017). doi:10.1107/S2059798316020234 Medline

71. N. M. Pearce, T. Krojer, A. R. Bradley, P. Collins, R. P. Nowak, R. Talon, B. D. Marsden, S. Kelm, J. Shi, C. M. Deane, F. von Delft, A multi-crystal method for extracting obscured crystallographic states from conventionally uninterpretable electron density. *Nat. Commun.* **8**, 15123 (2017). doi:10.1038/ncomms15123 Medline

72. P. Emsley, B. Lohkamp, W. G. Scott, K. Cowtan, Features and development of *Coot*. *Acta Cryst.* **D66**, 486–501 (2010). doi:10.1107/S0907444910007493 Medline

73. F. Long, R. A. Nicholls, P. Emsley, S. Gražulis, A. Merkys, A. Vaitkus, G. N. Murshudov, *AceDRG*: A stereochemical description generator for ligands. *Acta Cryst.* **D73**, 112–122 (2017). doi:10.1107/S2059798317000067 Medline

74. T. Gorrie-Stone, xchem/XChemReview: Jan_2021, Zenodo (2022); https://doi.org/10.5281/zenodo.5939113.

75. I. J. Bruno, J. C. Cole, M. Kessler, J. Luo, W. D. S. Motherwell, L. H. Purkis, B. R. Smith, R. Taylor, R. I. Cooper, S. E. Harris, A. G. Orpen, Retrieval of crystallographically-derived molecular geometry information. *J. Chem. Inf. Comput. Sci.* **44**, 2133–2144 (2004). doi:10.1021/ci049780b Medline

76. S. J. Cottrell, T. S. G. Olsson, R. Taylor, J. C. Cole, J. W. Liebeschuetz, Validating and understanding ring conformations using small molecule crystallographic data. *J. Chem. Inf. Model.* **52**, 956–962 (2012). doi:10.1021/ci200439d Medline

77. V. M. Corman, O. Landt, M. Kaiser, R. Molenkamp, A. Meijer, D. K. Chu, T. Bleicker, S. Brünink, J. Schneider, M. L. Schmidt, D. G. Mulders, B. L. Haagmans, B. van der Veer, S. van den Brink, L. Wijsman, G. Goderski, J.-L. Romette, J. Ellis, M. Zambon, M. Peiris, H. Goossens, C. Reusken, M. P. Koopmans, C. Drosten, Detection of 2019 novel coronavirus (2019-nCoV) by real-time RT-PCR. *Euro Surveill.* **25**, 2000045 (2020). doi:10.2807/1560-7917.ES.2020.25.3.2000045 Medline

78. F. S. Varghese, E. van Woudenbergh, G. J. Overheul, M. J. Eleveld, L. Kurver, N. van Heerbeek, A. van Laarhoven, P. Miesen, G. den Hartog, M. I. de Jonge, R. P. van Rij, Berberine and Obatoclax Inhibit SARS-Cov-2 Replication in Primary Human Nasal Epithelial Cells In Vitro. *Viruses* **13**, 282 (2021). doi:10.3390/v13020282 Medline

79. D. Jochmans, P. Leyssen, J. Neyts, A novel method for high-throughput screening to quantify antiviral activity against viruses that induce limited CPE. *J. Virol. Methods* **183**, 176–179 (2012). doi:10.1016/j.jviromet.2012.04.011 Medline

80. R. Rosales, B. L. McGovern, M. Luis Rodriguez, D. K. Rai, R. D. Cardin, A. S. Anderson, PSP study group, E. M. Sordillo, H. van Bakel, V. Simon, A. García-Sastre, K. M. White, Nirmatrelvir, Molnupiravir, and Remdesivir maintain potent in vitro activity against the SARS-CoV-2 Omicron variant. bioRxiv 2022.01.17.476685 [Preprint] (2022); https://doi.org/10.1101/2022.01.17.476685.

81. V. Gapsys, L. Pérez-Benito, M. Aldeghi, D. Seeliger, H. van Vlijmen, G. Tresadern, B. L. de Groot, Large scale relative protein ligand binding affinities using non-equilibrium alchemy. *Chem. Sci.* **11**, 1140–1152 (2020). doi:10.1039/C9SC03754C Medline

82. J. A. Maier, C. Martinez, K. Kasavajhala, L. Wickstrom, K. E. Hauser, C. Simmerling, ff14SB: Improving the Accuracy of Protein Side Chain and Backbone Parameters from ff99SB. *J. Chem. Theory Comput.* **11**, 3696–3713 (2015). doi:10.1021/acs.jctc.5b00255 Medline

83. I. S. Joung, T. E. Cheatham 3rd, Determination of alkali and halide monovalent ion parameters for use in explicitly solvated biomolecular simulations. *J. Phys. Chem. B* **112**, 9020–9041 (2008). doi:10.1021/jp8001614 Medline

84. I. S. Joung, T. E. Cheatham 3rd, Molecular dynamics simulations of the dynamic and energetic properties of alkali and halide ions using water-model-specific ion parameters. *J. Phys. Chem. B* **113**, 13279–13290 (2009). doi:10.1021/jp902584c Medline

85. W. L. Jorgensen, J. Chandrasekhar, J. D. Madura, R. W. Impey, M. L. Klein, Comparison of simple potential functions for simulating liquid water. *J. Chem. Phys.* **79**, 926–935 (1983). doi:10.1063/1.445869

86. H. Xu, Optimal Measurement Network of Pairwise Differences. *J. Chem. Inf. Model.* **59**, 4720–4728 (2019). doi:10.1021/acs.jcim.9b00528

87. G. E. Crooks, Path-ensemble averages in systems driven far from equilibrium. *Phys. Rev. E* **61**, 2361–2366 (2000). doi:10.1103/PhysRevE.61.2361

88. C. H. Bennett, Efficient estimation of free energy differences from Monte Carlo data. *J. Comput. Phys.* **22**, 245–268 (1976). doi:10.1016/0021-9991(76)90078-4

89. S. Salentin, S. Schreiber, V. J. Haupt, M. F. Adasme, M. Schroeder, PLIP: Fully automated protein–ligand interaction profiler. *Nucleic Acids Res.* **43**, W443–W447 (2015). doi:10.1093/nar/gkv315 Medline

90. D. W. Kneller, G. Phillips, H. M. O'Neill, R. Jedrzejczak, L. Stols, P. Langan, A. Joachimiak, L. Coates, A. Kovalevsky, Structural plasticity of SARS-CoV-2 3CL M^{pro} active site cavity revealed by room temperature X-ray crystallography. *Nat. Commun.* **11**, 3202 (2020). doi:10.1038/s41467-020-16954-7 Medline

91. M. Robinson, M. Henry, A. Morris, asapdiscovery/COVID_moonshot_submissions: Initial release for zenodo, version v0.1, Zenodo (2023); https://doi.org/10.5281/zenodo.8196348.

92. J. Scheen, asapdiscovery/COVID_moonshot_FECs_data: First Release, version 1.0.0, Zenodo (2023); https://doi.org/10.5281/zenodo.8215553.

93. D. Fearon, A. Aimon, J. C. Aschenbrenner, B. H. Balcomb, I. A. Barker, F. K. R. Bertram, J. Brandao-Neto, A. Dias, A. Douangamath, L. Dunnett, A. S. Godoy, T. J. Gorrie-Stone, L. Koekemoer, T. Krojer, R. M. Lithgo, P. Lukacik, P. G. Marples, H. Mikolajek, E. Nelson, K. H. V. Nidamarthi, C. D. Owen, A. J. Powell, V. L. Rangel, R. Skyner, C. M. Strain-Damerell, W. Thompson, C. W. E. Tomlinson, C. Wild, M. A. Walsh, F. von Delft, Crystal structures of SARS-CoV-2 main protease screened

against COVID Moonshot compounds by X-ray Crystallography at the XChem facility of Diamond Light Source, data set, Zenodo (2023); https://doi.org/10.5281/zenodo.8214528.

94. J. Jiménez-Luna, F. Grisoni, G. Schneider, Drug discovery with explainable artificial intelligence. *Nat. Mach. Intell.* **2**, 573–584 (2020). doi:10.1038/s42256-020-00236-4

95. A. Özen, W. Sherman, C. A. Schiffer, Improving the Resistance Profile of Hepatitis C NS3/4A Inhibitors: Dynamic Substrate Envelope Guided Design. *J. Chem. Theory Comput.* **9**, 5693–5705 (2013). doi:10.1021/ct400603p Medline
